# The future of the Arctic flora under climate change

**DOI:** 10.1093/nsr/nwag096

**Published:** 2026-02-11

**Authors:** Jun Zhang, Huan-Wen Peng, Hai-Tao Ding, Guoke Chen, Andrey S Erst, Jin-Feng Li, Lian Lian, Fu-Cai Xia, Xiaoqi Zhou, Robert A Spicer, Jian Yang, Wei Wang

**Affiliations:** State Key Laboratory of Plant Diversity and Specialty Crops, Institute of Botany, Chinese Academy of Sciences, China; University of Chinese Academy of Sciences, China; China National Botanical Garden, China; State Key Laboratory of Plant Diversity and Specialty Crops, Institute of Botany, Chinese Academy of Sciences, China; China National Botanical Garden, China; Antarctic Great Wall Ecology National Observation and Research Station, Polar Research Institute of China, Ministry of Natural Resources, China; State Key Laboratory of Vegetation and Environmental Change, Institute of Botany, Chinese Academy of Sciences, China; Central Siberian Botanical Garden, Siberian Branch of Russian Academy of Sciences, Russia; State Key Laboratory of Plant Diversity and Specialty Crops, Institute of Botany, Chinese Academy of Sciences, China; University of Chinese Academy of Sciences, China; China National Botanical Garden, China; State Key Laboratory of Plant Diversity and Specialty Crops, Institute of Botany, Chinese Academy of Sciences, China; China National Botanical Garden, China; Forestry College, Beihua University, China; Zhejiang Tiantong Forest Ecosystem National Observation and Research Station, Zhejiang Zhoushan Island Ecosystem Observation and Research Station, School of Ecological and Environmental Sciences, East China Normal University, China; School of Environment, Earth and Ecosystem Sciences, The Open University, UK; Yunnan Key Laboratory of Forest Ecosystem Stability and Global Change, Xishuangbanna Tropical Botanical Garden, Chinese Academy of Sciences, China; State Key Laboratory of Tibetan Plateau Earth System, Resources and Environment (TPESRE), Institute of Tibetan Plateau Research, Chinese Academy of Sciences, China; State Key Laboratory of Plant Diversity and Specialty Crops, Institute of Botany, Chinese Academy of Sciences, China; University of Chinese Academy of Sciences, China; China National Botanical Garden, China; State Key Laboratory of Plant Diversity and Specialty Crops, Institute of Botany, Chinese Academy of Sciences, China; University of Chinese Academy of Sciences, China; China National Botanical Garden, China

The Arctic flora occupies a narrow zone between the natural tree line and the Arctic Ocean, and is highly sensitive to changes in global climate [1]. In recent decades, this region has been warming at rates 3–4 times the global average [2], a phenomenon known as Arctic amplification [3]. This rapid warming will cause permafrost thaw and methane release and alter atmospheric and oceanic circulation [4]. By the end of the 21st century, the mean surface temperature over the Arctic could be ∼2.8°C–10.4°C warmer than now depending on which greenhouse gas (GHG) emission scenarios play out (from SSP1-2.6 to SSP5-8.5) [5]. The magnitude and rate of this projected future climate change are unparalleled in the past several million years [6], underscoring the urgency of predicting potential changes in the Arctic flora under different emission scenarios. Climate change will cause species to shift, contract and/or expand their ranges [7], and consequently alter community composition, species richness patterns and thus ecosystem functioning [8]. The Arctic flora is home to 2041 vascular plant species that are tolerant of low temperatures, frozen soils, desiccation and short reproductive windows [9] (Fig. S1). To date, only small-scale species modelling limited to relatively few taxa has been conducted [10–12], which precludes broad understanding of the details and magnitude of that change, and responses of the Arctic flora as a whole.

Based on temporal and spatial differences in past glaciations, land bridges and north-south trending mountain ranges, the Arctic flora may be divided into five sectors: European Russia-West Siberia (ER-WSS), East Siberia (ESS), Beringia (BS), Canada (CS) and North Atlantic (NAS) sectors [13] (Fig. 1a). Spatial heterogeneity and complexity in Arctic vegetation responses to climate change are already being observed [14]. Modelling studies involving part sectors, such as northernmost Europe and northwestern North America [11,12], show the likely range loss among most of the studied species, but complex responses in terms of richness. Though poleward range shifts are most frequently reported [7,11], non-poleward shifts have also been detected in northernmost Europe [12]. We do not yet know whether, or how, responses in the five sectors will differ under future emission scenarios, hence this study.

**Figure 1.**
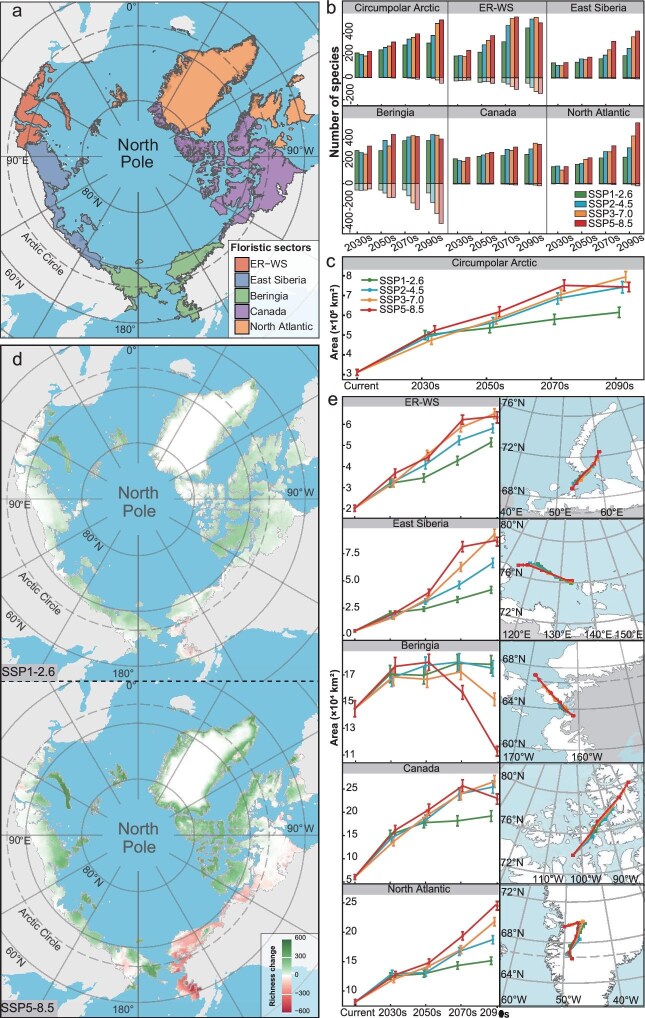
Potential changes in the Arctic flora and its five sectors from now until 2100 under different emission scenarios. (a) The Arctic and its five sectors. (b) Number of species in the Arctic with expansion (positive values) and contraction (negative values) in the AOH compared with the present. (c) Mean AOH changes of species in the Arctic now and in the future. (d) Potential species richness changes by the 2090s under different climate scenarios compared with now. (e) Mean AOH changes of species and mean distributional centroid changes in spatiotemporal species patterns in the five sectors now and in the future. Arrows indicate the direction and magnitude of predicted species’ mean distributional centroid change through time.

To test the hypothesis of a heterogeneous spatial and temporal floral response to future Arctic warming, we assembled occurrence records of all 2041 Arctic vascular plant species and retained 1187 (∼58.2%) with more than 25 occurrences for subsequent analyses, which ensures higher model predictive power (Data S1). Our floristic-level modelling covers ∼94.5% (86/91) and ∼76.2% (325/426) of Arctic vascular plant families and genera, respectively (Fig. S2). We incorporate outcomes from five different modelling

techniques to predict the area of habitat (AOH) for each of the sampled species, and then generate species richness maps for the current and future (2030s, 2050s, 2070s and 2090s) conditions under four emission scenarios (from low to high: SSP1-2.6, SSP2-4.5, SSP3-7.0 and SSP5-8.5) for the entire Arctic, as well as each of the five sectors. To examine within-sector change, we calculate the direction and magnitude of the mean distributional centroid in each sector and compare the centroids for current and future binary distribution maps of all sampled species. Our results show widespread but heterogeneous changes in the Arctic flora throughout the 21st century, including surprising cases of southward migration and a halt in richness gains under high emissions. Importantly, future changes in the Arctic flora will be amplified over time as GHG emissions increase.

We generate 20 179 potential distribution maps for the 1187 sampled species with high area under the curve (AUC) values, indicating excellent predictive performance (Data S1). From these, potential species richness distribution maps, species AOH changes and spatiotemporal patterns of species richness changes in the Arctic are obtained (Fig. 1b–d, Figs S3–S20, Table S3), reflecting Arctic floristic responses to the different climate scenarios. Except for relatively stable species whose AOH changes are ≤5%, almost all Arctic species expand their AOHs (90.6%–99.3%) and between 0.7% and 9.4% of species’ AOHs contract, depending on the emission scenario (Fig. 1b, Table S1, Data S2). Across the Arctic, there is a mean AOH expansion of 3.04–4.83 × 10^5^ km^2^ (0.5–1.6 times) between now and the 2090s (Fig. 1c, Table S2). The Arctic potential mean

species richness (MSR) is currently 52.4 species per grid cell (spgc), with projections indicating an increase of 27.4–81.0 spgc (Fig. S20, Table S3). Potential MSR change trends follow those of the AOHs (Fig. 1c, Fig. S20), supporting a positive relationship between species richness and spatial range [15]. Species richness is expected to increase significantly in the northern Arctic, while decreasing in the south (Fig. 1d and Figs S21–S32), which may lead to site-specific changes in species composition.

The responses of five sectors to future climate change are heterogeneous (Fig. 1b, d and e, Figs S20–S32). Although most species are expected to expand their AOHs, the number of species with reduced AOHs is higher in the ER-WSS (21–142 species) and BS (49–361 species) (Fig. 1b, Table S4, Data S3). There are also some differences in mean AOH change across the five sectors (Fig. 1e, Table S5). Except for the BS (−0.2 to 0.2 times), the mean AOH in others will increase substantially (ER-WSS: 0.6–2.2 times, ESS: 3.1–21.8 times, CS: 1.3–3.4 times, and NAS: 0.5–2.0 times) (Fig. 1e, Table S5). Currently, the BS exhibits the highest potential MSR (190.1 spgc), followed by the ER-WSS (41.4 spgc), NAS (36.7 spgc), CS (34.4 spgc) and ESS (5.6 spgc) (Fig. S3, Table S6). In future, the MSR of the BS is projected to decline, while other sectors are generally expected to increase under all four emission scenarios (Figs S20–S32, Table S6). Compared to the NAS, the larger areas of the other four sectors will accommodate greater changes in species richness (Fig. 1d, Figs S33–S37). Although the total number of Arctic plant species will not decrease significantly, extinction risks within specific sectors will increase (Table S7). Compared to now, the mean northward migration distance for the five sectors will be 0.5°–3.5°(ER-WSS), −0.3° to 1.3°(ESS), 0.5°–3.8° (BS), 1.7°–6.0° (CS) and 0.4°–3.6° (NAS), and the longitudinal shifts also vary between different sectors and time periods (Fig. 1e, Table S8). Different landscapes, soil conditions, ecological disturbances and especially the direction and magnitude of climate change will likely produce heterogeneous responses among the five sectors [5]. When individual species are analyzed, we see significant differences in both the direction and magnitude of movement (Figs S38–S41, Table S8). Most Arctic species are projected to migrate northward (65.5%–93.8% in five sectors), but some undergo southward migration (6.2%–34.5%, Figs S38–S41, Table S9), demonstrating species-specific responses.

Potential changes of the Arctic flora will be amplified greatly in future as GHG emissions increase. Under the higher emission scenarios (SSP3-7.0 and SSP5-8.5), the Arctic flora will undergo more profound transformations (Fig. 1b–e). The mean AOH for species in some sectors will shrink, particularly in the BS (Fig. 1e, Table S5). Moreover, the increase in MSR and the northward shift evident in some sectors (i.e. the CS, ESS and NAS) are expected to slow or even stop (Fig. 1e, Tables S6 and S8). Importantly, our projections show that potential responses of the Arctic flora spanning lower and higher GHG emission scenarios are temporally displaced (Fig. 1, Tables S2, S3, S5, S6 and S8). Only if mean global warming is limited to below 2°C, a low-emission future (SSP1-2.6), will potential changes in the Arctic flora be manageable (Fig. 1b–e). Around the 2070s, possibly earlier, both species AOH and MSR decline, with northward migration decelerating in CS and potentially halting in the ESS and NAS (Fig. 1e, Fig. S20). While there are no major differences in potential changes in the short term (from now to the 2050s), the magnitudes of risks under the four emission scenarios will grow substantially over time (Fig. 1c and e). Continued GHG emissions, combined with other potential stressors (e.g. changes in sea ice and permafrost), will likely accelerate Arctic floral change.

Instead of the smaller-scale selective studies so far undertaken, our study predicts the likely future development of the Arctic flora by including almost all of the current taxa. The intrinsic uncertainties of species distribution models [16], and biotic (e.g. species-specific dispersal limitations and interspecific competition) and abiotic factors (e.g. permafrost, snow cover and topography) can influence the prediction accuracy [17], which future work needs to consider, but our comprehensive approach is flexible, scalable and can be applied to any regional flora. Our results show that the responses of the Arctic flora to climate change are heterogeneous, complex and will be amplified over time as GHG emissions increase. Arctic species currently occupying the northernmost regions of the continent will find themselves in a ‘nowhere-to-go’ situation and become extinct. Importantly, we demonstrate that a low-emission future (SSP1-2.6) could allow for effective conservation of the Arctic flora, albeit with much change already ‘baked in’ due to natural response times. The increasing risks to the Arctic flora inherent in other emission scenarios will have profound implications for global vegetation-atmosphere interactions and become an important source of uncertainty for carbon uptake in the terrestrial biosphere. This underscores the urgency of developing strategies to mitigate and minimize the impacts of GHG emissions, rapid implementations of which are crucial for promoting sustainable development in the Arctic.

## MATERIALS AND METHODS

For Materials and methods, please refer to the Supplementary data.

## Supplementary Material

nwag096_Supplemental_File

## Data Availability

A total of 20 179 potential suitable distribution maps of 1187 species in different periods and scenarios and Data S1–S3 are openly available in Zenodo at https://doi.org/10.5281/zenodo.16946158. Source data are provided in the Supplementary data.

## References

[1] Seddon AWR, Macias-Fauria M, Long PR et al. Nature 2016; 531: 229–32. 10.1038/nature16986 26886790

[2] Zhou W, Leung LR, Lu J. Nat Geosci 2024; 17: 508–15. 10.1038/s41561-024-01441-1

[3] Cohen J, Screen JA, Furtado JC et al. Nat Geosci 2014; 7: 627–37. 10.1038/ngeo2234

[4] Schuur EAG, Abbott BW, Commane R et al. Annu Rev Environ Resour 2022; 47: 343–71. 10.1146/annurev-environ-012220-011847

[5] Masson-Delmotte V, Zhai P, Pirani A et al. IPCC, *Climate Change 2021: the Physical Science Basis* Contribution of Working Group I to the Sixth Assessment Report of the Intergovernmental Panel on Climate Change. Basel: Cambridge University Press, 2021, 583–4.

[6] Foster GL, Royer DL, Lunt DJ. Nat Commun 2017; 8: 14845. 10.1038/ncomms14845 28375201 PMC5382278

[7] Chen I-C, Hill JK, Ohlemüller R et al. Science 2011; 333: 1024–6. 10.1126/science.1206432 21852500

[8] Scheffers BR, De Meester L, Bridge TCL et al. Science 2016; 354: aaf7671. 10.1126/science.aaf7671 27846577

[9] Elven R, Murray DF, Razzhivin VY et al. Annotated Checklist of the Panarctic Flora (PAF): Vascular plants. https://panarcticflora.org/pages/background.html (1 February 2022, date last accessed).

[10] Stubbs RL, Soltis DE, Cellinese N. Ecol Evol 2018; 8: 7164–77. 10.1002/ece3.4242 30073075 PMC6065370

[11] Oke TA, Stralberg D, Reid DG et al. Divers Distrib 2023; 29: 509–23. 10.1111/ddi.13674

[12] Niskanen AKJ, Niittynen P, Aalto J et al. Divers Distrib 2019; 25: 809–21. 10.1111/ddi.12889

[13] Walker DA, Raynolds MK, Daniëls FJA et al. J Veg Sci 2005; 16: 267–82. 10.1111/j.1654-1103.2005.tb02365.x

[14] Myers-Smith IH, Kerby JT, Phoenix GK et al. Nat Clim Chang 2020; 10: 106–17. 10.1038/s41558-019-0688-1

[15] Storch D, Keil P, Jetz W. Nature 2012; 488: 78–81. 10.1038/nature11226 22722856

[16] Araújo MB, Anderson RP, Márcia Barbosa A et al. Sci Adv 2019; 5: eaat4858. 10.1126/sciadv.aat4858 30746437 PMC6357756

[17] Feng X, Peterson AT, Aguirre-López LJ et al. Biol Rev 2024; 99: 1481–503. 10.1111/brv.13077 38597328

