# Supplementary material for: The future of the Arctic flora under climate change
Source: Natl Sci Rev. 2026 Feb 11;13(6):nwag096. doi: 10.1093/nsr/nwag096 (PMC13017705; doi:10.1093/nsr/nwag096)
Supplement: nwag096_Supplemental_File [file nwag096_supplemental_file.pdf]

Supplementary Information for

The future of the Arctic flora under climate change

Jun Zhang, Huan-Wen Peng, Hai-Tao Ding, Guoke Chen, Andrey S. Erst, Jin-Feng Li, Lian Lian, Fu-Cai Xia, Xiaoqi Zhou*, Robert A. Spicer*, Jian Yang* and Wei Wang*

*** Corresponding authors.** emails: wangwei1127@ibcas.ac.cn; yangjian@ibcas.ac.cn; xqzhou@des.ecnu.edu.cn; r.a.spicer@open.ac.uk

The PDF file includes:

Supplementary Materials and Methods

Figs. S1 to S41

Tables S1 to S10

References

Other Supplementary Materials for this manuscript include the following:

Data S1 to S3

Supplementary Materials and Methods

Study area

From an ecological perspective it is more meaningful in this study to use tree lines of the boreal forest as the southern boundary of the Arctic [18]. In terms of ecological borders, the Arctic land area comprises about 7.02 million km^2, which is approximately 4.8% of Earth's land surface [19] (Fig. 1a). The Arctic has a relatively consistent core of plant species that have a circumpolar distribution, but in the regional floras there is considerable variation from east to west due to many factors (including different histories related to glaciations, land bridges, and north-south trending mountain ranges) [13]. To analyze variations in species distributions in the Arctic, based on Walker et al. [13], we divided the Arctic into five sectors (European Russia-West Siberia: ER-WSS, East Siberia: ESS, Beringia: BS, Canada: CS, and North Atlantic: NAS; Fig. 1a) based on the distribution boundaries of vascular plant species. The Arctic flora land area is $\sim 7.07 \times 10^6$ km^2 based on a spatial resolution of 2.5 arc minutes, which is less than 2% different from the total area circumscribed in the previous 1-km resolution (7.02×10^6 km^2) [19]. The land area of the five sectors is $\sim 5.81 \times 10^5$ km^2 (ER-WSS), $\sim 8.65 \times 10^5$ km^2 (ESS), $\sim 9.07 \times 10^5$ km^2 (BS), $\sim 2.09 \times 10^6$ km^2 (CS), and $\sim 2.62 \times 10^5$ km^2 (NAS, Fig. 1a).

Species occurrence data

Species occurrence data, downloaded from the Global Biodiversity Information Facility (GBIF; http://www.gbif.org/; accessed February 2021), yield a total of 12,610,740 occurrence records for 2,041 species based on the *Checklist of the Panarctic flora (PAF) Vascular Plants* (http://panarcticflora.org/). All records are filtered to include only georeferenced occurrences since the 1990 with higher geolocation accuracy. We then retain the occurrence records of Arctic

plants at high latitudes (>50° N; 11,876,396) of Eurasia and North America and remove records in areas of ocean and glaciers. In addition, duplicate occurrences within a 5 km range are removed to reduce spatial autocorrelation through spatial filtering. The prediction ability of a species distribution model with occurrences of less than 25 may be poor [20]. To avoid modeling issues related to low sample size (e.g., model convergence, number of covariates) only species with a minimum of 25 remaining records were retained for the analyses. Finally, we are left with 2,306,430 occurrence records for 1,187 species (~58.2%, 1,187/2,041) belonging to 32 orders, 86 families, and 324 genera for the subsequent prediction. They represent ~94.5% of the Arctic vascular plant families (86/91) and ~76.2% of all Arctic vascular plant genera (324/425, Fig. S2; Data S1).

Environmental variables

Nineteen bioclimatic variables for the present and future are downloaded from the WorldClim database (version 2.1, http:// www.worldclim.org/) with a resolution of 2.5 arc minutes. Recently, the new generation of scenarios (Shared Socioeconomic Pathways; SSPs) has a much more nuanced approach to baselines, and the climate impacts we are likely to experience can be communicated more clearly [21]. SSPs of the Coupled Model Intercomparison Project (CMIP6) are considered to be more reasonable future climate scenarios than the representative concentration pathway of CMIP5 [22]. For future climate scenarios, we used the CNRM-CM6-1 climate system model of CMIP6 [23]. This approach of restricting our climate constraints to output from a single model, but one yielding typical ensemble outcomes, ensures coherent physicality, which may not exist if we used ensemble means. To present a variety of diverse climate change outcomes by the end of the century, SSPs provide a set of scenarios. They

include scenarios with high and very high GHG emissions (SSP3-7.0 and SSP5-8.5) and CO_2 emissions that roughly double from current levels by 2100 and 2050, respectively, scenarios with intermediate GHG emissions (SSP2-4.5) and CO_2 emissions remaining around current levels until the middle of the century, and scenarios with very low and low GHG emissions and CO_2 emissions declining to net zero around or after 2050, followed by varying levels of net negative CO_2 emissions (SSP1-1.9 and SSP1-2.6) (IPCC, 2021). Here we examine Arctic plant responses under the range of possible emission scenarios modelled in the CMIP6 model. We obtained the bioclimatic variables for four emission scenarios: SSP1-2.6, SSP2-4.5, SSP3-7.0, and SSP5-8.5 for the 2030s (2021–2040), 2050s (2041–2060), 2070s (2061–2080), and 2090s (2081–2100), respectively.

To avoid overfitting the model due to multi-collinearity of environmental variables [24], a multicollinearity test is conducted to examine the extent of cross-correlation in R version 4.1.2 [25], and variables with absolute cross-correlation coefficient values of ≥.0.80| are excluded. The initial 19 predictor variable in nine (Table S10) with the different combinations is retained in the final species model. Considering computational limitations and minimising the impact of niche truncation on spatial and temporal predictions, we use an extended bounding box covering the entire Arctic region (ranging from 50°N to 90°N) to extract climate data for modelling.

Species distribution modeling

We simulate species distributions for the present and future under different climate scenarios using the “ensemble” species distribution modeling methodology in the biomod2 package [26] in R v.4.1.2 [25], which incorporates predictions from multiple modeling techniques to make better and more accurate predictions. The ensemble model combines the strengths of multiple models

and aims to improve the overall predictive performance by reducing the potential biases and uncertainties associated with individual models [26, 27]. We use two metrics to evaluate the performance of the models, including the area under the curve (AUC) and the true skill statistic [28] (TSS). The AUC values range from 0.5 to 1, with higher values indicating the better performance of the model: poor (0.5–0.6), fair (0.6–0.7), good (0.7–0.8), very good (0.8–0.9), and excellent (0.9–1.0) [28]. The TSS is a threshold-dependent measure that combines sensitivity (true positive rate) and specificity (true negative rate) to assess model performance and the TSS value above 0.6 indicates good performance [29, 30]. To create an ensemble model using a weighted mean approach, we select models with a TSS value greater than 0.6. The ensemble of just the Classification Tree Analysis (CTA), Generalized Boosted Regression Model (GBM), Generalized Linear Models (GLMs), Random Forest (RF), and Maximum Entropy (MaxEnt) models are used to make the final projection. All the outputs are based on the Lambert Azimuthal Equal Area projection.

Statistical analyses

A mask of the study area (Fig. 1a) is created to only include cells of the Arctic. To distinguish between suitable and unsuitable areas in the models, we chose the maximum sensitivity plus specificity (MSS) logistic threshold, which is robust for all types of data [31]. Changes in Area of Habitat (AOH) of sampled species are calculated as the difference in range size of each species relative to now. For purposes of classifying species distributional area changes, species' AOH are considered to be decreasing/increasing when the range change is >5% of the floristic sector area. Mean Area of Habitat (MAOH) is calculated as the mean range size of all sampled species in the Arctic and the five sectors.

$\Delta AOH_{pi} = AOH_{pi+1} - AOH_{pi}$

$MAOH = (AOH_1 + AOH_2 + ... + AOH_n)/n$

ΔAOH indicates changes in area of habitat, AOH indicates area of habitat, $pi+1$ indicates the future period (2030s 2050s, 2070s or 2090s), pi indicates the current, $MAOH$ indicates mean area of habitat, n indicates species number.

We overlay distribution consensus maps for all sampled species onto a single map for each period to generate new maps representing the species richness of the focal area [32]. Then, we use these maps to extract the mean values for species richness of each region to compare trends in species richness change between the five sectors from now to 2100. The mean species richness value for each region is calculated from the mean value of species number for all grid cells within that region [32]. We measure the potential species richness changes for the Arctic and its five sectors. The richness variation value of the Arctic is obtained to generate the maps representing the richness change distributions of all sampled species under future climate scenarios compared with the current richness distribution. Then, we calculate the number of grid cells with the varying species numbers, and plot the frequency distribution histogram of species richness changes in each floristic sector under four emission scenarios.

Most Arctic species have a circumpolar distribution [33]. To better examine the migration trend of each species' range, we analyze the changes in five sectors separately. We calculated the direction and magnitude of centroid changes in the range of each species by comparing the centroids of current and future distribution maps. This distributional centroid is reduced for each species' distribution to a single central point (a centroid) to create a vector file for depicting the direction and magnitude of distribution changes over time. Moreover, we calculate the mean distributional centroid (MDC) from the distributional centroid of all sampled species in each

period. The change of centroid coordinates, the direction and magnitude of distributional shifts, reveal the general future distribution trends of all sampled species.

$MDC_{pi} = (DC1_{pi} + DC2_{pi} + ... + DCn_{pi})/n$

MDC indicates mean distributional centroid, *DC* indicates the distributional centroid of species, *pi* indicates time, *n* indicates species number.

Fig. S1.

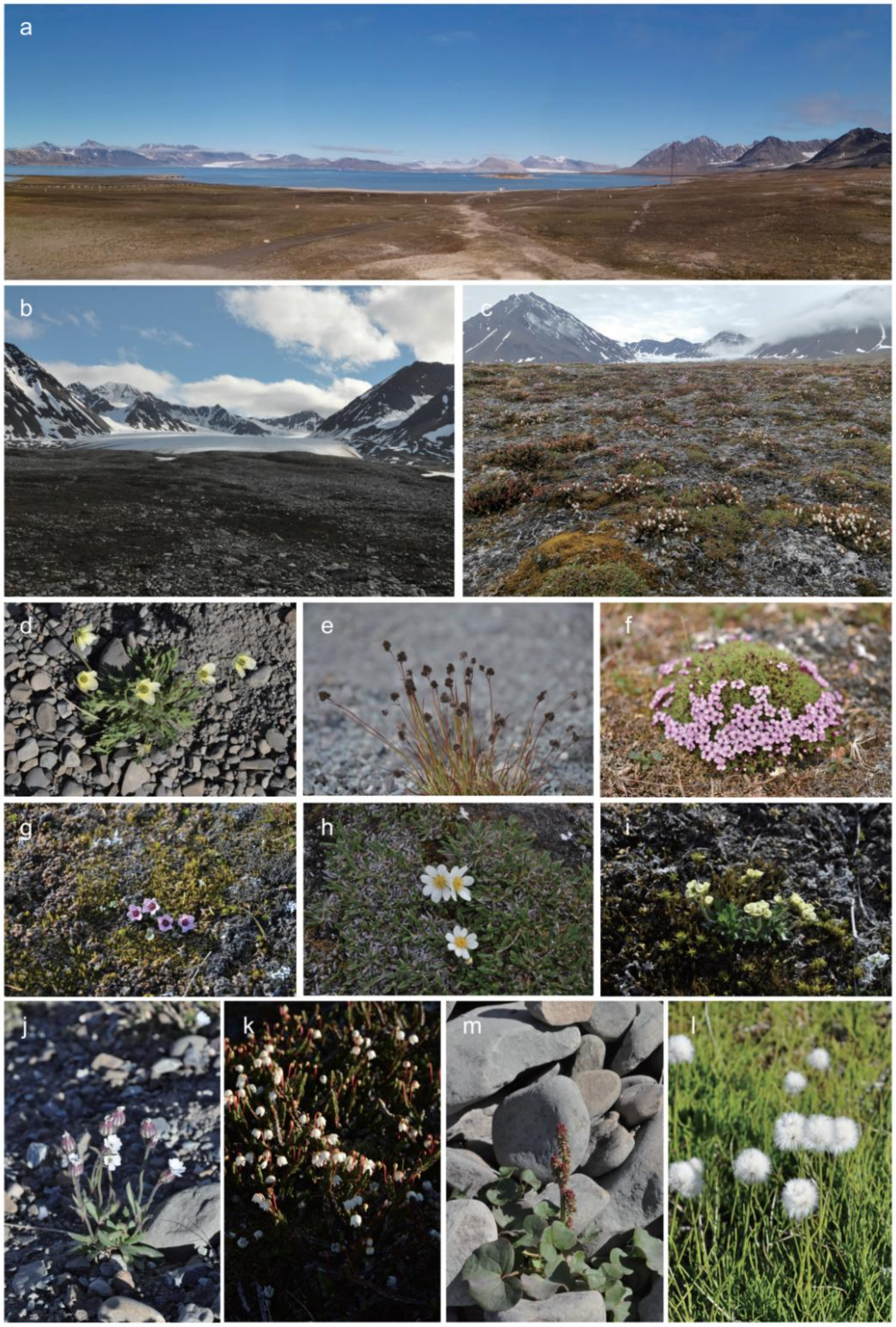

Fig. S1. Landscape (a-c) and some vascular plants (d-i) of the Svalbard archipelago in the Arctic. (a-c) The landscape around the Arctic Yellow River Station (78°55′21″N, 11°56′05″E), the forefront landscape of Glacier M (78°53′53″N, 12°04′07″E), and the forefront landscape of Glacier A (78°54′01″N, 12°09′34″E) in Ny-Ålesund of Svalbard Archipelago, photographed on

24 July 2024. (d-i) *Papaver radicatum*, *Luzula confuse*, *Silene acaulis*, *Saxifraga oppositifolia*, *Dryas octopetala*, *Draba oxycarpa*, *Silene involucrata* ssp. *furcata*, *Cassiope tetragona*, *Oxyria digyna*, and *Eriophorum scheuchzeri* ssp. *arcticum*.

Fig. S2.

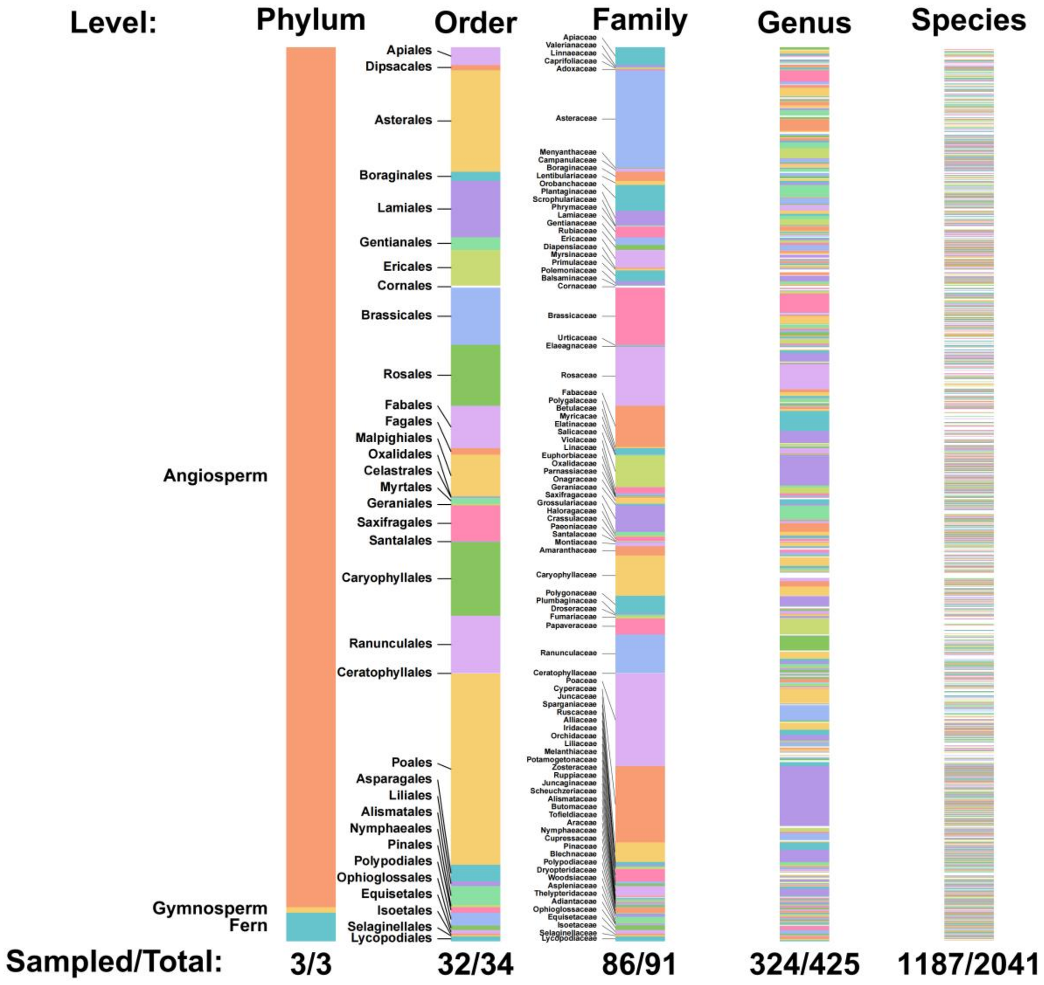

Fig. S2. Sampling of Arctic vascular plants in this study.

Fig. S3.

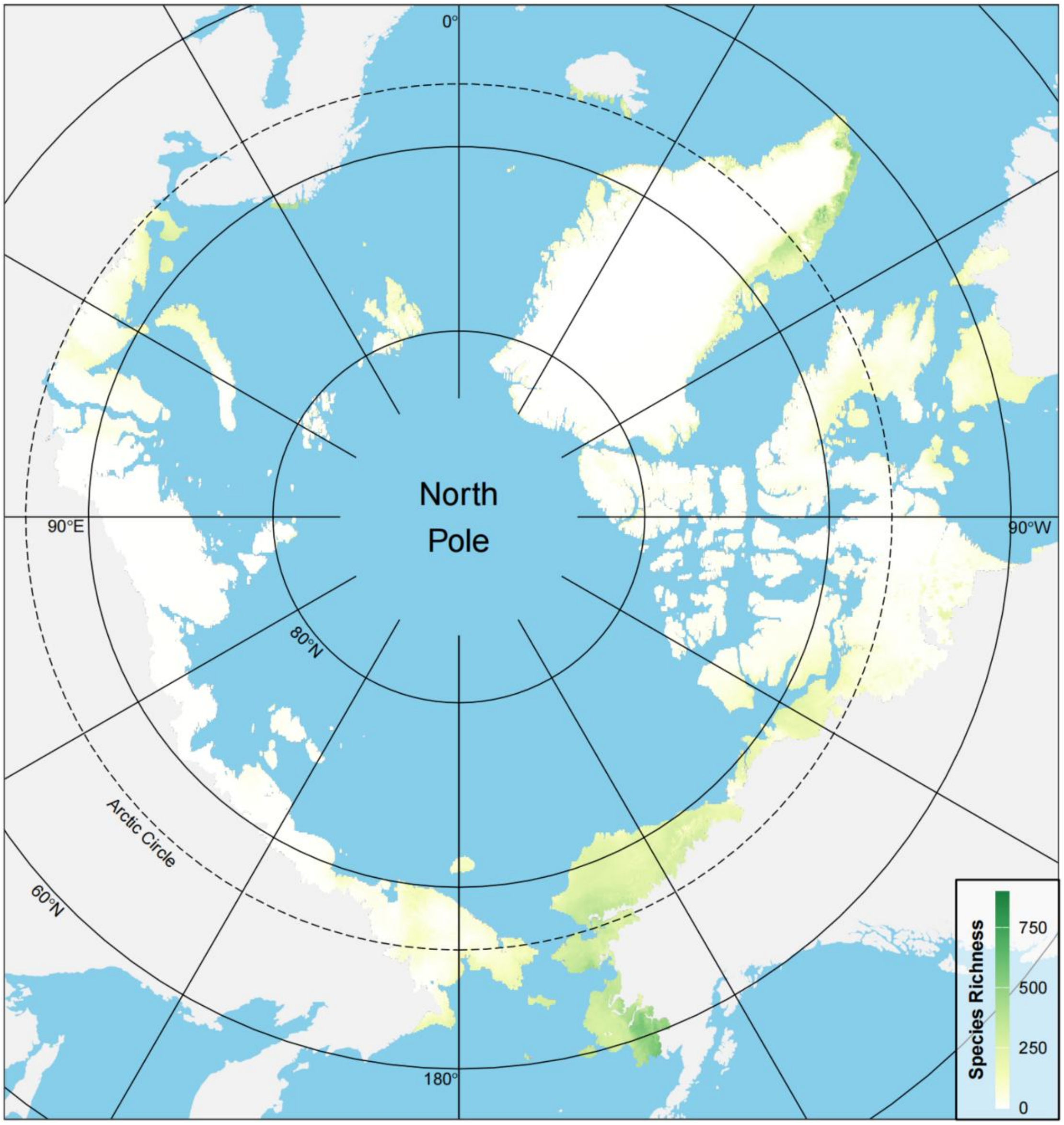

Fig. S3. The potential species richness (as represented by the sampled species) under the current climatic scenario. The colors ranging from white to green indicate species richness (the number of distinct species in each grid cell) from low to high.

Fig. S4.

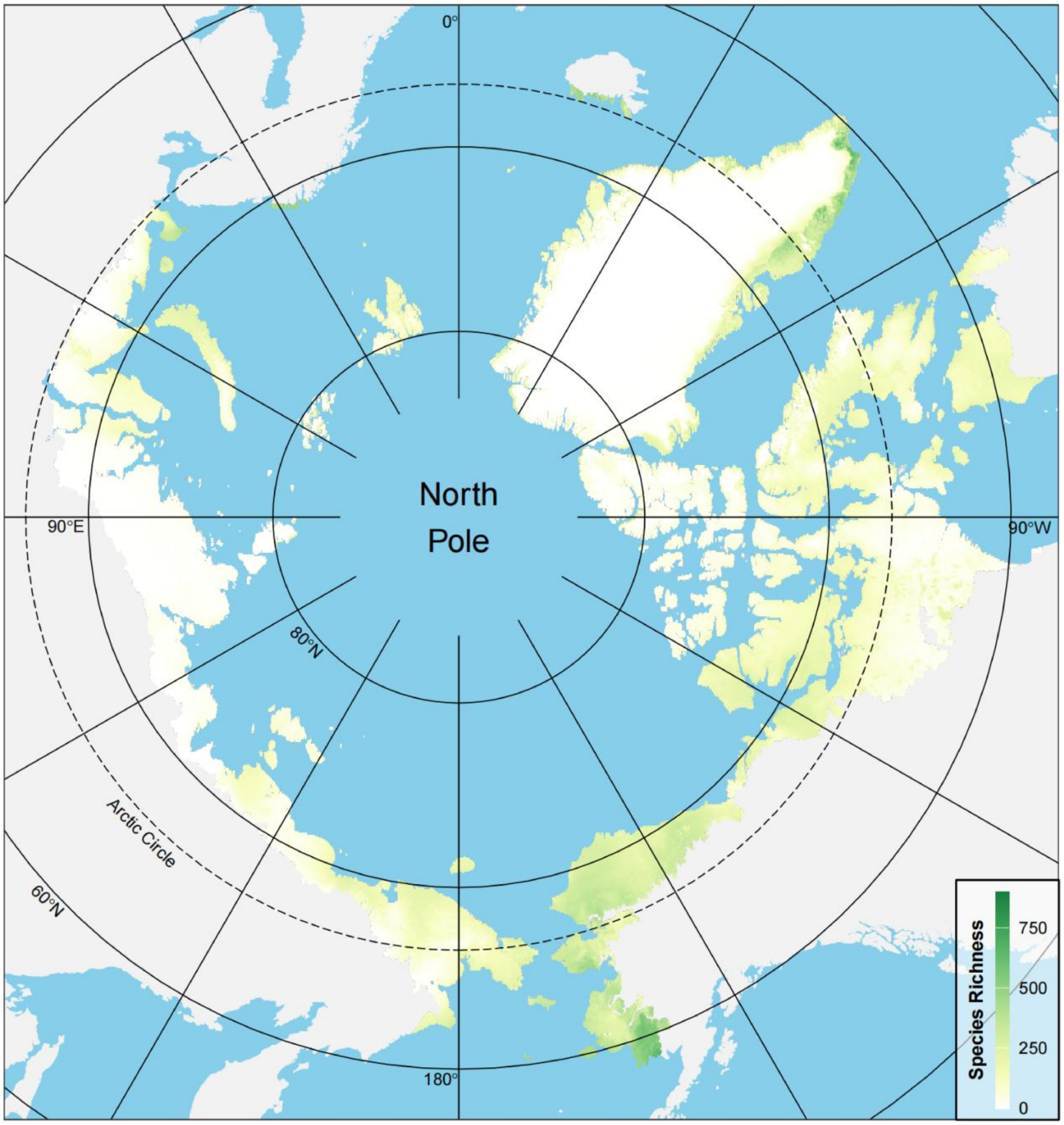

Fig. S4. The potential species richness (as represented by the sampled species) under scenario 2030s SSP1-2.6. The colors ranging from white to green indicate species richness from low to high.

Fig. S5.

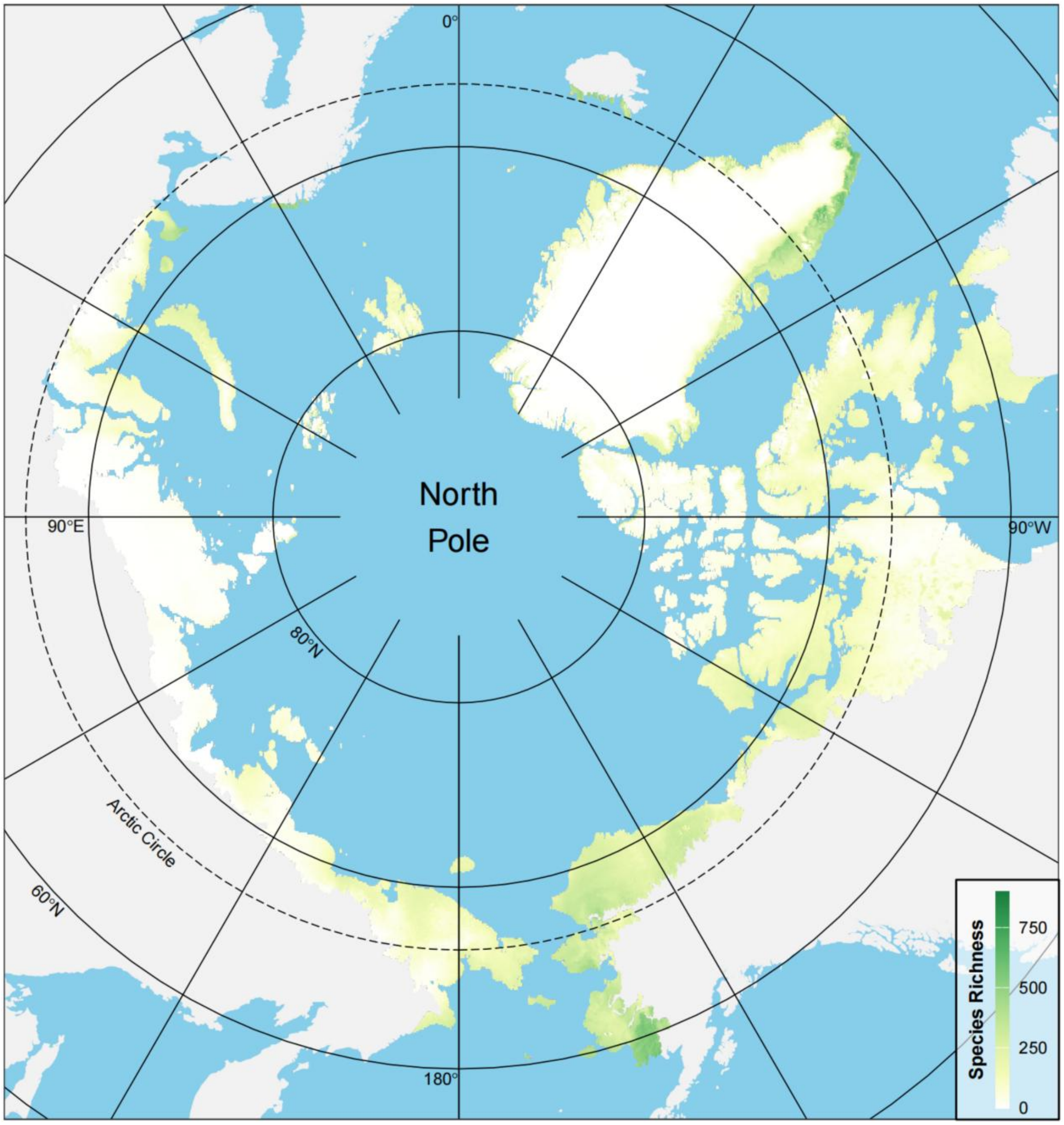

Fig. S5. The potential species richness (as represented by the sampled species) under scenario 2030s SSP2-4.5. The colors ranging from white to green indicate species richness from low to high.

Fig. S6.

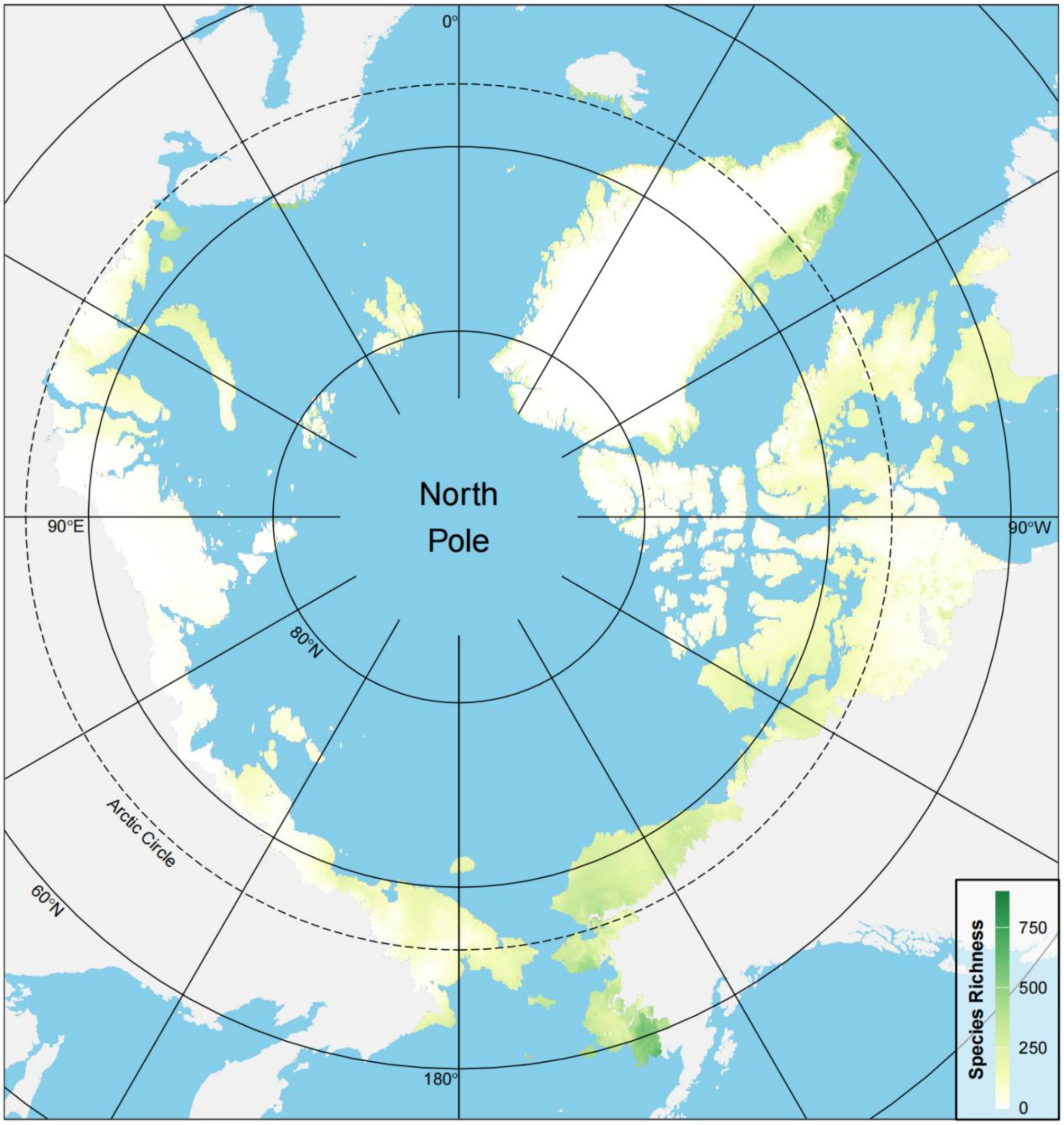

Fig. S6. The potential species richness (as represented by the sampled species) under scenario 2030s SSP3-7.0. The colors ranging from white to green indicate species richness from low to high.

Fig. S7.

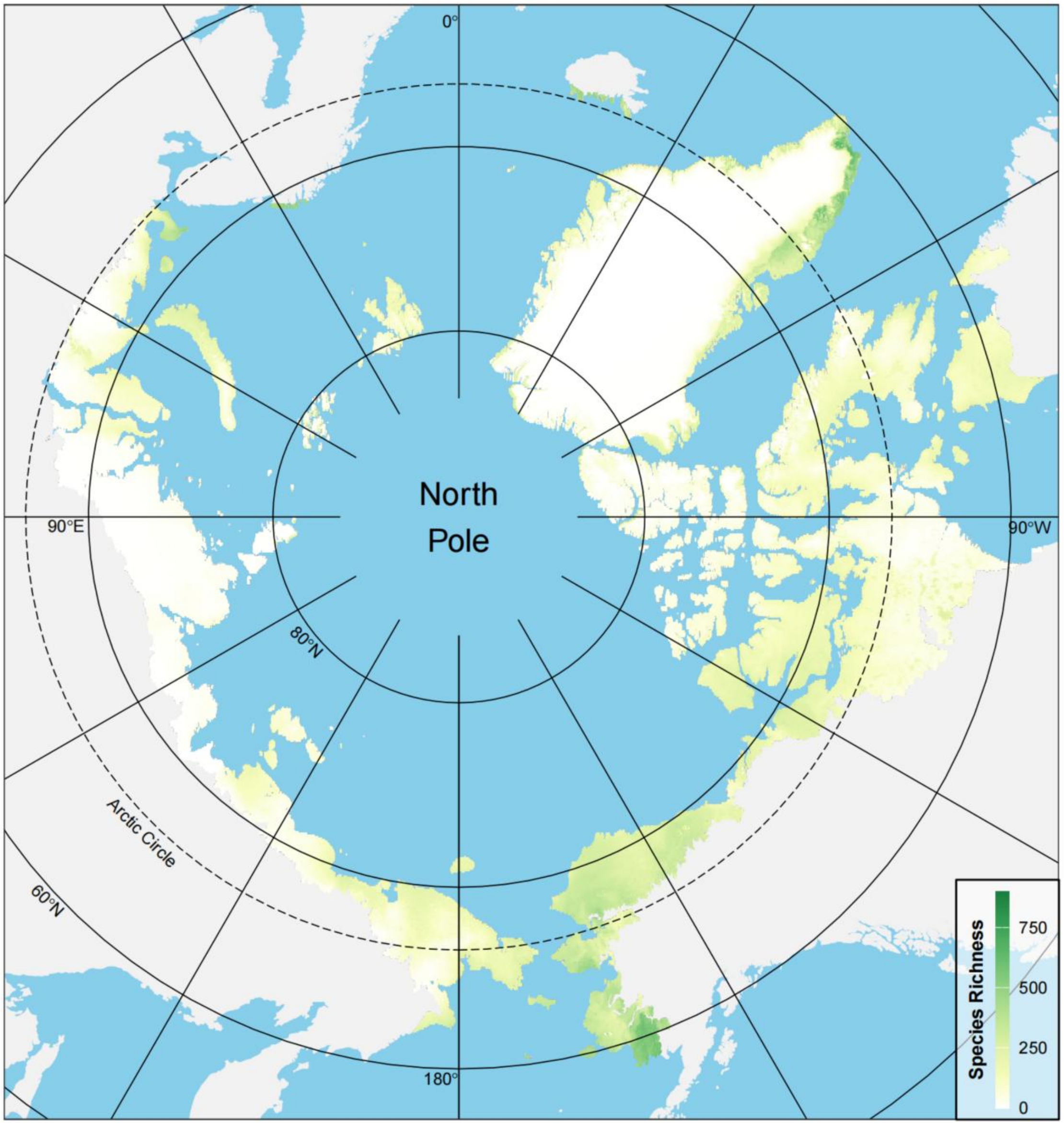

Fig. S7. The potential species richness (as represented by the sampled species) under scenario 2030s SSP5-8.5. The colors ranging from white to green indicate species richness from low to high.

Fig. S8.

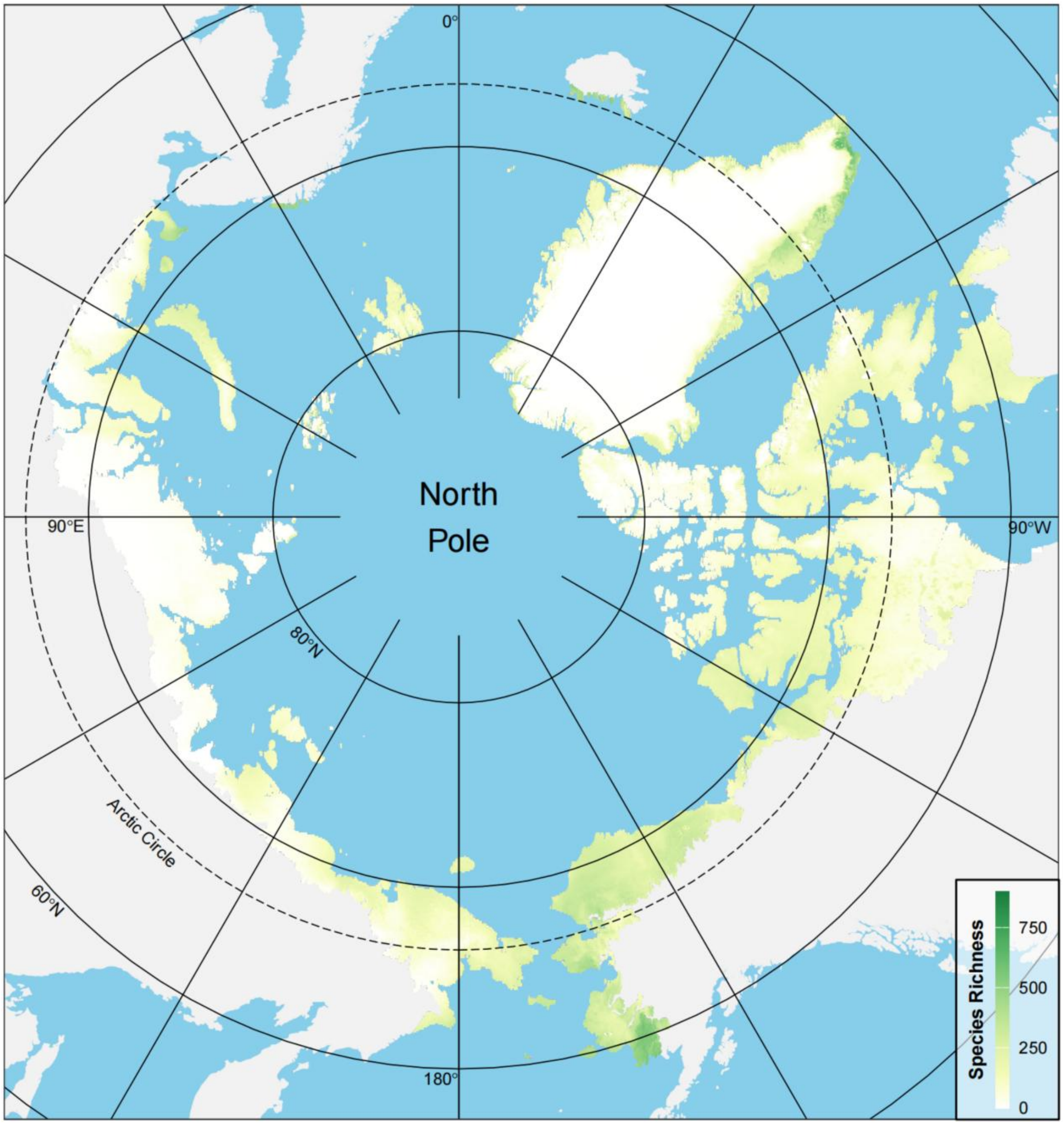

Fig. S8. The potential species richness (as represented by the sampled species) under scenario 2050s SSP1-2.6. The colors ranging from white to green indicate species richness from low to high.

Fig. S9.

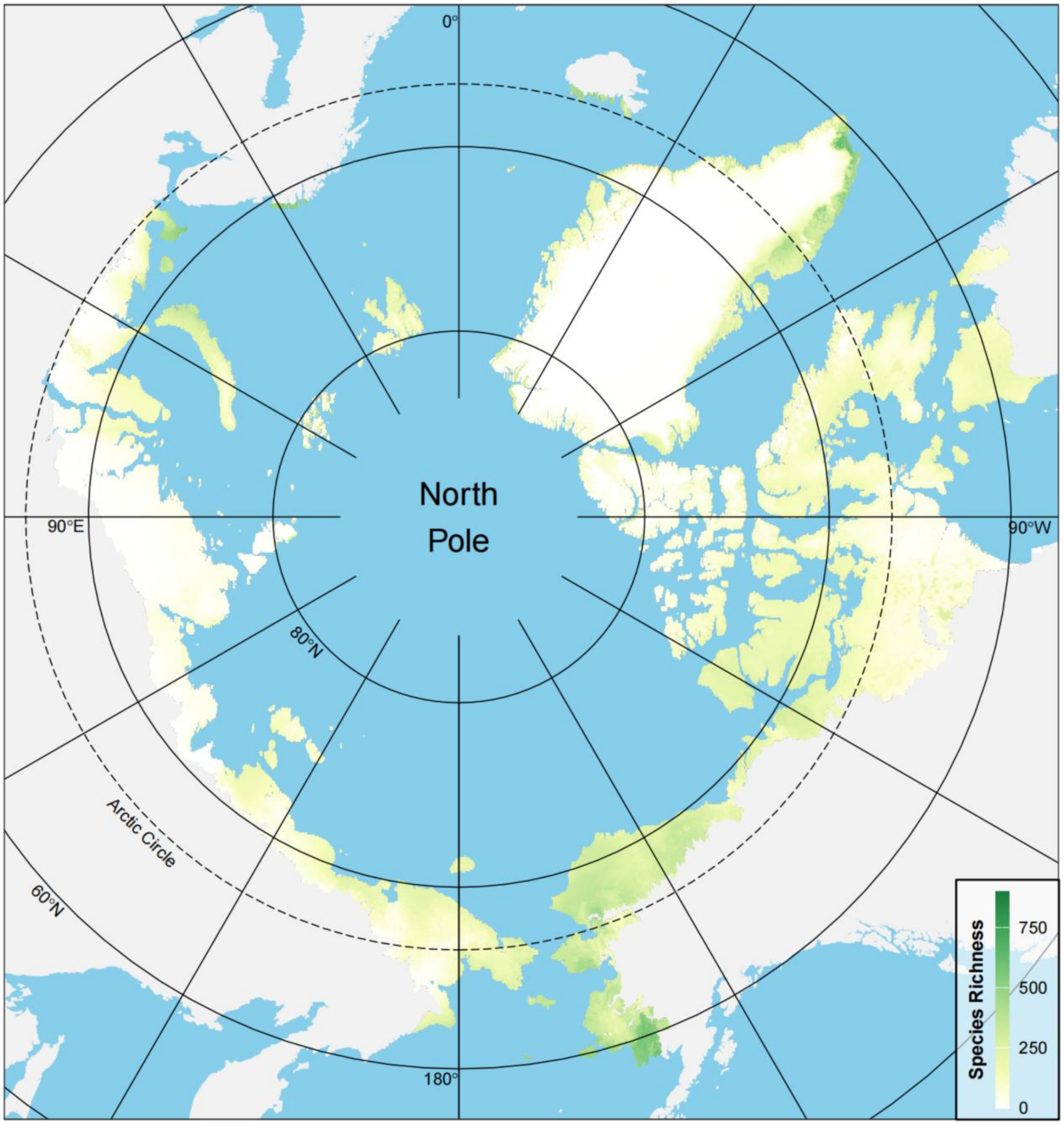

Fig. S9. The potential species richness (as represented by the sampled species) under scenario 2050s SSP2-4.5. The colors ranging from white to green indicate species richness from low to high.

Fig. S10.

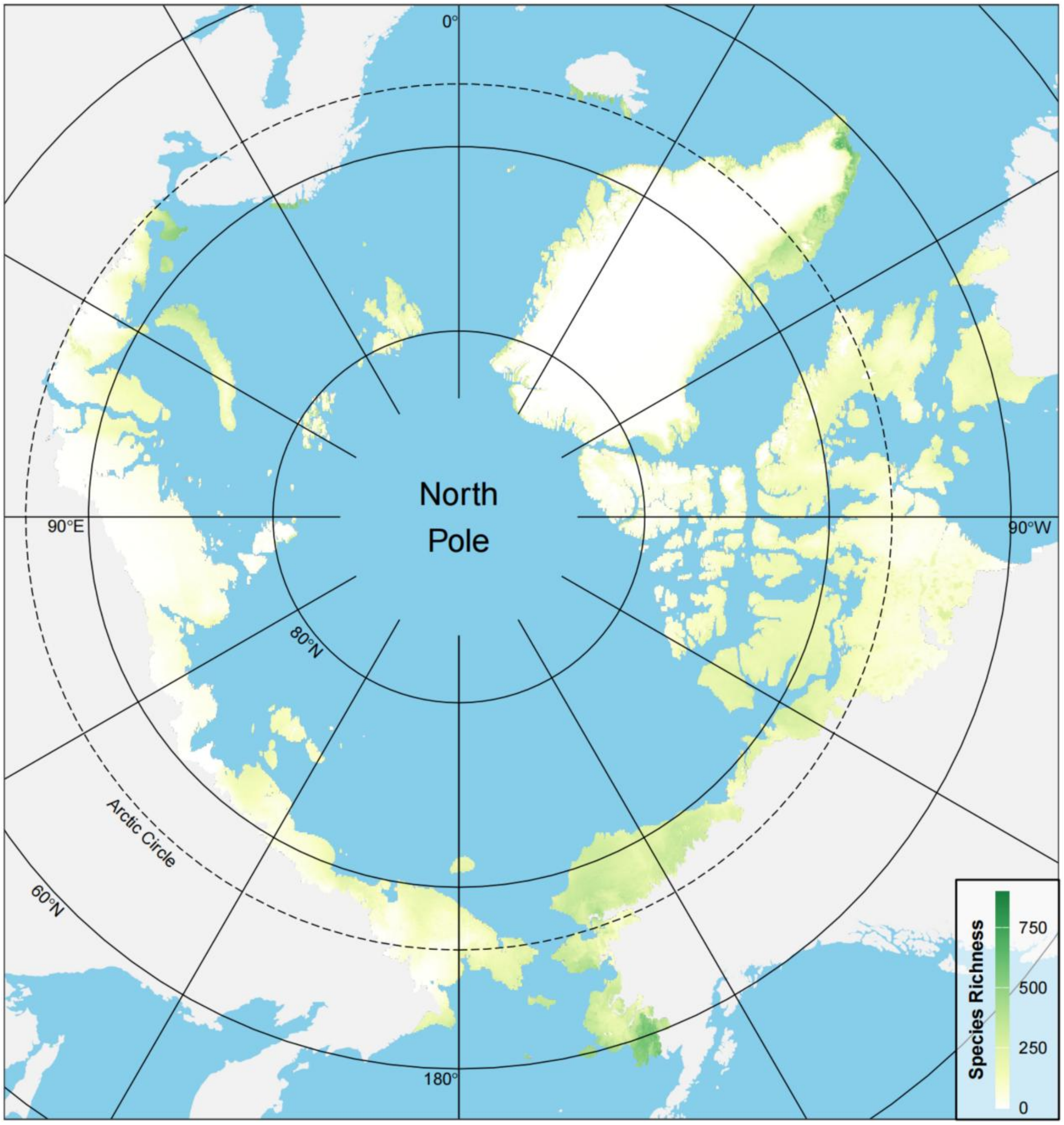

Fig. S10. The potential species richness (as represented by the sampled species) under scenario 2050s SSP3-7.0. The colors ranging from white to green indicate species richness from low to high.

Fig. S11.

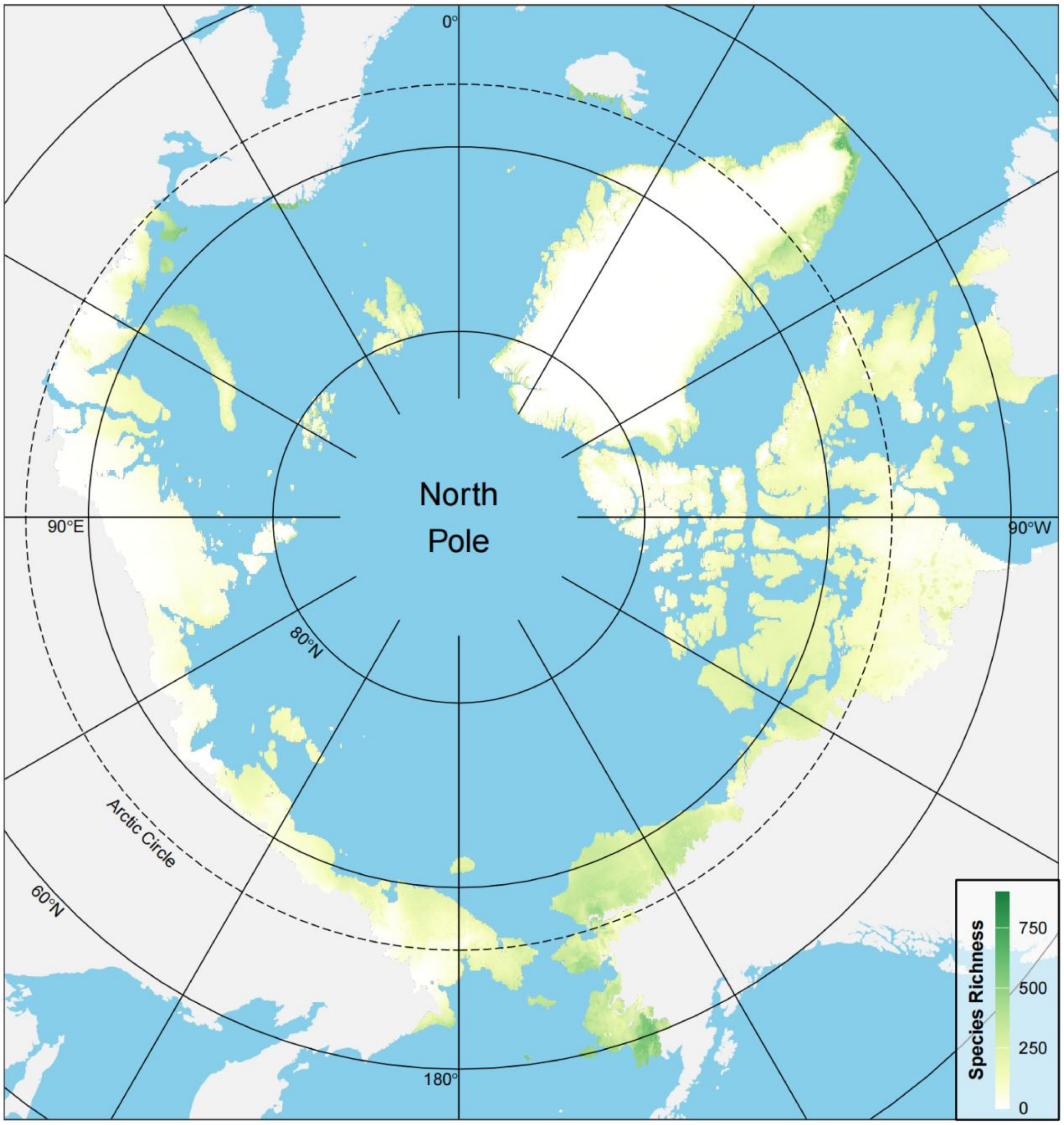

Fig. S11. The potential species richness (as represented by the sampled species) under scenario 2050s SSP5-8.5. The colors ranging from white to green indicate species richness from low to high.

Fig. S12.

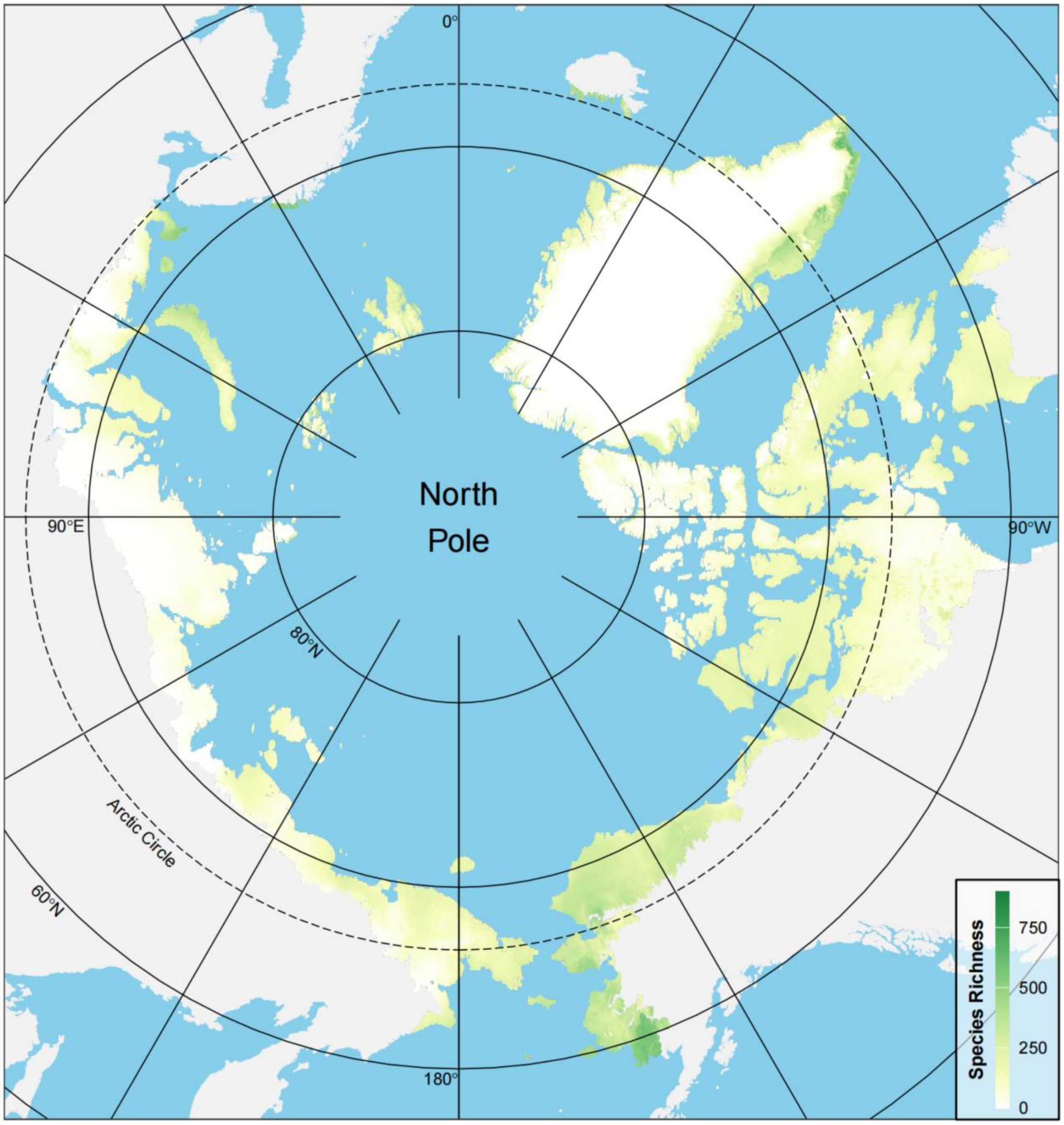

Fig. S12. The potential species richness (as represented by the sampled species) under scenario 2070s SSP1-2.6. The colors ranging from white to green indicate species richness from low to high.

Fig. S13.

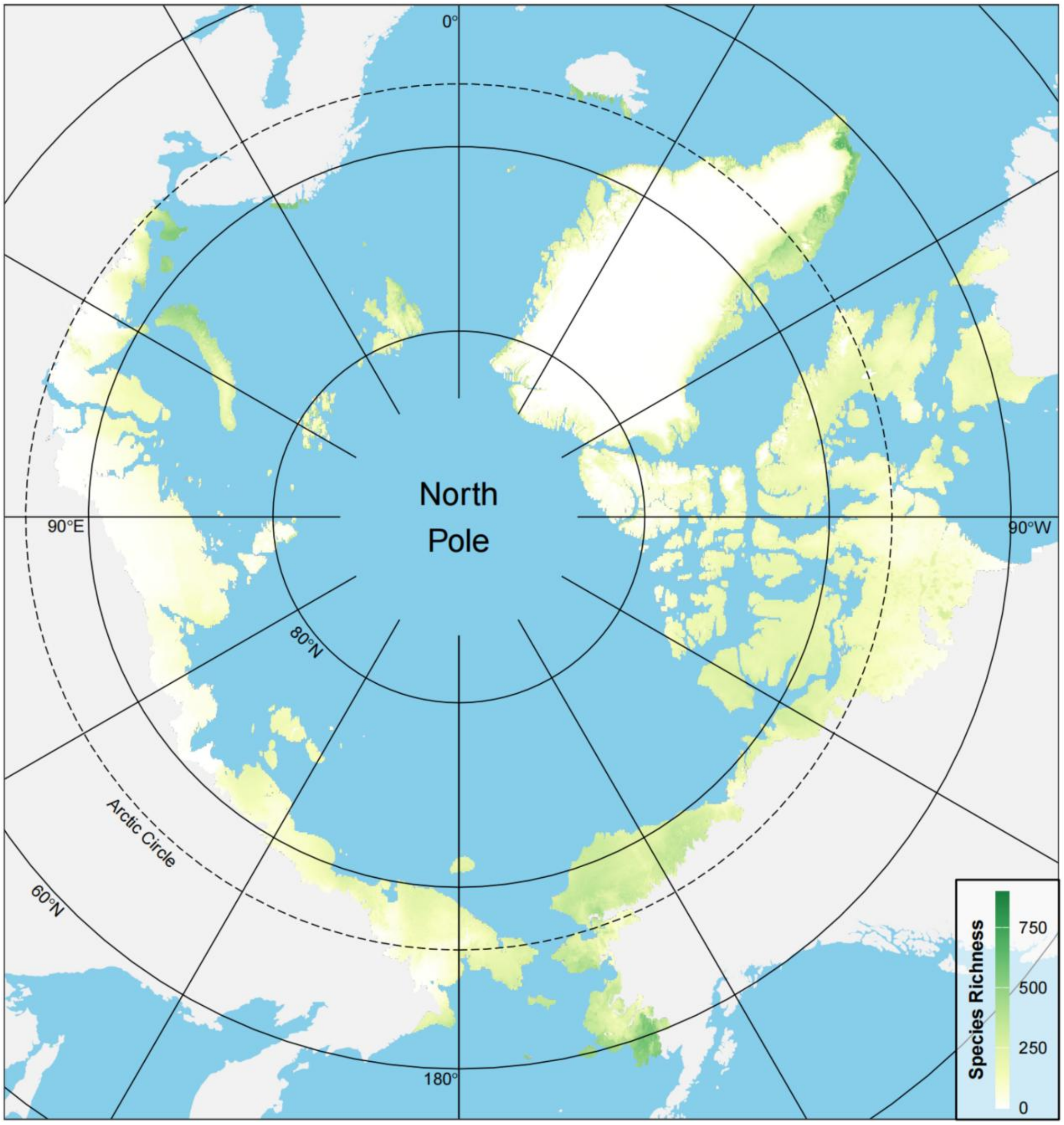

Fig. S13. The potential species richness (as represented by the sampled species) under scenario 2070s SSP2-4.5. The colors ranging from white to green indicate species richness from low to high.

Fig. S14.

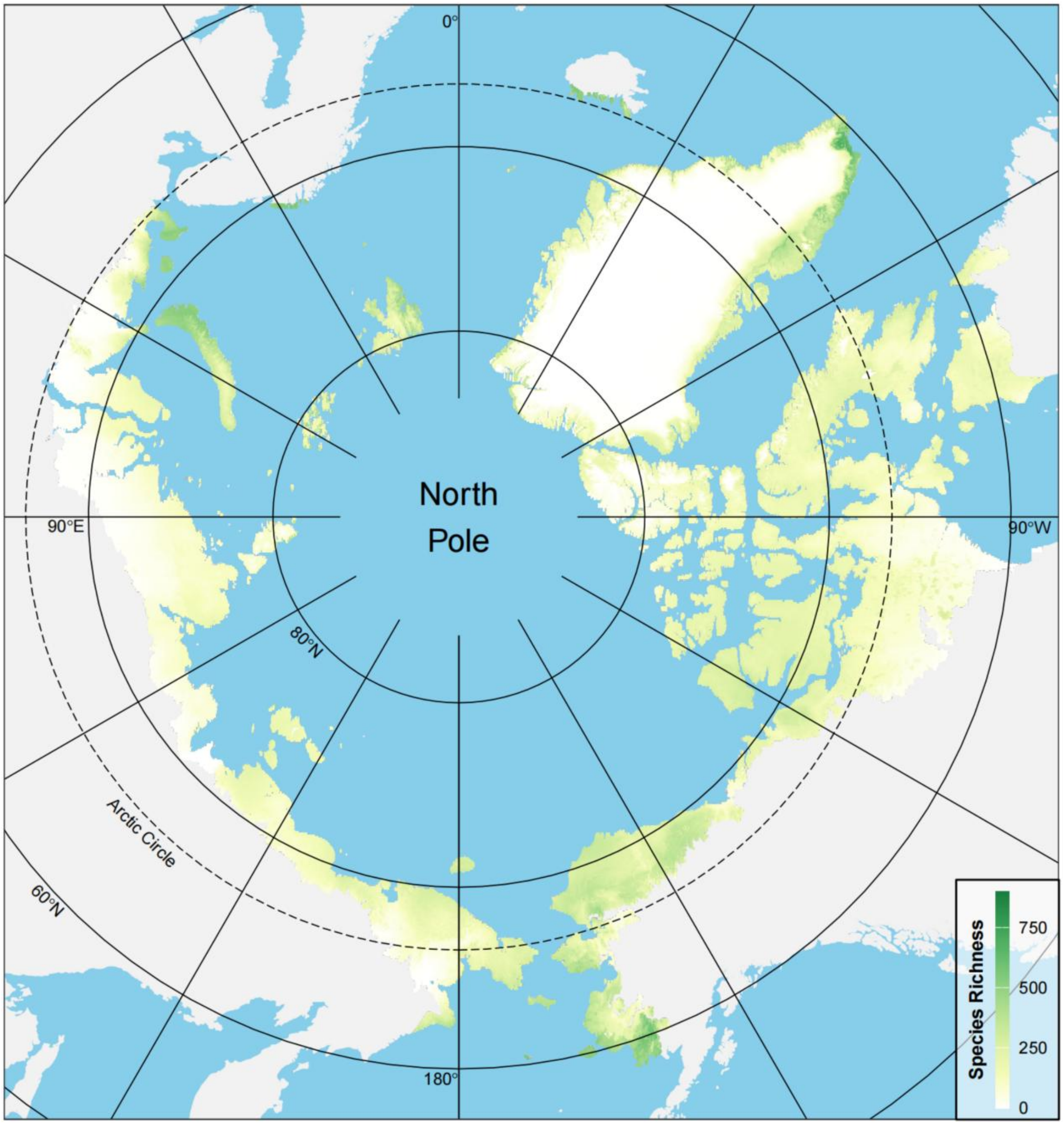

Fig. S14. The potential species richness (as represented by the sampled species) under scenario 2070s SSP3-7.0. The colors ranging from white to green indicate species richness from low to high.

Fig. S15.

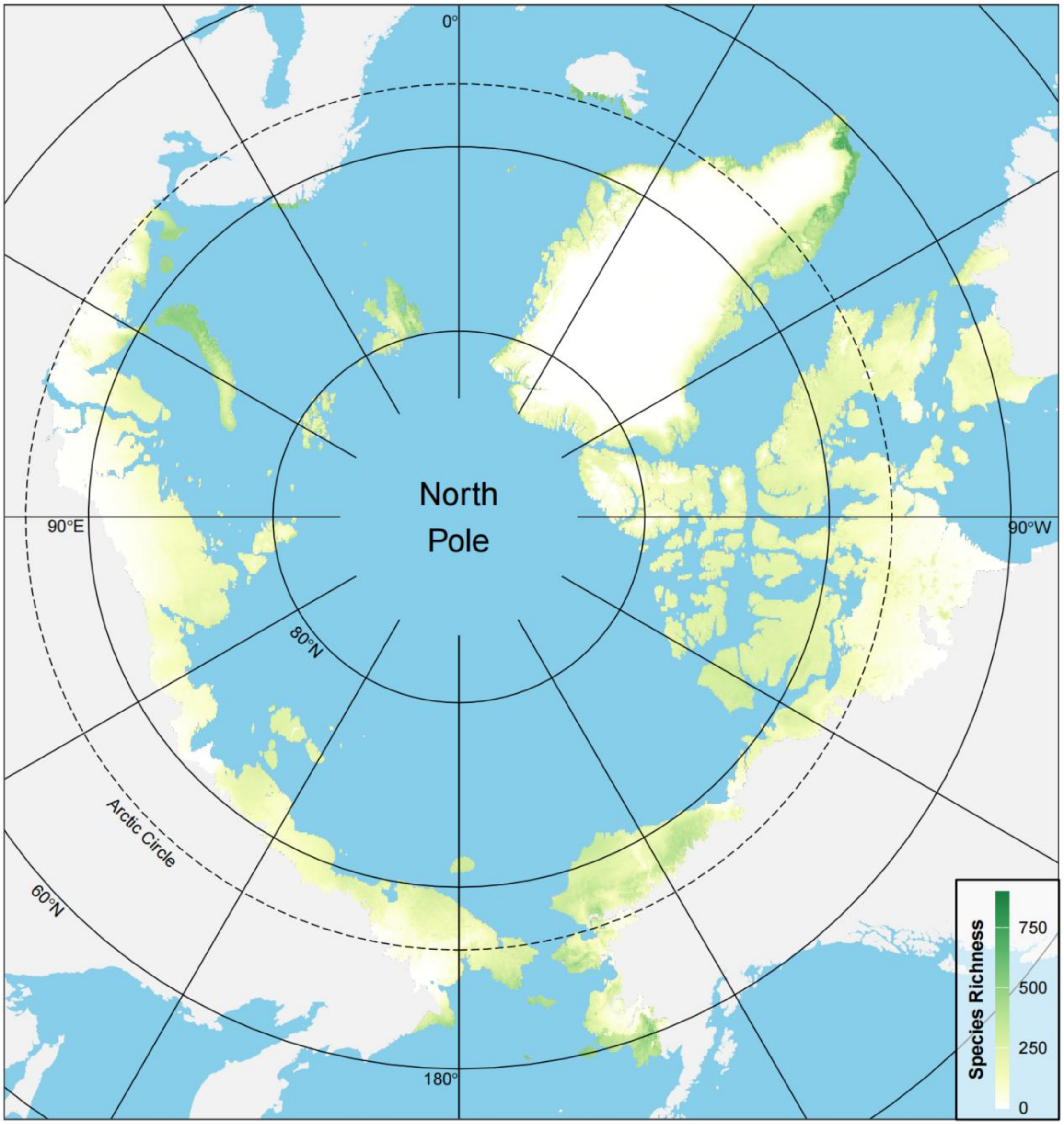

Fig. S15. The potential species richness (as represented by the sampled species) under scenario 2070s SSP5-8.5. The colors ranging from white to green indicate species richness from low to high.

Fig. S16.

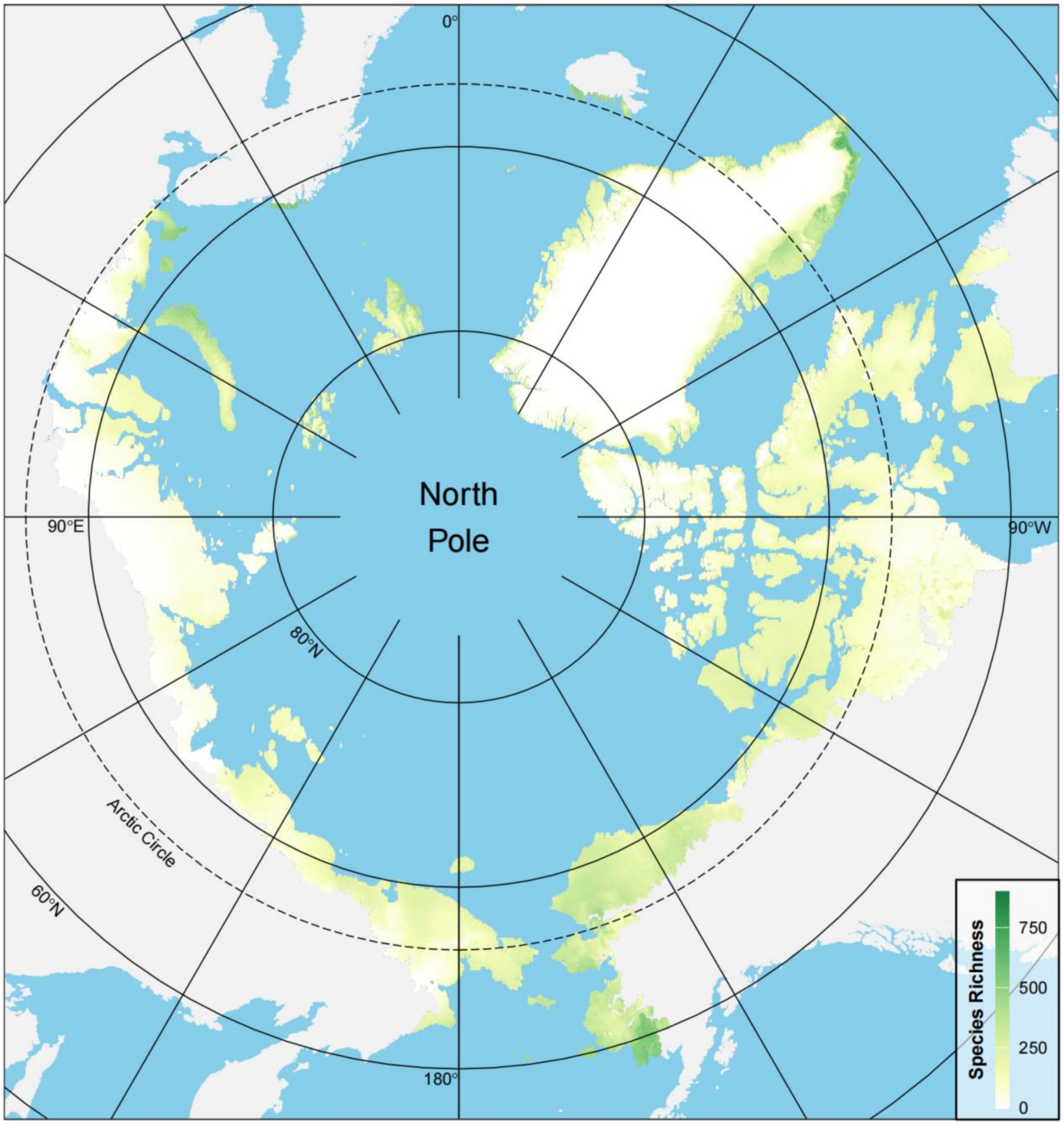

Fig. S16. The potential species richness (as represented by the sampled species) under scenario 2090s SSP1-2.6. The colors ranging from white to green indicate species richness from low to high.

Fig. S17.

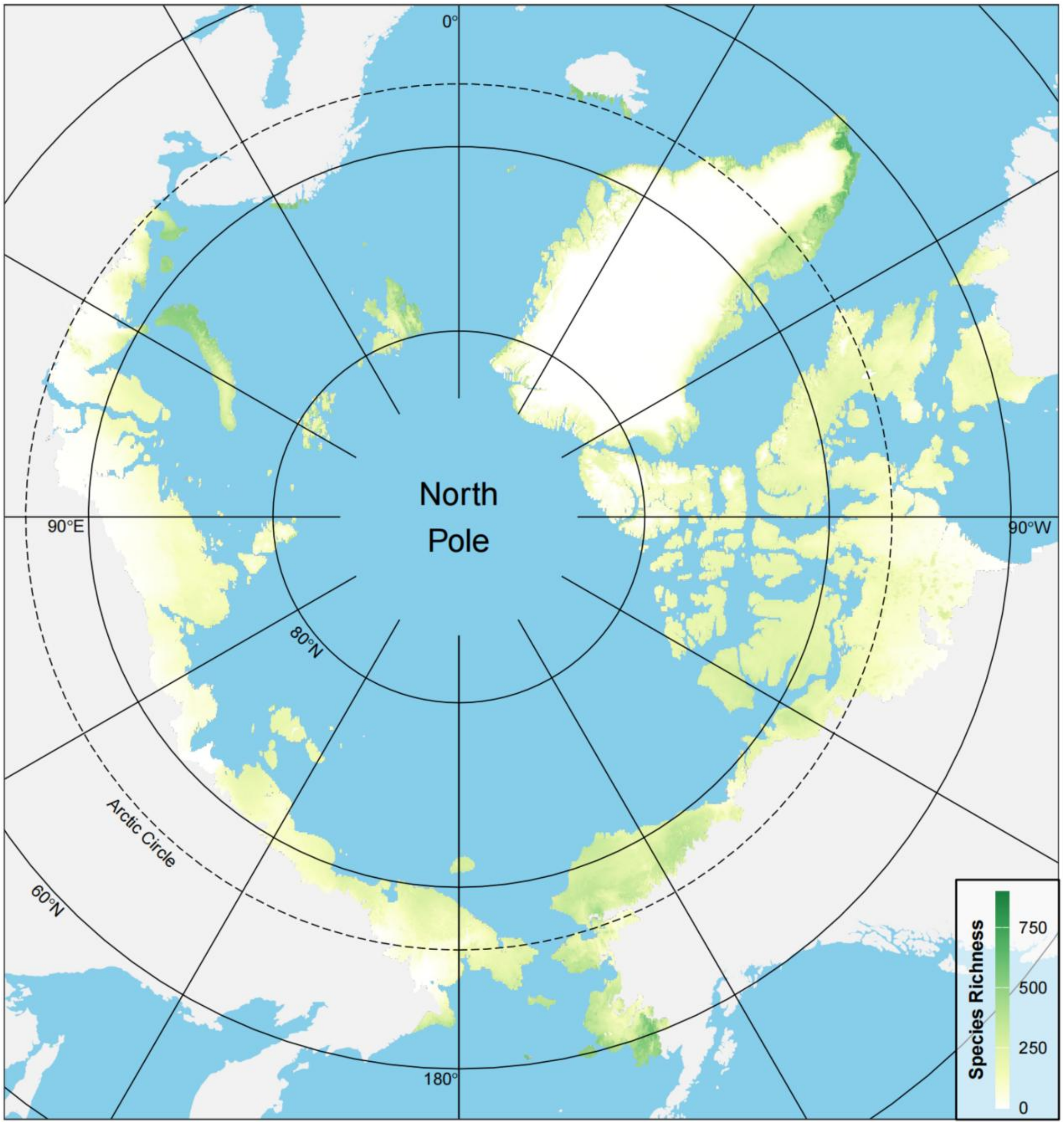

Fig. S17. The potential species richness (as represented by the sampled species) under scenario 2090s SSP2-4.5. The colors ranging from white to green indicate species richness from low to high.

Fig. S18.

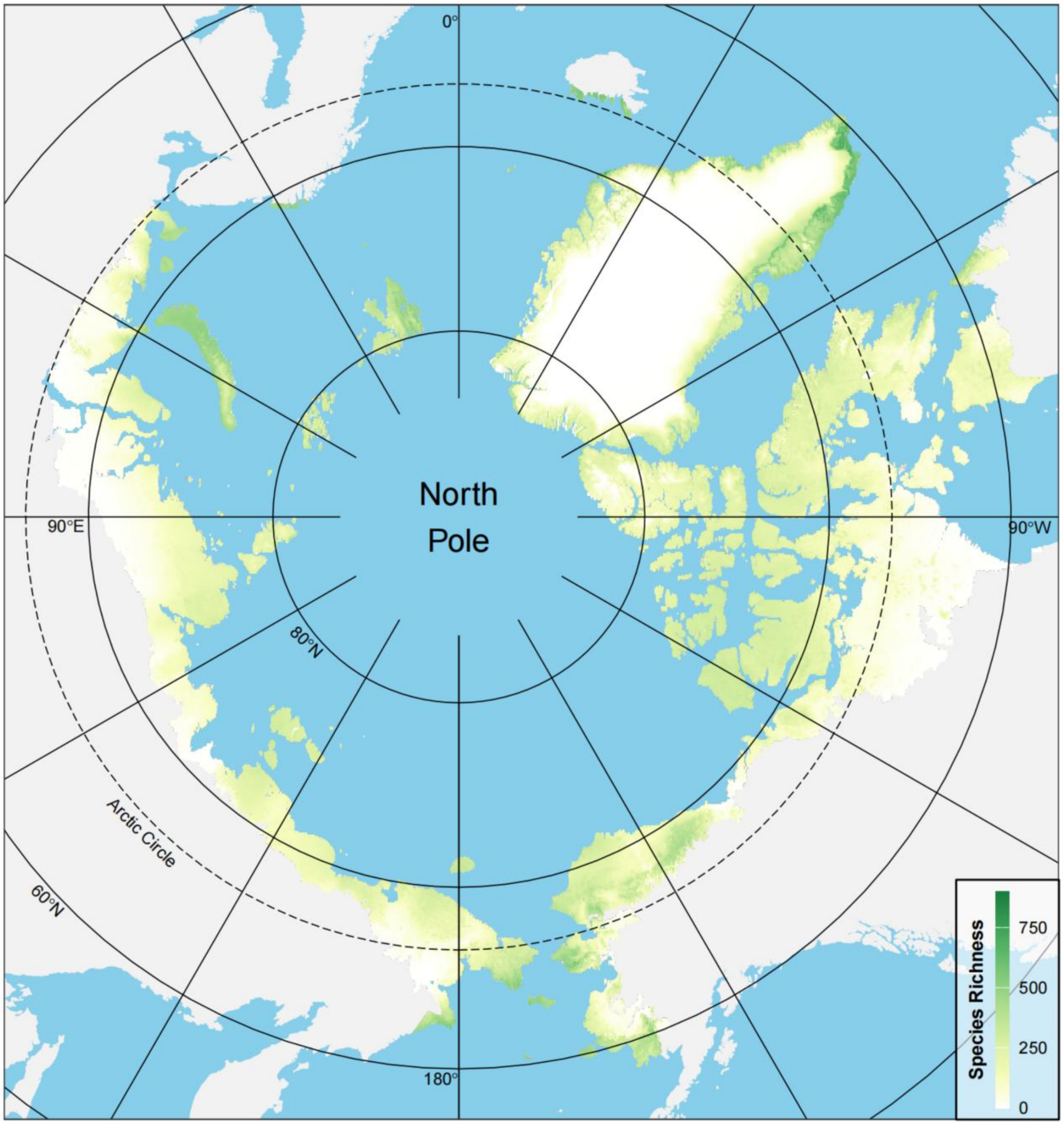

Fig. S18. The potential species richness (as represented by the sampled species) under scenario 20930s SSP3-7.0. The colors ranging from white to green indicate species richness from low to high.

Fig. S19.

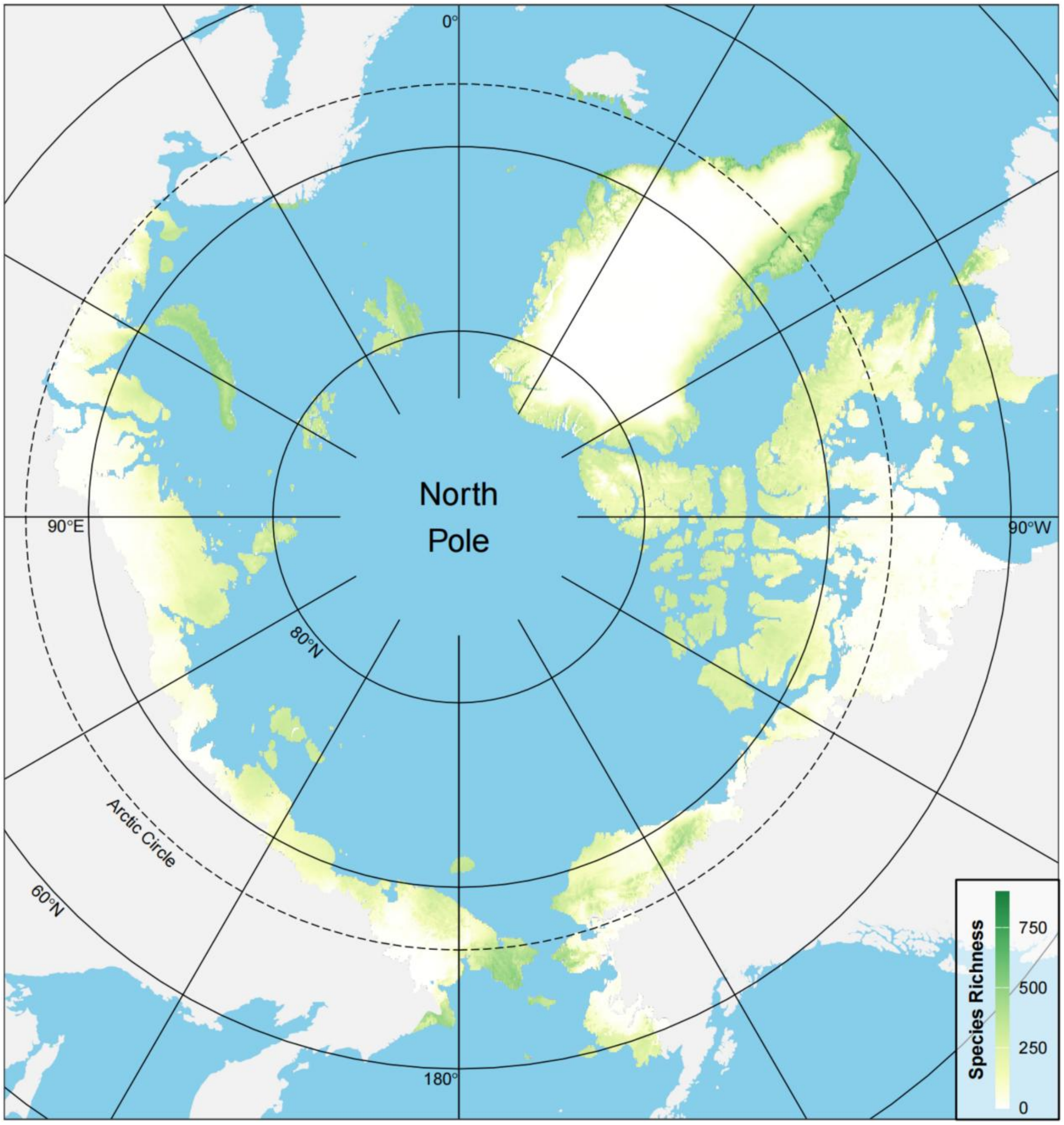

Fig. S19. The potential species richness (as represented by the sampled species) under scenario 2090s SSP5-8.5. The colors ranging from white to green indicate species richness from low to high.

Fig. S20.

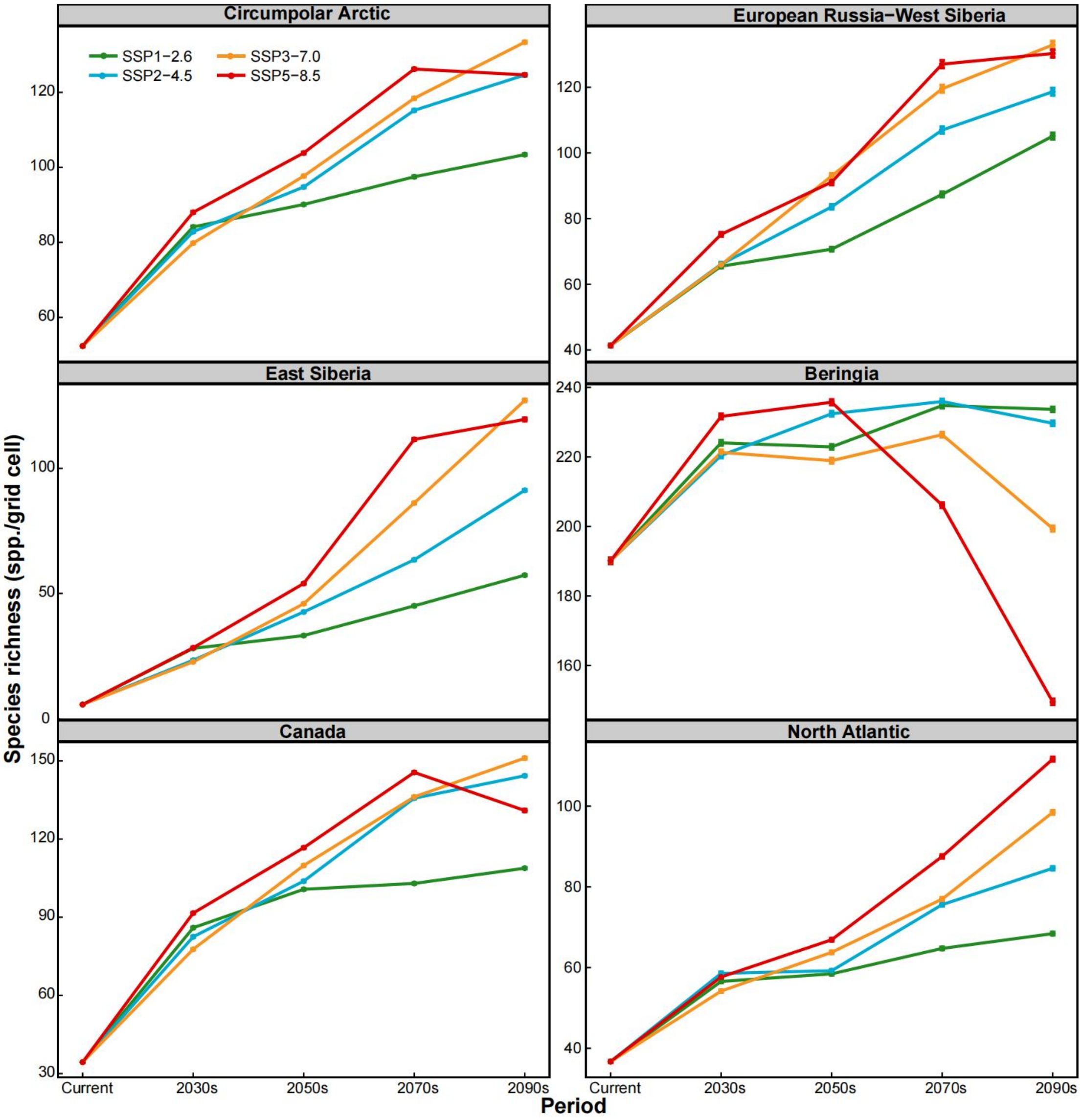

Fig. S20. The changes in mean species richness in the Arctic and five Arctic floristic sectors under four emission scenarios.

Fig. S21.

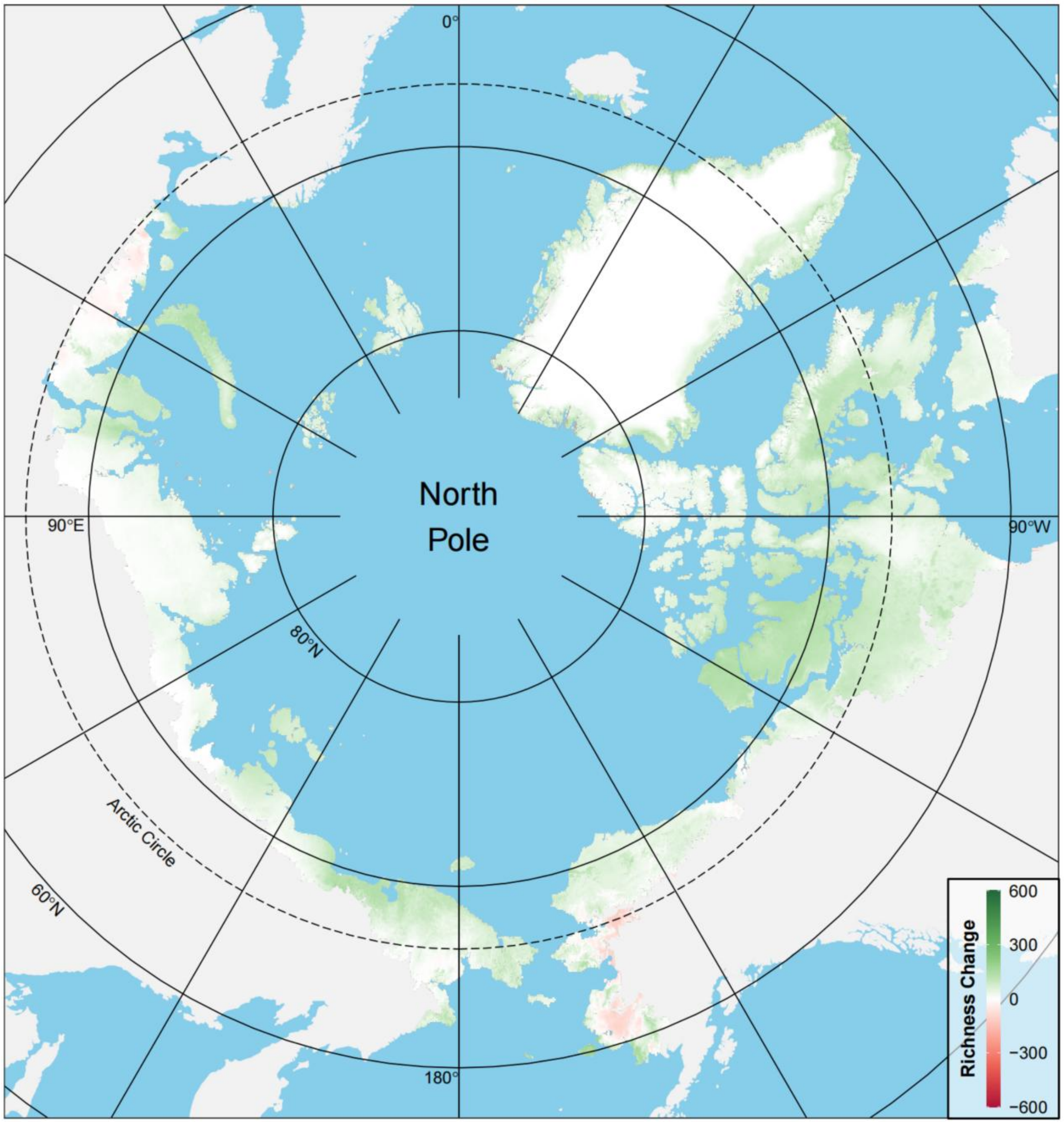

Fig. S21. Changes in the potential species richness under scenario 2030s SSP1-2.6 compared to the current climatic scenario. The colors from white to green indicate an increase in species richness, while from white to red indicate a decrease (from low to high, respectively).

Fig. S22.

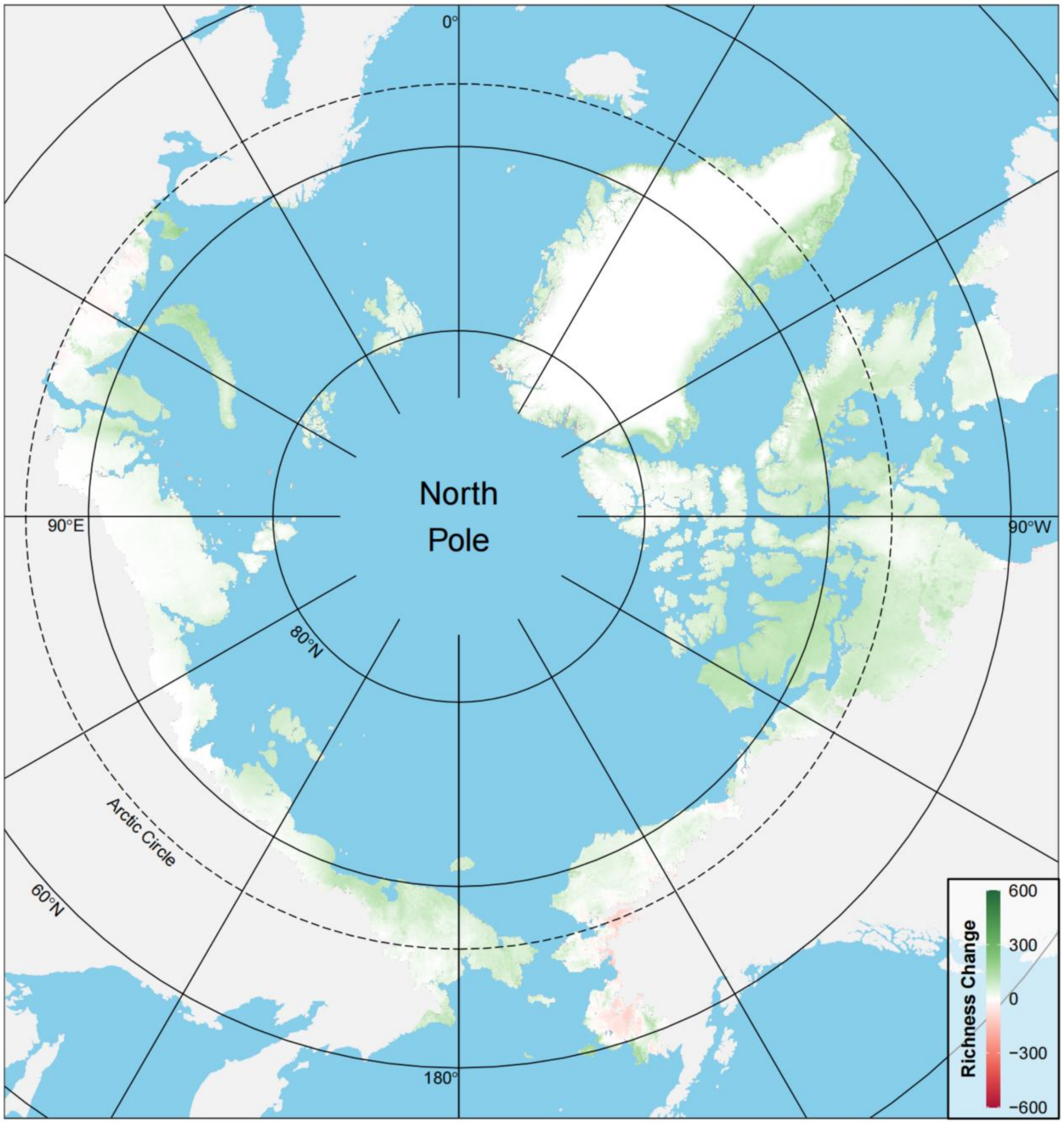

Fig. S22. Changes in the potential species richness under scenario 2030s SSP2-4.5 compared to the current climatic scenario. The colors from white to green indicate an increase in species richness, while from white to red indicate a decrease (from low to high, respectively).

Fig. S23.

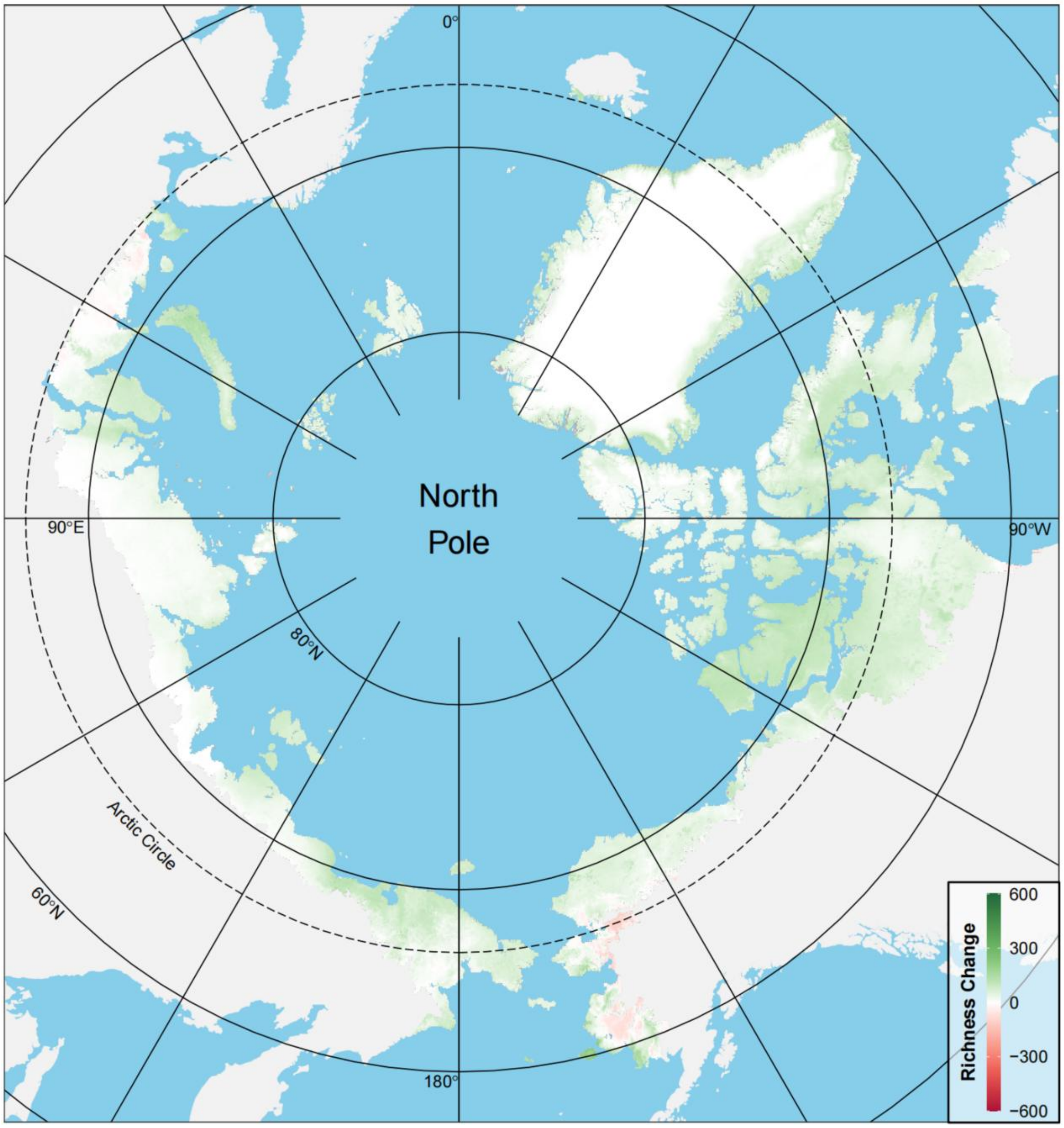

Fig. S23. Changes in the potential species richness under scenario 2030s SSP3-7.0 compared to the current climatic scenario. The colors from white to green indicate an increase in species richness, while from white to red indicate a decrease (from low to high, respectively).

Fig. S24.

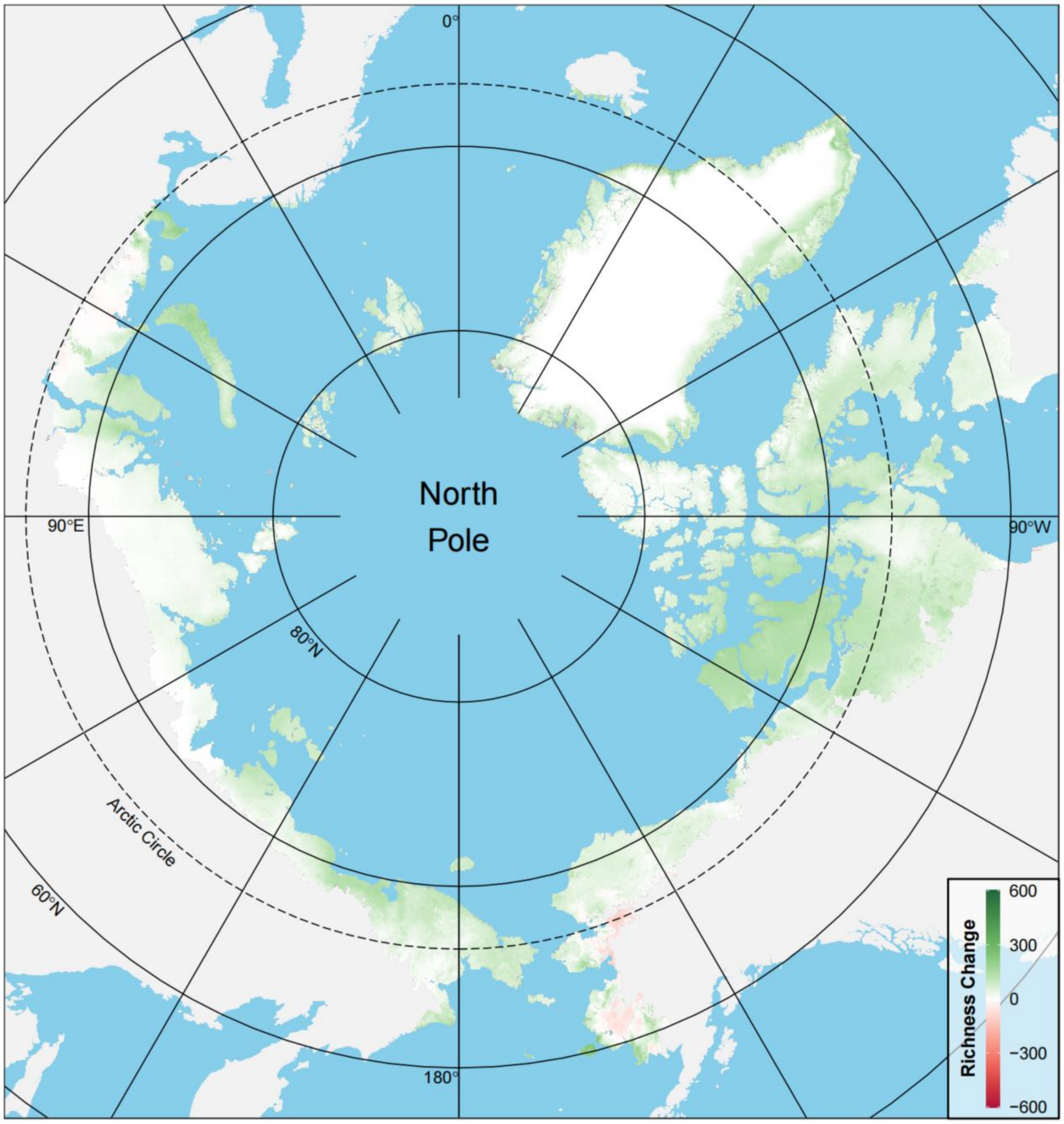

Fig. S24. Changes in the potential species richness under scenario 2030s SSP5-8.5 compared to the current climatic scenario. The colors from white to green indicate an increase in species richness, while from white to red indicate a decrease (from low to high, respectively).

Fig. S25.

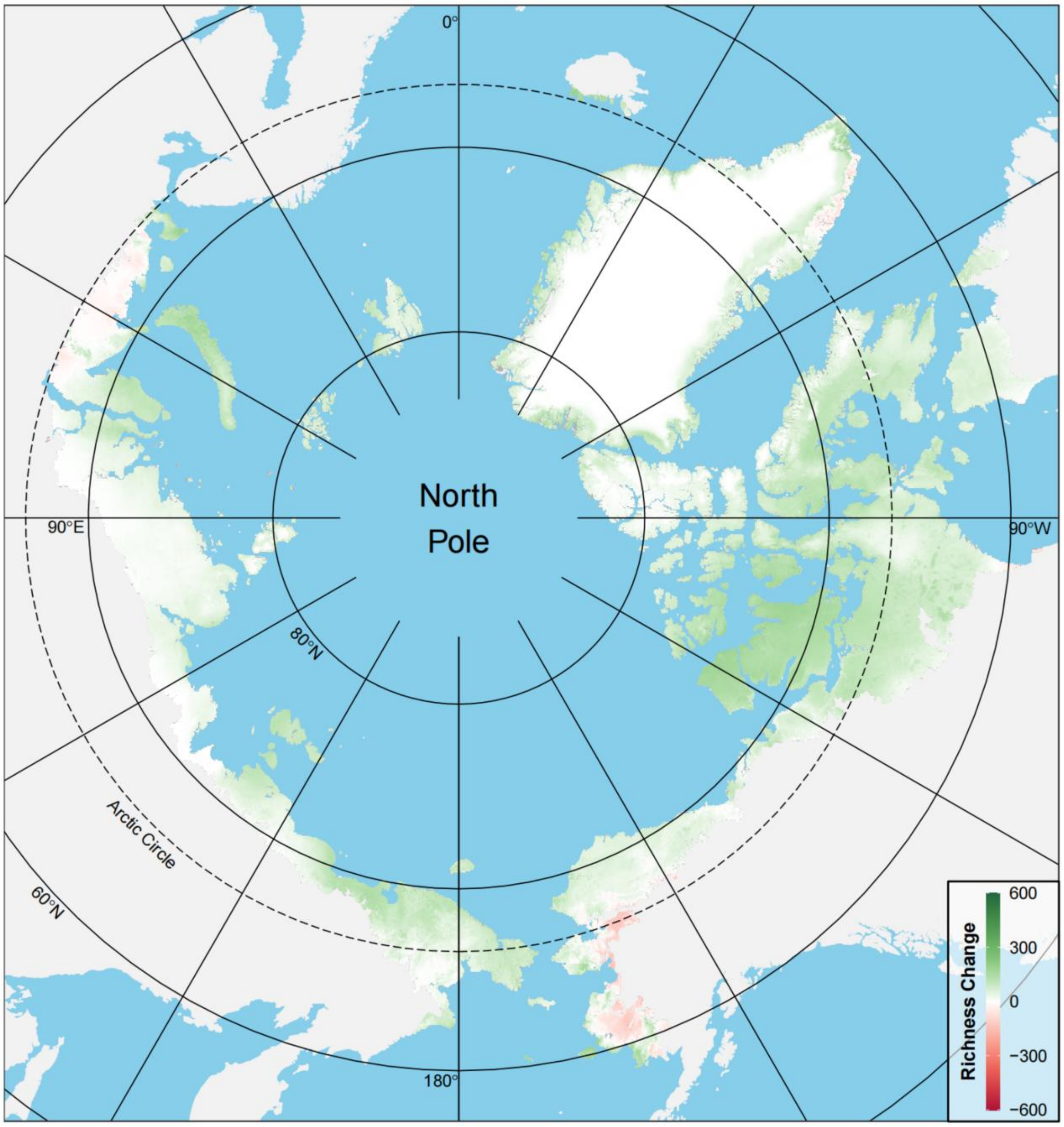

Fig. S25. Changes in the potential species richness under scenario 2050s SSP1-2.6 compared to the current climatic scenario. The colors from white to green indicate an increase in species richness, while from white to red indicate a decrease (from low to high, respectively).

Fig. S26.

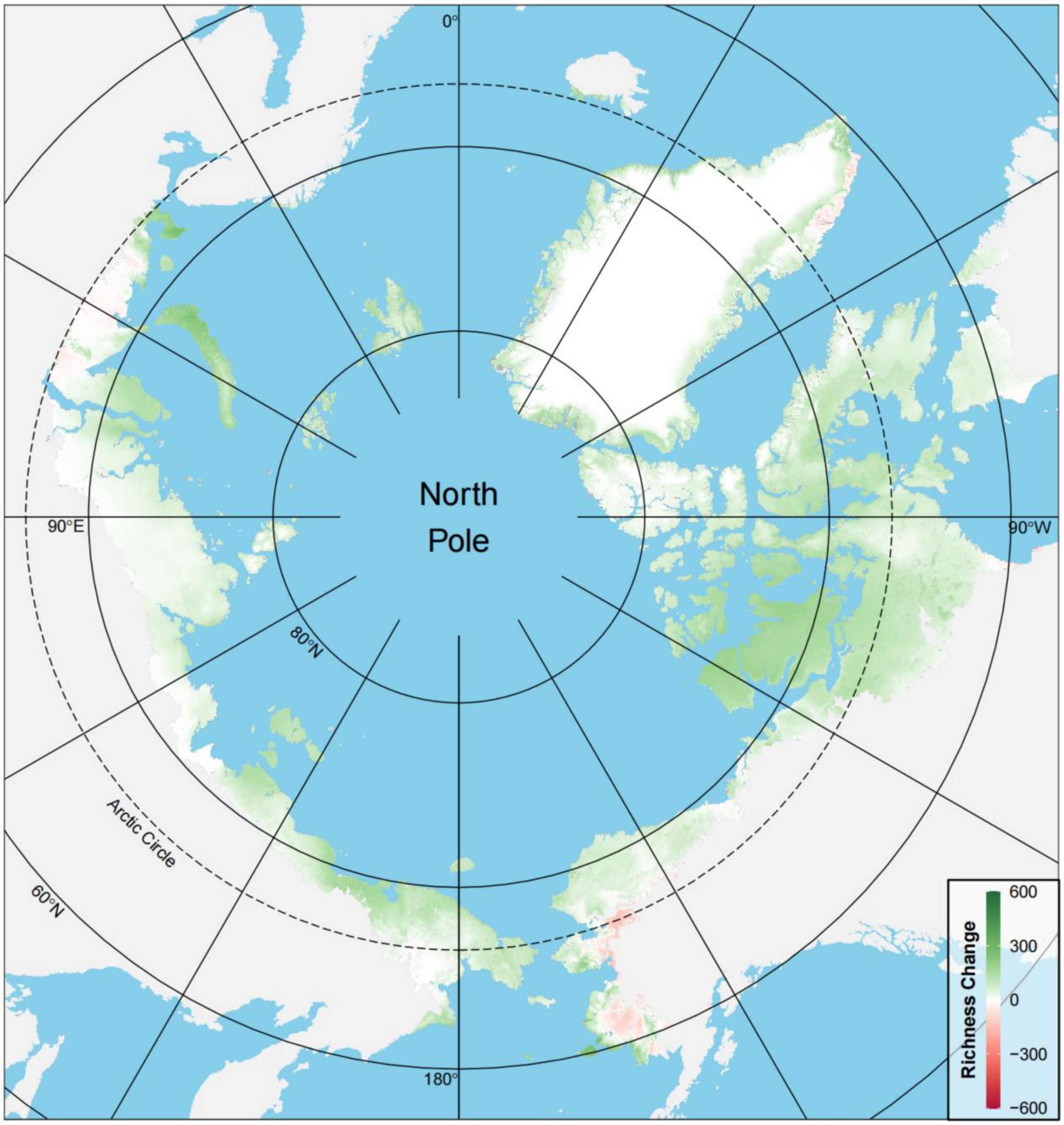

Fig. S26. Changes in the potential species richness under scenario 2050s SSP2-4.5 compared to the current climatic scenario. The colors from white to green indicate an increase in species richness, while from white to red indicate a decrease (from low to high, respectively).

Fig. S27.

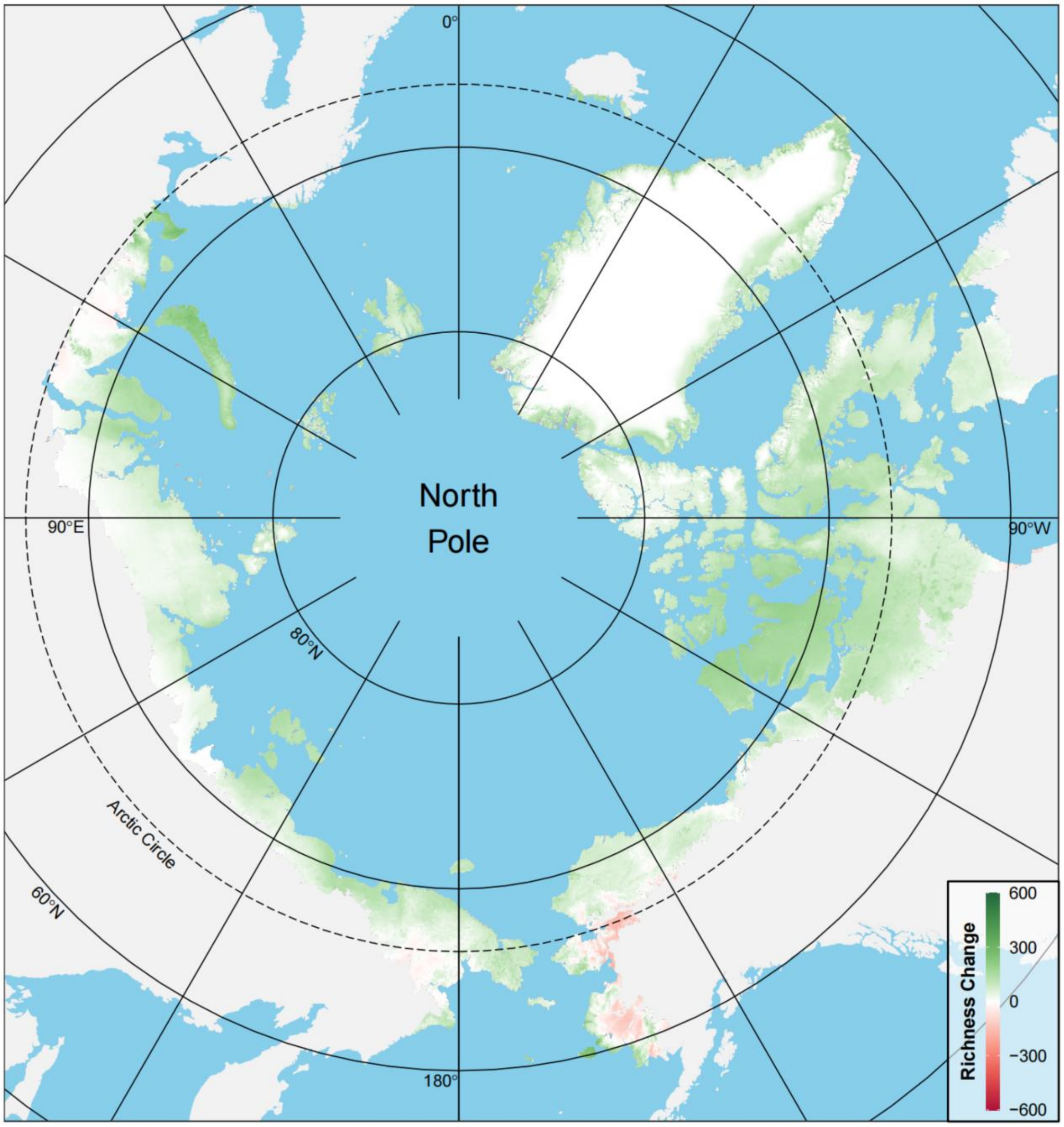

Fig. S27. Changes in the potential species richness under scenario 2050s SSP3-7.0 compared to the current climatic scenario. The colors from white to green indicate an increase in species richness, while from white to red indicate a decrease (from low to high, respectively).

Fig. S28.

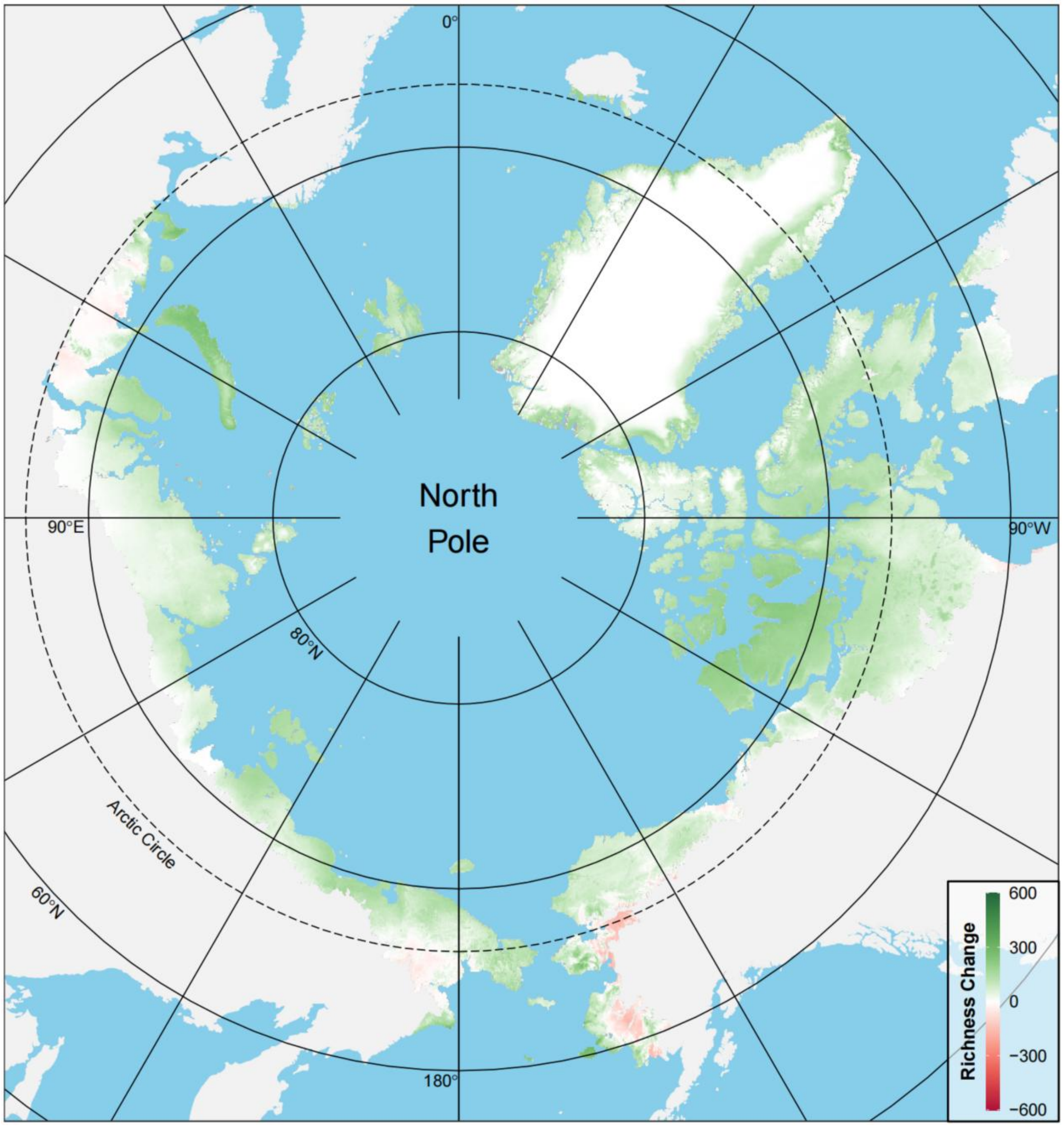

Fig. S28. Changes in the potential species richness under scenario 2050s SSP5-8.5 compared to the current climatic scenario. The colors from white to green indicate an increase in species richness, while from white to red indicate a decrease (from low to high, respectively).

Fig. S29.

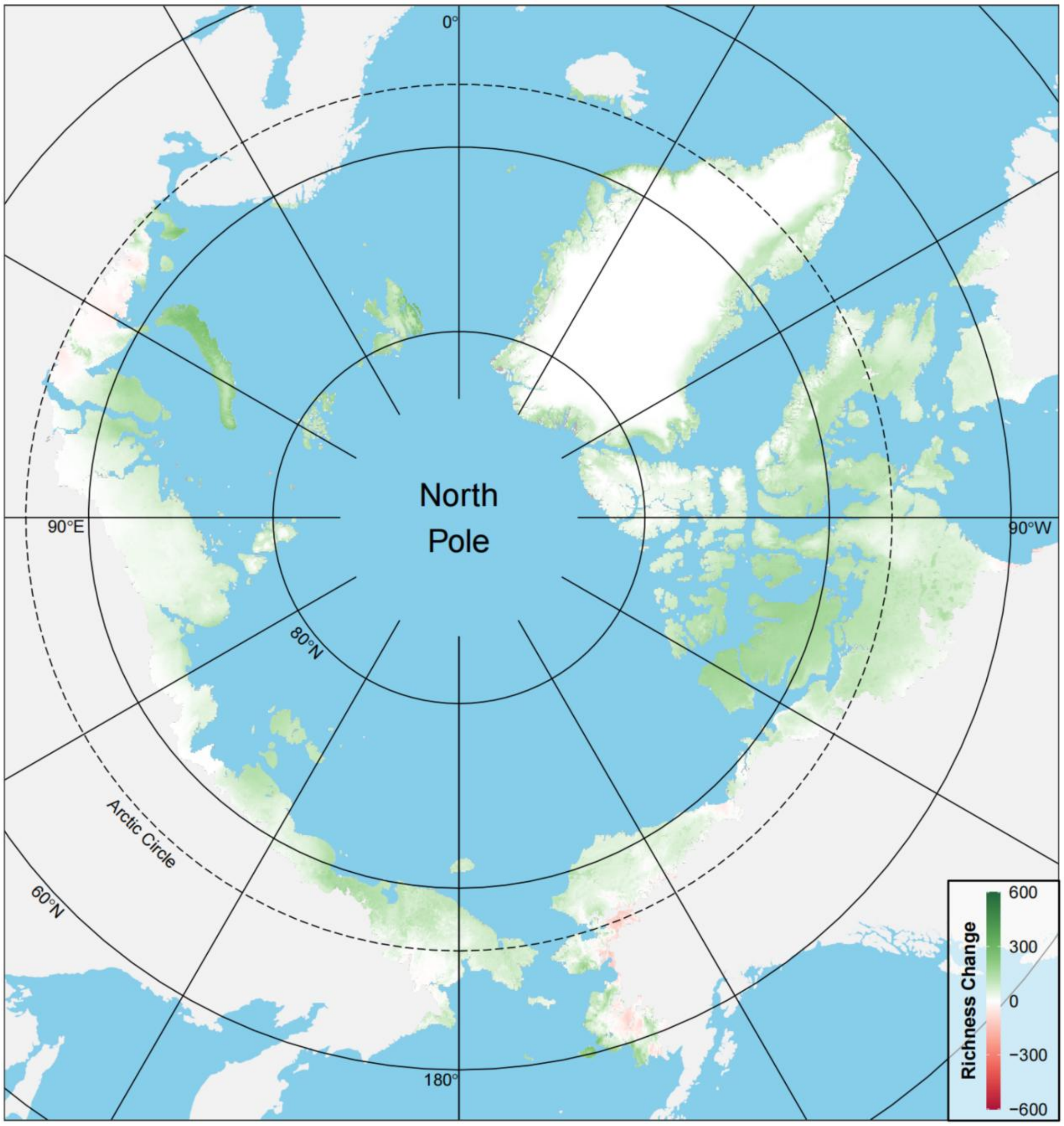

Fig. S29. Changes in the potential species richness under scenario 2070s SSP1-2.6 compared to the current climatic scenario. The colors from white to green indicate an increase in species richness, while from white to red indicate a decrease (from low to high, respectively).

Fig. S30.

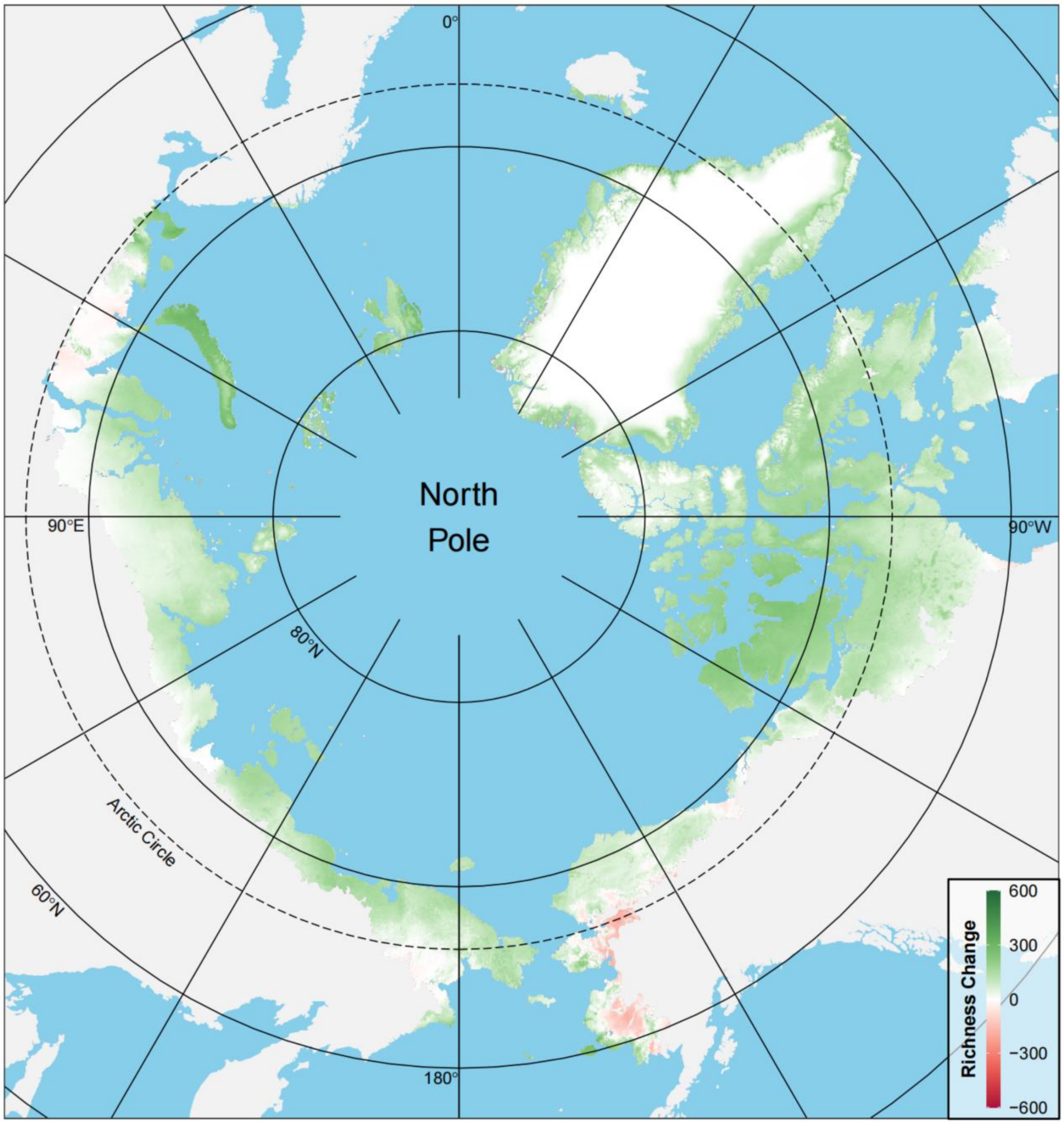

Fig. S30. Changes in the potential species richness under scenario 2070s SSP2-4.5 compared to the current climatic scenario. The colors from white to green indicate an increase in species richness, while from white to red indicate a decrease (from low to high, respectively).

Fig. S31.

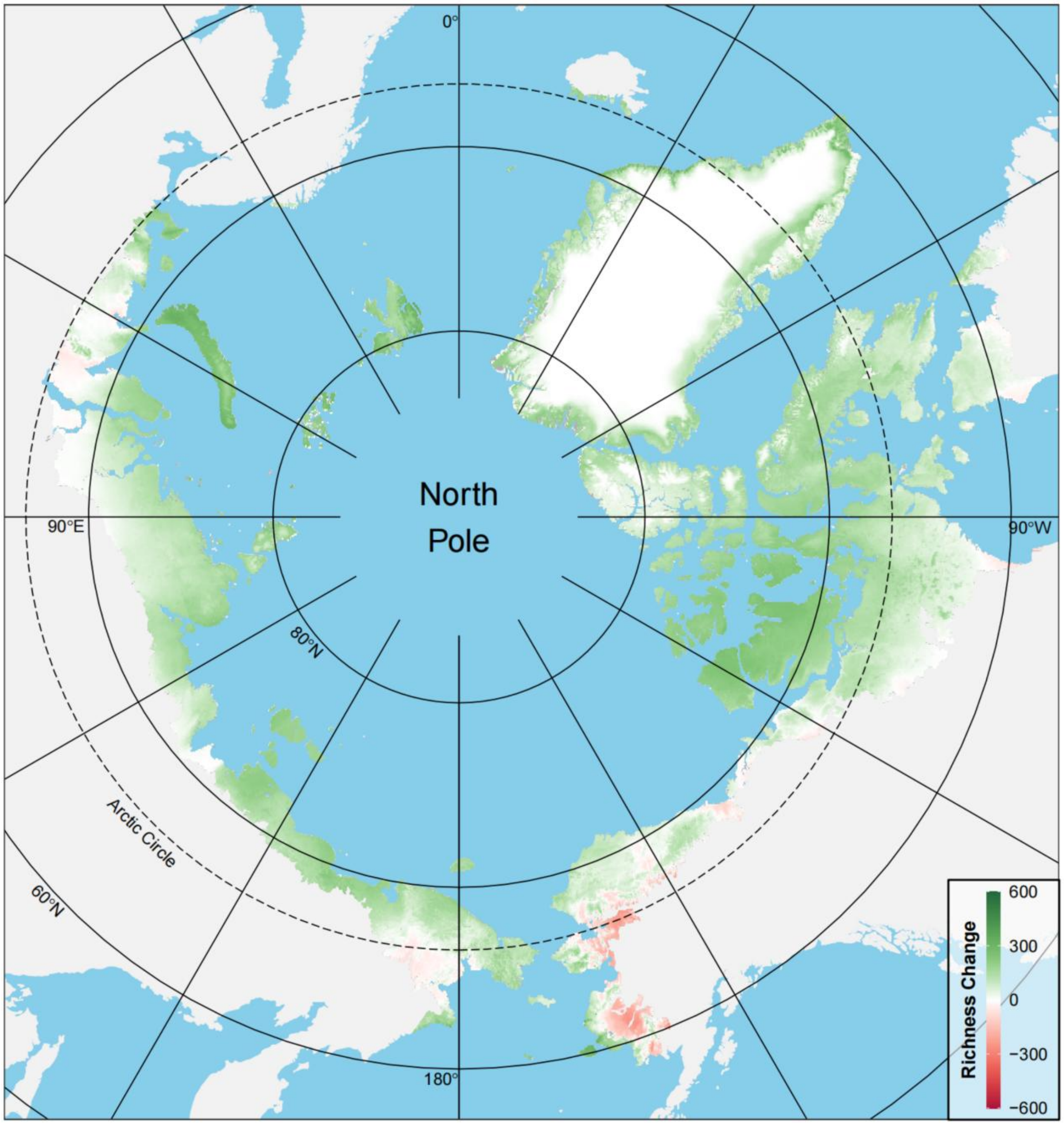

Fig. S31. Changes in the potential species richness under scenario 2070s SSP3-7.0 compared to the current climatic scenario. The colors from white to green indicate an increase in species richness, while from white to red indicate a decrease (from low to high, respectively).

Fig. S32.

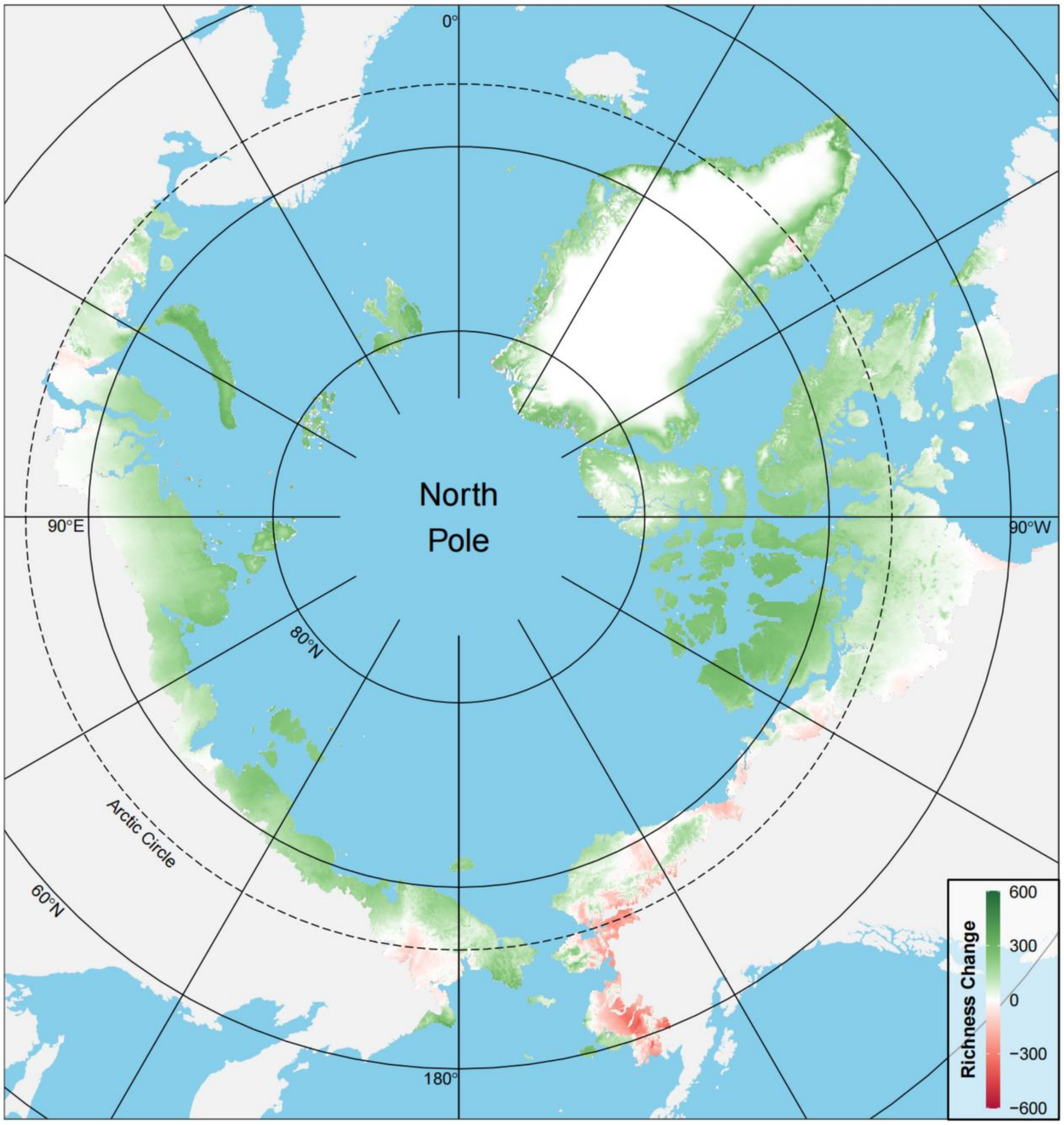

Fig. S32. Changes in the potential species richness under scenario 2070s SSP5-8.5 compared to the current climatic scenario. The colors from white to green indicate an increase in species richness, while from white to red indicate a decrease (from low to high, respectively).

Fig. S33.

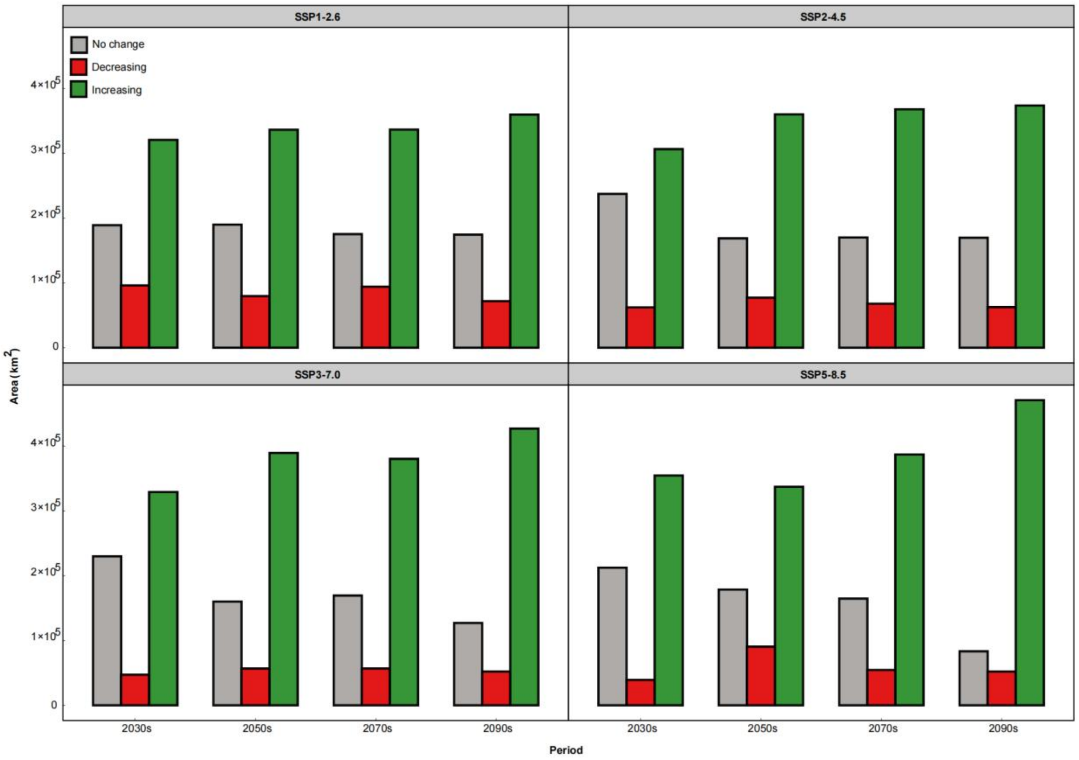

Fig. S33. Frequency distribution histogram of species richness changes in European Russia-West Siberia sector under future climate scenarios compared with the current climatic scenario.

Fig. S34.

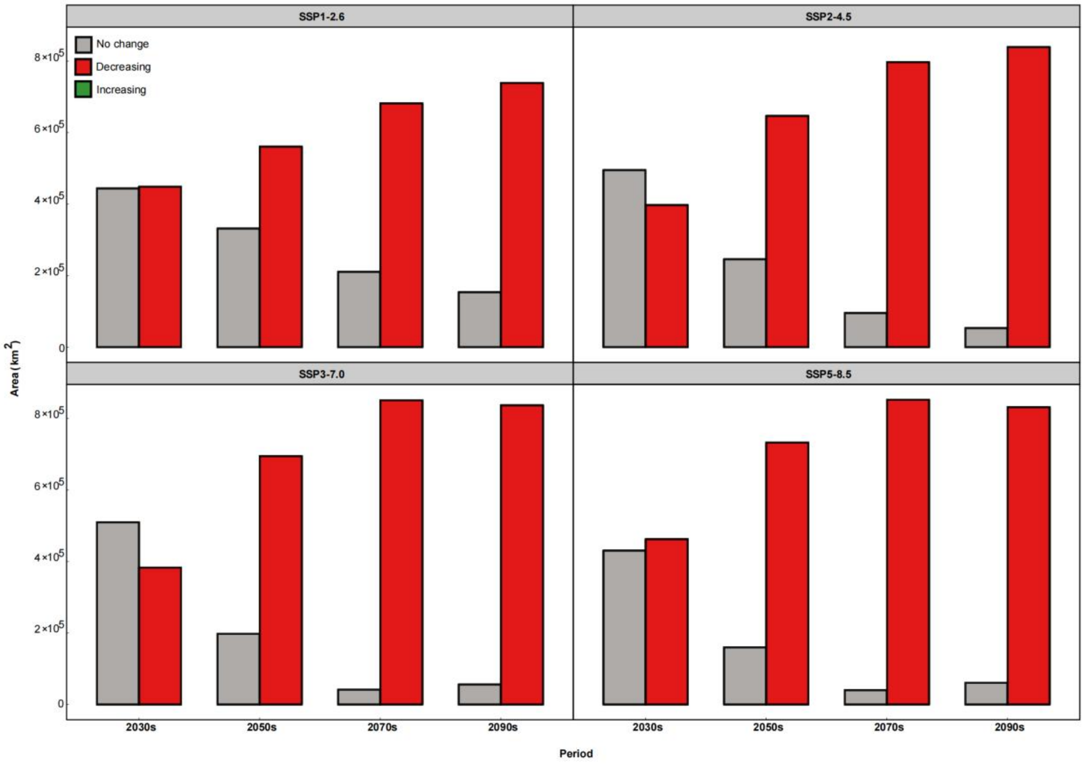

Fig. S34. Frequency distribution histogram of species richness changes in East Siberia sector under future climate scenarios compared with the current climatic scenario.

Fig. S35.

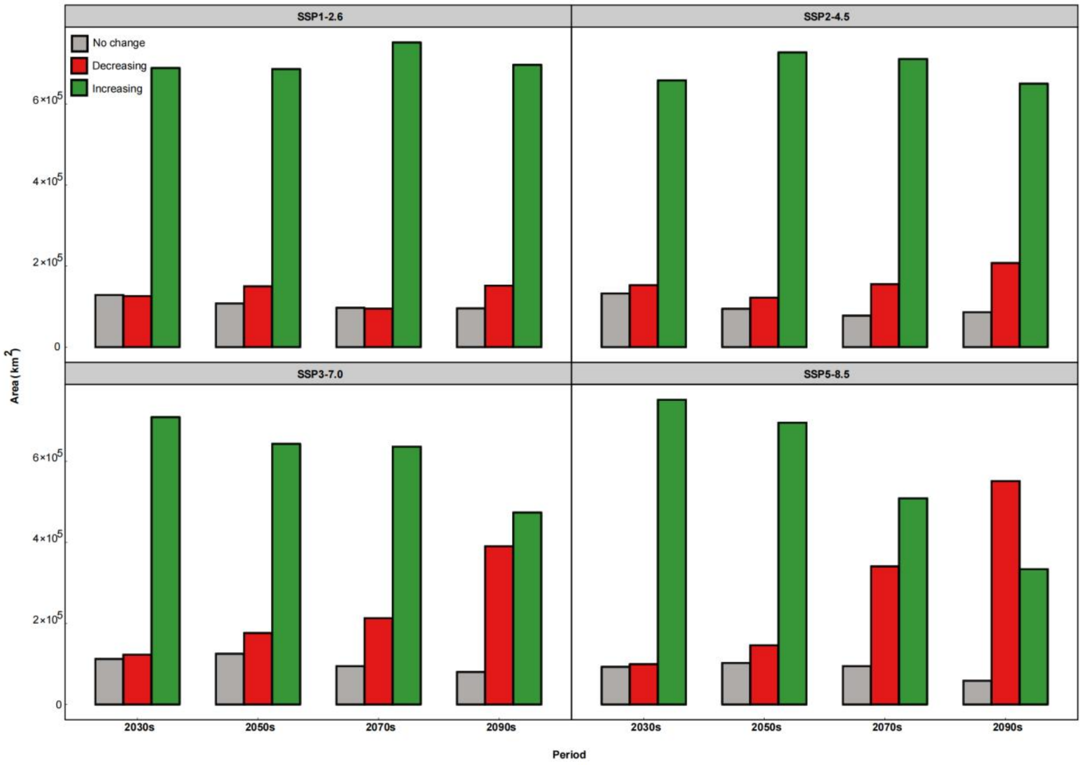

Fig. S35. Frequency distribution histogram of species richness changes in Beringia sector under future climate scenarios compared with the current climatic scenario.

Fig. S36.

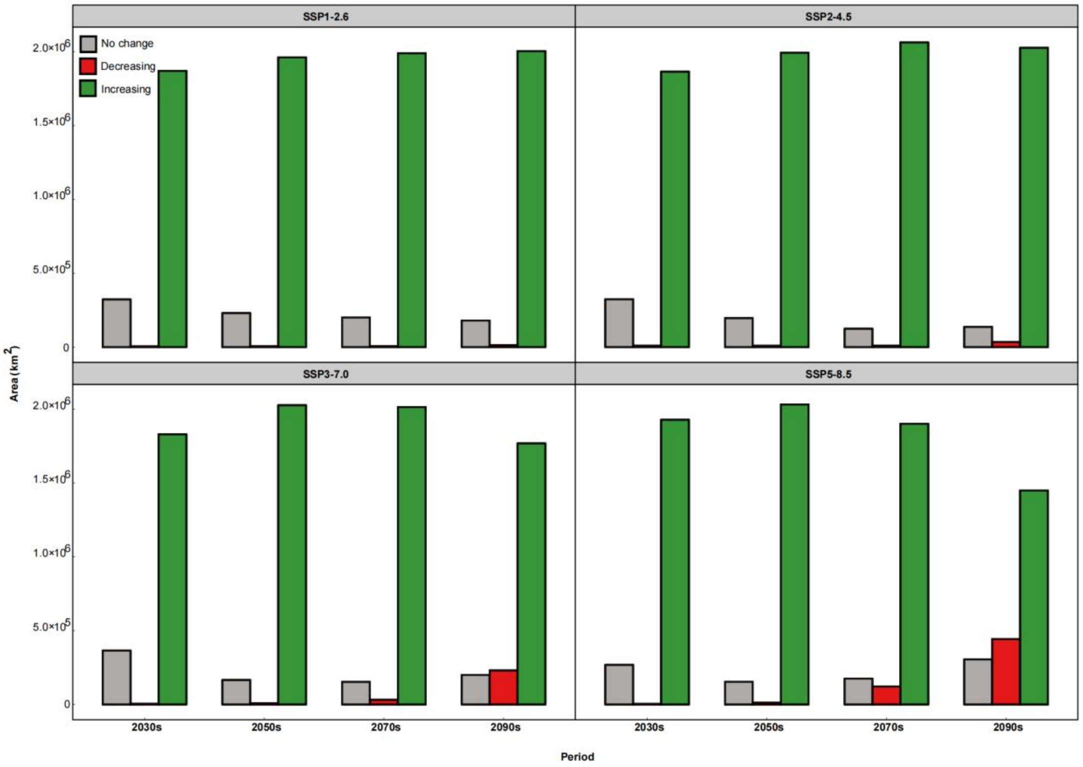

Fig. S36. Frequency distribution histogram of species richness changes in Canada sector under future climate scenarios compared with the current climatic scenario.

Fig. S37.

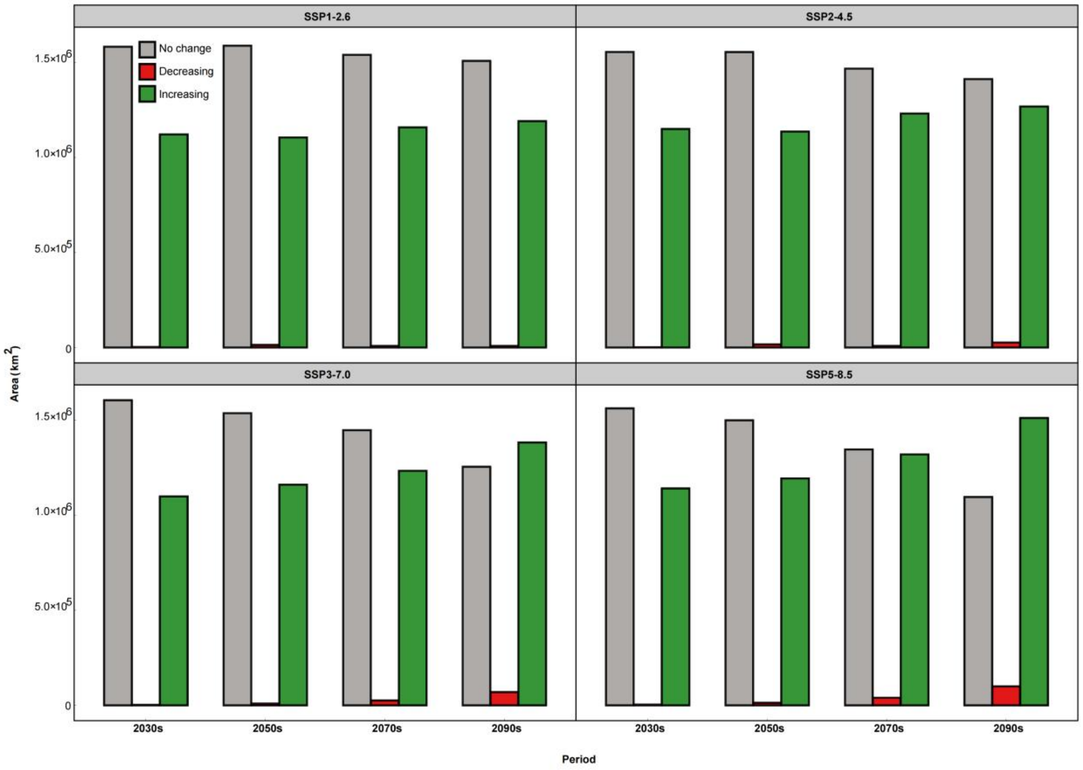

Fig. S37. Frequency distribution histogram of species richness changes in the North Atlantic sector under future climate scenarios compared with the current climatic scenario**.**

Fig. S38.

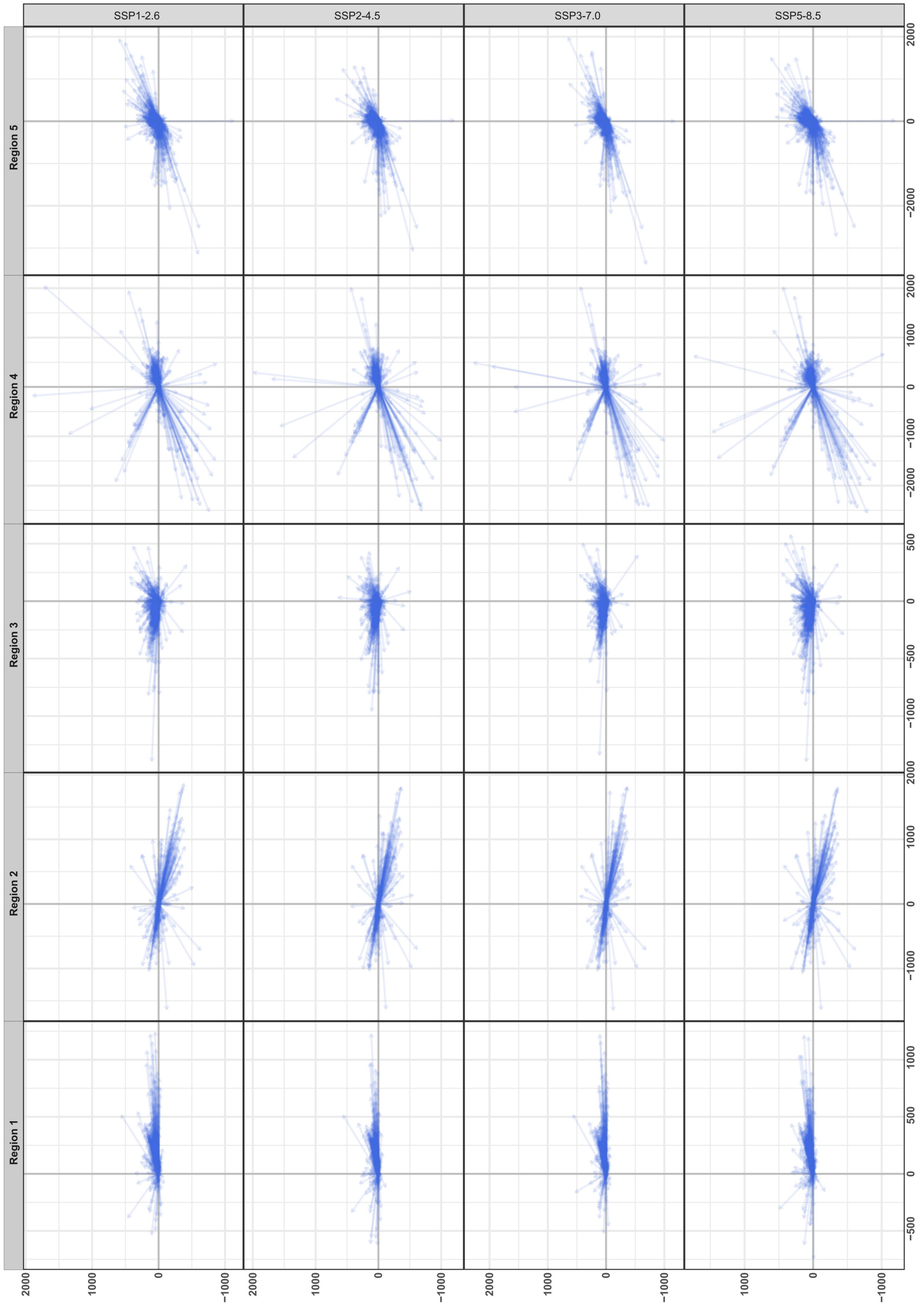

Fig. S38. The distance (magnitude) and direction of changes (represented by the arrows) at the

distributional centroid of species in each Arctic floristic sectors and scenario from the current to 2030s. The arrows indicate the magnitude and direction of predicted species' mean distributional centroid change through time. Region 1: European Russia-West Siberia, Region 2: East Siberia, Region 3: Beringia, Region 4: Canada, and Region 5: North Atlantic.

Fig. S39.

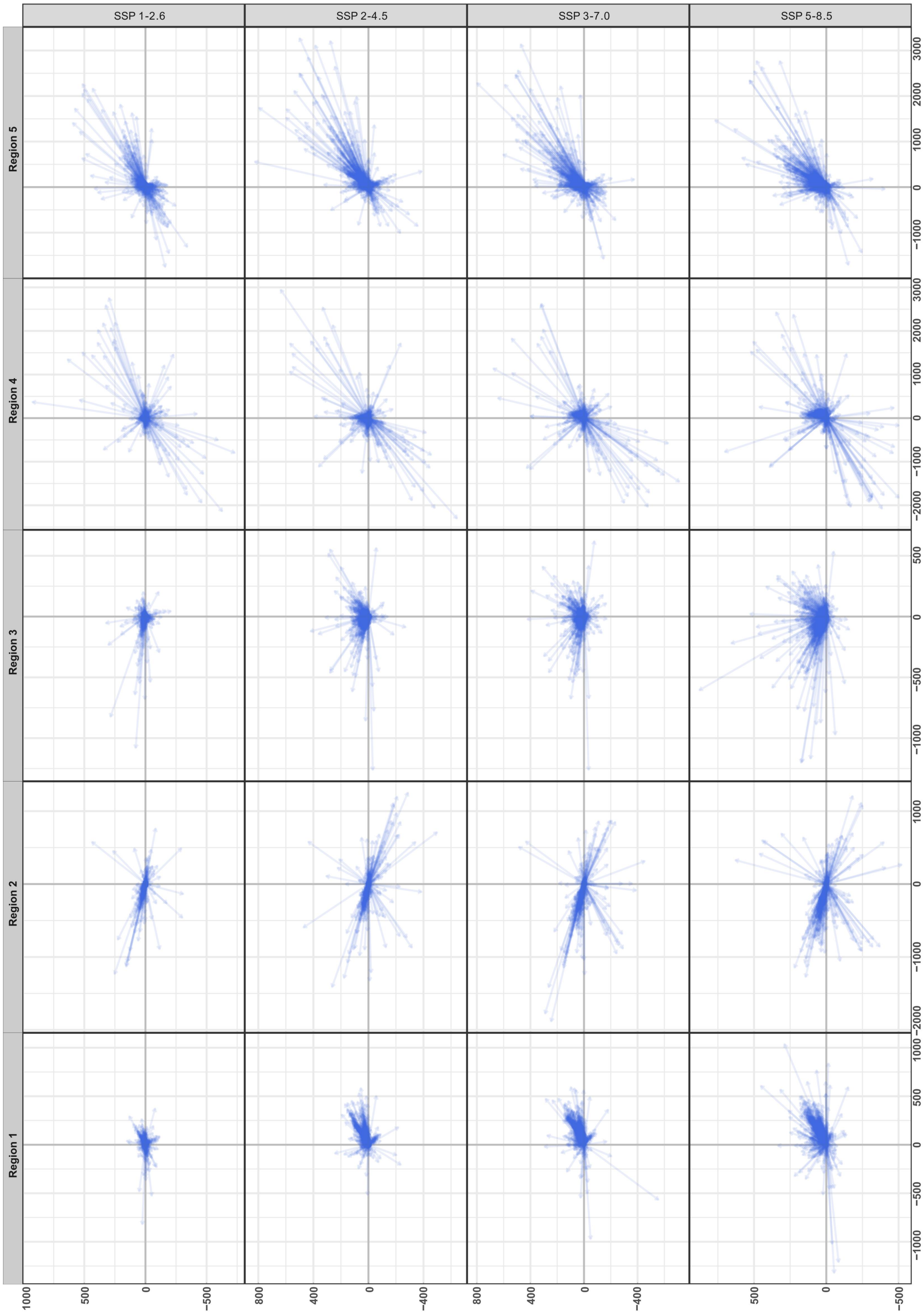

Fig. S39. The distance (magnitude) and direction of changes (represented by the arrows) at the

distributional centroid of species in each Arctic floristic sectors and scenario from the current to 2050s. The arrows indicate the magnitude and direction of predicted species' mean distributional centroid change through time. Region 1: European Russia-West Siberia, Region 2: East Siberia, Region 3: Beringia, Region 4: Canada, and Region 5: North Atlantic.

Fig. S40.

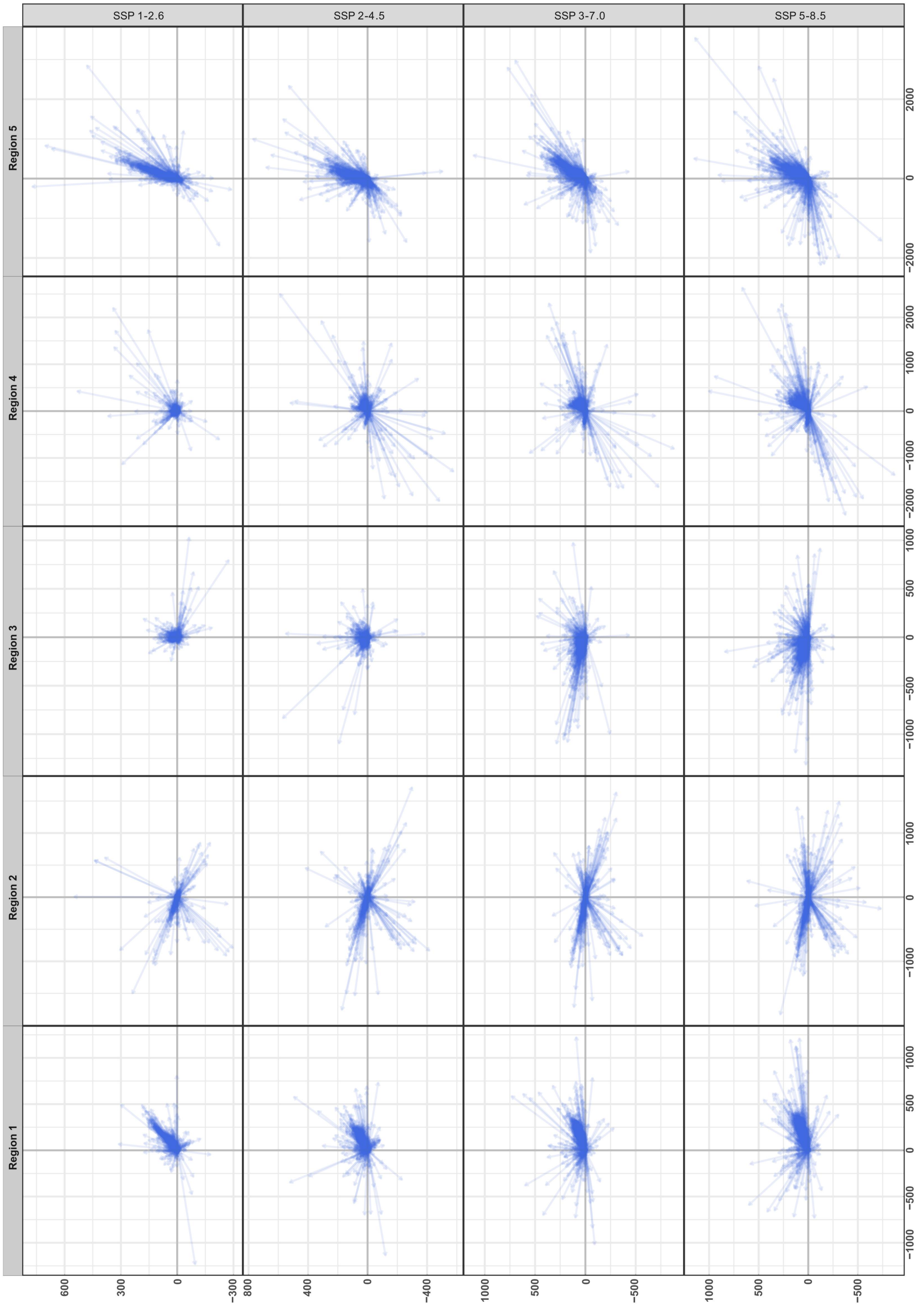

Fig. S40. The distance (magnitude) and direction of changes (represented by the arrows) at the distributional centroid of species in each Arctic floristic sectors and scenario from the current to

the 2070s. The arrows indicate the magnitude and direction of predicted species' mean distributional centroid change through time. Region 1: European Russia-West Siberia, Region 2: East Siberia, Region 3: Beringia, Region 4: Canada, and Region 5: North Atlantic.

Fig. S41.

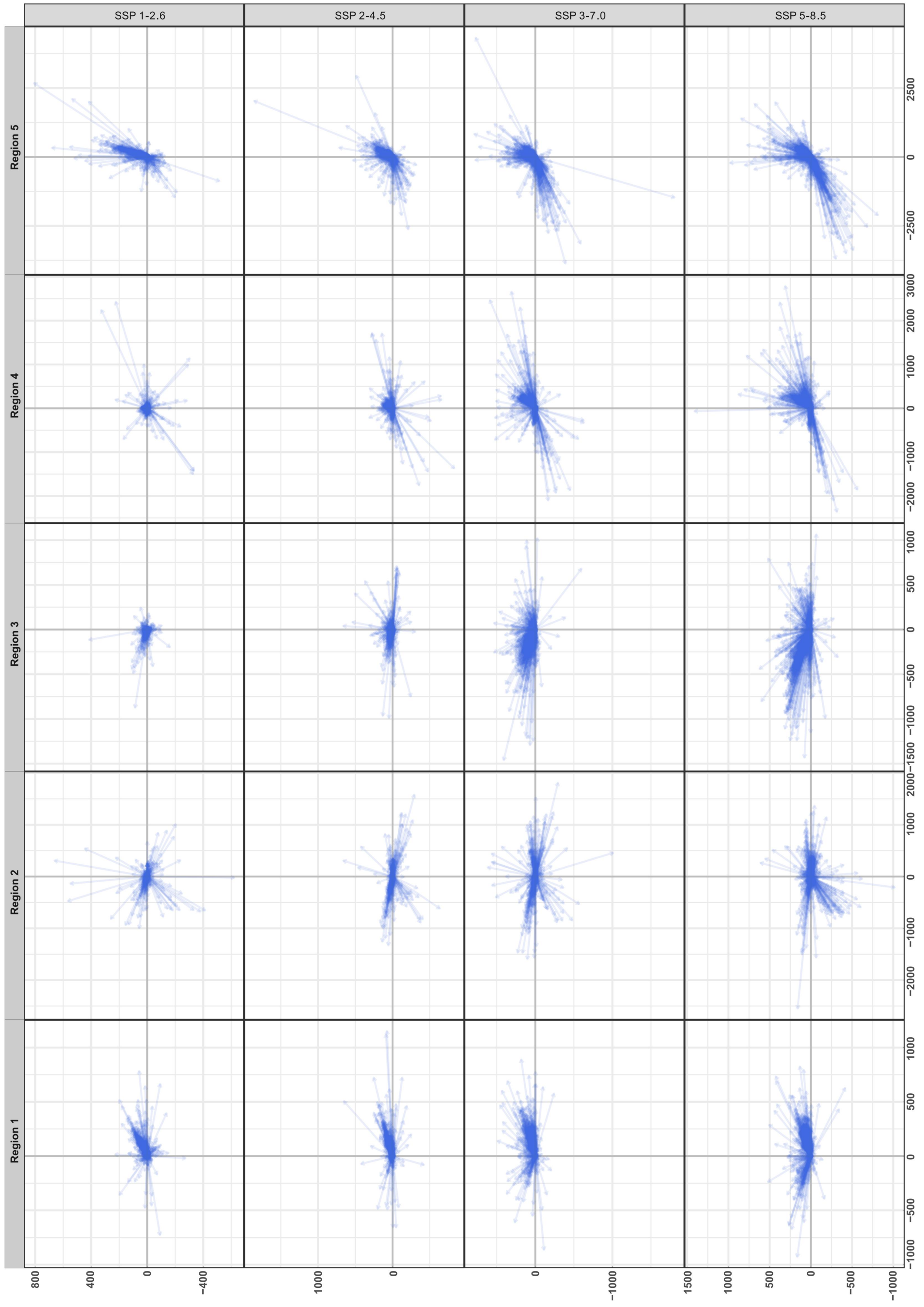

Fig. S41. The distance (magnitude) and direction of changes (represented by the arrows) at the distributional centroid of species in Arctic each Arctic floristic sectors and scenario from the

current to 2090s. The arrows indicate the magnitude and direction of predicted species' mean distributional centroid change through time. Region 1: European Russia-West Siberia, Region 2: East Siberia, Region 3: Beringia, Region 4: Canada, and Region 5: North Atlantic.

Table S1. The numbers of species with expansion and contraction of the Area of Habitat (AOH) in the Arctic compared to the present day

SSPs	Period	No. species with range expansion	No. species with range contraction
SSP1-2.6	2030s	217	2
SSP1-2.6	2050s	245	2
SSP1-2.6	2070s	289	2
SSP1-2.6	2090s	306	5
SSP2-4.5	2030s	206	2
SSP2-4.5	2050s	267	4
SSP2-4.5	2070s	341	6
SSP2-4.5	2090s	374	8
SSP3-7.0	2030s	191	3
SSP3-7.0	2050s	279	3
SSP3-7.0	2070s	357	8
SSP3-7.0	2090s	481	23
SSP5-8.5	2030s	233	3
SSP5-8.5	2050s	315	6
SSP5-8.5	2070s	390	18
SSP5-8.5	2090s	512	53

Note. The species' AOH are considered to be decreasing/increasing when the range change is >5% area of the Arctic.

Table S2. The mean Area of Habitat (AOH) of species in the Arctic now and in the future under four GHG emission scenarios

SSPs	Period	Mean AOH (km^2)	Standard deviation	Standard error	Confidence interval
–	Current	311435.4	515566.8	14964.4	29359.7
SSP1-2.6	2030s	500362.9	785554.8	22800.9	44734.5
SSP1-2.6	2050s	535931.4	837090.7	24296.7	47669.3
SSP1-2.6	2070s	579755.5	876190.9	25431.6	49895.9
SSP1-2.6	2090s	614969.8	905678.1	26287.5	51575.1
SSP2-4.5	2030s	492782.9	771375.6	22389.3	43927.1
SSP2-4.5	2050s	563595.8	858305.8	24912.5	48877.4
SSP2-4.5	2070s	685343.3	976629.3	28346.8	55615.5
SSP2-4.5	2090s	741606.1	992162.1	28797.7	56500.0
SSP3-7.0	2030s	474712.3	746809.8	21676.3	42528.1
SSP3-7.0	2050s	580962.0	882154.2	25604.7	50235.5
SSP3-7.0	2070s	704638.9	966695.1	28058.5	55049.8
SSP3-7.0	2090s	794238.1	945494.3	27443.1	53842.5
SSP5-8.5	2030s	523460.7	808631.4	23470.7	46048.6
SSP5-8.5	2050s	617715.6	897597.8	26052.9	51114.9
SSP5-8.5	2070s	751318.8	966171.4	28043.3	55020.0
SSP5-8.5	2090s	743103.0	808576.5	23469.1	46045.5

Table S3. The mean potential species richness in the Arctic at the current and future under four GHG emission scenarios

SSPs	Period	Potential species richness (spgc)	Standard deviation (spgc)	Standard error	Confidence interval
–	Current	52.4	86.7	0.1	0.2
SSP1-2.6	2030s	84.1	97.9	0.1	0.2
SSP1-2.6	2050s	90.1	97.7	0.1	0.2
SSP1-2.6	2070s	97.5	103.4	0.1	0.2
SSP1-2.6	2090s	103.4	105.0	0.1	0.2
SSP2-4.5	2030s	82.9	97.9	0.1	0.2
SSP2-4.5	2050s	94.8	101.2	0.1	0.2
SSP2-4.5	2070s	115.2	109.4	0.1	0.2
SSP2-4.5	2090s	124.6	113.6	0.1	0.3
SSP3-7.0	2030s	79.8	96.6	0.1	0.2
SSP3-7.0	2050s	97.7	101.0	0.1	0.2
SSP3-7.0	2070s	118.4	109.3	0.1	0.2
SSP3-7.0	2090s	133.4	123.4	0.1	0.3
SSP5-8.5	2030s	88.0	100.8	0.1	0.2
SSP5-8.5	2050s	103.8	105.8	0.1	0.2
SSP5-8.5	2070s	126.2	114.2	0.1	0.3
SSP5-8.5	2090s	124.7	128.3	0.1	0.3

Note. spgc = species per grid cell.

Table S4. The numbers of species with expansion and contraction of the Area of Habitat (AOH) in Arctic floristic sectors compared to now

Region	SSPs	Period	No. species with range expansion	No. species with range contraction
Region 1	SSP1-2.6	2030s	198	29
Region 1	SSP1-2.6	2050s	233	41
Region 1	SSP1-2.6	2070s	327	40
Region 1	SSP1-2.6	2090s	453	49
Region 1	SSP2-4.5	2030s	202	25
Region 1	SSP2-4.5	2050s	300	36
Region 1	SSP2-4.5	2070s	476	57
Region 1	SSP2-4.5	2090s	534	87
Region 1	SSP3-7.0	2030s	194	23
Region 1	SSP3-7.0	2050s	341	34
Region 1	SSP3-7.0	2070s	540	79
Region 1	SSP3-7.0	2090s	550	124
Region 1	SSP5-8.5	2030s	250	21
Region 1	SSP5-8.5	2050s	384	50
Region 1	SSP5-8.5	2070s	554	105
Region 1	SSP5-8.5	2090s	500	142
Region 2	SSP1-2.6	2030s	136	4
Region 2	SSP1-2.6	2050s	142	5
Region 2	SSP1-2.6	2070s	172	5
Region 2	SSP1-2.6	2090s	201	6
Region 2	SSP2-4.5	2030s	113	3
Region 2	SSP2-4.5	2050s	171	4
Region 2	SSP2-4.5	2070s	206	5
Region 2	SSP2-4.5	2090s	268	7
Region 2	SSP3-7.0	2030s	110	4
Region 2	SSP3-7.0	2050s	167	5
Region 2	SSP3-7.0	2070s	254	5
Region 2	SSP3-7.0	2090s	373	8
Region 2	SSP5-8.5	2030s	138	5
Region 2	SSP5-8.5	2050s	187	5
Region 2	SSP5-8.5	2070s	332	7
Region 2	SSP5-8.5	2090s	425	11
Region 3	SSP1-2.6	2030s	298	59
Region 3	SSP1-2.6	2050s	299	59
Region 3	SSP1-2.6	2070s	388	58
Region 3	SSP1-2.6	2090s	390	85
Region 3	SSP2-4.5	2030s	281	61

Region 3	SSP2-4.5	2050s	391	86
Region 3	SSP2-4.5	2070s	422	111
Region 3	SSP2-4.5	2090s	443	171
Region 3	SSP3-7.0	2030s	267	59
Region 3	SSP3-7.0	2050s	340	127
Region 3	SSP3-7.0	2070s	433	175
Region 3	SSP3-7.0	2090s	438	272
Region 3	SSP5-8.5	2030s	339	49
Region 3	SSP5-8.5	2050s	444	125
Region 3	SSP5-8.5	2070s	426	234
Region 3	SSP5-8.5	2090s	403	361
Region 4	SSP1-2.6	2030s	224	4
Region 4	SSP1-2.6	2050s	247	5
Region 4	SSP1-2.6	2070s	253	4
Region 4	SSP1-2.6	2090s	265	6
Region 4	SSP2-4.5	2030s	209	3
Region 4	SSP2-4.5	2050s	259	6
Region 4	SSP2-4.5	2070s	310	6
Region 4	SSP2-4.5	2090s	313	9
Region 4	SSP3-7.0	2030s	201	4
Region 4	SSP3-7.0	2050s	272	6
Region 4	SSP3-7.0	2070s	301	10
Region 4	SSP3-7.0	2090s	365	13
Region 4	SSP5-8.5	2030s	236	5
Region 4	SSP5-8.5	2050s	283	5
Region 4	SSP5-8.5	2070s	330	12
Region 4	SSP5-8.5	2090s	355	20
Region 5	SSP1-2.6	2030s	152	3
Region 5	SSP1-2.6	2050s	174	3
Region 5	SSP1-2.6	2070s	232	3
Region 5	SSP1-2.6	2090s	239	3
Region 5	SSP2-4.5	2030s	158	1
Region 5	SSP2-4.5	2050s	182	3
Region 5	SSP2-4.5	2070s	290	3
Region 5	SSP2-4.5	2090s	324	5
Region 5	SSP3-7.0	2030s	122	2
Region 5	SSP3-7.0	2050s	219	3
Region 5	SSP3-7.0	2070s	287	5
Region 5	SSP3-7.0	2090s	431	12
Region 5	SSP5-8.5	2030s	154	3
Region 5	SSP5-8.5	2050s	233	4
Region 5	SSP5-8.5	2070s	342	10
Region 5	SSP5-8.5	2090s	550	20

Note. The species' AOH were considered to be decreasing/increasing when the range change is >5% area of the Arctic floristic sector. Region 1: European Russia-West Siberia, Region 2: East Siberia, Region 3: Beringia, Region 4: Canada, and Region 5: North Atlantic.

Table S5. The mean Area of Habitat (AOH) of species in the Arctic floristic sectors now and in the future under four GHG emission scenarios

Region	SSPs	Period	Mean AOH (km^2)	Standard deviation (km^2)	Standard error	Confidence interval
Region 1	–	Current	20253.0	49402.5	1433.9	2813.3
Region 1	SSP1-2.6	2030s	32077.1	65282.6	1894.8	3717.6
Region 1	SSP1-2.6	2050s	34609.0	66607.0	1933.3	3793.0
Region 1	SSP1-2.6	2070s	42784.2	70207.8	2037.8	3998.1
Region 1	SSP1-2.6	2090s	51474.0	71734.2	2082.1	4085.0
Region 1	SSP2-4.5	2030s	32377.5	64113.8	1860.9	3651.0
Region 1	SSP2-4.5	2050s	40917.9	69962.2	2030.7	3984.1
Region 1	SSP2-4.5	2070s	52374.8	70568.8	2048.3	4018.6
Region 1	SSP2-4.5	2090s	58091.0	72328.0	2099.3	4118.8
Region 1	SSP3-7.0	2030s	32287.6	67220.7	1951.1	3828.0
Region 1	SSP3-7.0	2050s	45534.3	74347.0	2157.9	4233.8
Region 1	SSP3-7.0	2070s	58534.1	71733.6	2082.1	4085.0
Region 1	SSP3-7.0	2090s	65065.0	81484.0	2365.1	4640.2
Region 1	SSP5-8.5	2030s	36825.3	69637.0	2021.2	3965.6
Region 1	SSP5-8.5	2050s	44621.0	68307.1	1982.6	3889.8
Region 1	SSP5-8.5	2070s	62206.5	75073.6	2179.0	4275.2
Region 1	SSP5-8.5	2090s	63792.7	91318.7	2650.5	5200.3
Region 2	–	Current	4071.0	32515.7	943.8	1851.7
Region 2	SSP1-2.6	2030s	20428.3	62103.8	1802.6	3536.6
Region 2	SSP1-2.6	2050s	24156.0	70623.9	2049.9	4021.8
Region 2	SSP1-2.6	2070s	32815.1	89585.6	2600.2	5101.6
Region 2	SSP1-2.6	2090s	41749.7	106618.2	3094.6	6071.5
Region 2	SSP2-4.5	2030s	16982.5	56563.0	1641.7	3221.1
Region 2	SSP2-4.5	2050s	31032.7	85004.2	2467.3	4840.7
Region 2	SSP2-4.5	2070s	46240.6	115319.7	3347.2	6567.0
Region 2	SSP2-4.5	2090s	66482.1	145507.2	4223.4	8286.1
Region 2	SSP3-7.0	2030s	16496.2	54812.6	1590.9	3121.4
Region 2	SSP3-7.0	2050s	33423.8	92068.2	2672.3	5243.0
Region 2	SSP3-7.0	2070s	62780.5	141572.3	4109.2	8062.0
Region 2	SSP3-7.0	2090s	92686.7	163599.4	4748.5	9316.4
Region 2	SSP5-8.5	2030s	20604.9	61288.4	1778.9	3490.2
Region 2	SSP5-8.5	2050s	39321.8	103357.9	3000.0	5885.9
Region 2	SSP5-8.5	2070s	81319.9	161344.9	4683.1	9188.0
Region 2	SSP5-8.5	2090s	87147.5	140987.7	4092.2	8028.7
Region 3	–	Current	145273.1	207493.0	6022.5	11816.0
Region 3	SSP1-2.6	2030s	171191.8	224350.6	6511.8	12776.0
Region 3	SSP1-2.6	2050s	170303.4	224068.4	6503.6	12759.9

Region 3	SSP1-2.6	2070s	179393.1	226439.7	6572.4	12894.9
Region 3	SSP1-2.6	2090s	178549.9	228071.6	6619.8	12987.9
Region 3	SSP2-4.5	2030s	168415.9	225182.9	6536.0	12823.4
Region 3	SSP2-4.5	2050s	177585.7	221603.9	6432.1	12619.5
Region 3	SSP2-4.5	2070s	180275.3	217790.7	6321.4	12402.4
Region 3	SSP2-4.5	2090s	175497.9	201489.0	5848.2	11474.1
Region 3	SSP3-7.0	2030s	169099.7	221666.0	6433.9	12623.1
Region 3	SSP3-7.0	2050s	167271.6	207477.6	6022.1	11815.1
Region 3	SSP3-7.0	2070s	172977.8	200254.9	5812.4	11403.8
Region 3	SSP3-7.0	2090s	152344.2	162198.0	4707.8	9236.6
Region 3	SSP5-8.5	2030s	177002.6	229522.9	6661.9	13070.5
Region 3	SSP5-8.5	2050s	180112.1	208336.9	6047.0	11864.0
Region 3	SSP5-8.5	2070s	157485.7	176296.8	5117.0	10039.5
Region 3	SSP5-8.5	2090s	114256.3	123175.9	3575.2	7014.4
Region 4	–	Current	60613.0	175928.8	5106.4	10018.5
Region 4	SSP1-2.6	2030s	151494.1	336572.6	9769.1	19166.6
Region 4	SSP1-2.6	2050s	177525.6	376142.9	10917.6	21420.0
Region 4	SSP1-2.6	2070s	181483.8	382175.9	11092.7	21763.5
Region 4	SSP1-2.6	2090s	191797.6	396181.1	11499.2	22561.1
Region 4	SSP2-4.5	2030s	145404.2	330017.6	9578.8	18793.3
Region 4	SSP2-4.5	2050s	183022.4	383248.0	11123.8	21824.6
Region 4	SSP2-4.5	2070s	239150.8	455717.4	13227.3	25951.5
Region 4	SSP2-4.5	2090s	254316.7	464788.6	13490.6	26468.0
Region 4	SSP3-7.0	2030s	136946.1	314087.7	9116.4	17886.2
Region 4	SSP3-7.0	2050s	193602.0	400528.9	11625.4	22808.7
Region 4	SSP3-7.0	2070s	239996.7	449535.7	13047.8	25599.4
Region 4	SSP3-7.0	2090s	266261.0	445772.7	12938.6	25385.1
Region 4	SSP5-8.5	2030s	161404.1	352723.5	10237.9	20086.3
Region 4	SSP5-8.5	2050s	205616.2	410251.1	11907.6	23362.3
Region 4	SSP5-8.5	2070s	256589.8	454015.4	13177.9	25854.5
Region 4	SSP5-8.5	2090s	230828.9	374043.4	10856.7	21300.4
Region 5	–	Current	81225.3	144604.1	4197.2	8234.7
Region 5	SSP1-2.6	2030s	125171.8	195750.3	5681.7	11147.3
Region 5	SSP1-2.6	2050s	129337.4	202759.6	5885.1	11546.4
Region 5	SSP1-2.6	2070s	143279.3	214990.6	6240.1	12242.9
Region 5	SSP1-2.6	2090s	151398.7	217590.2	6315.6	12391.0
Region 5	SSP2-4.5	2030s	129602.7	196420.5	5701.1	11185.4
Region 5	SSP2-4.5	2050s	131037.0	204537.7	5936.7	11647.7
Region 5	SSP2-4.5	2070s	167301.9	235281.6	6829.1	13398.4
Region 5	SSP2-4.5	2090s	187218.5	238843.5	6932.5	13601.3
Region 5	SSP3-7.0	2030s	119882.6	188911.3	5483.2	10757.8
Region 5	SSP3-7.0	2050s	141130.3	214230.3	6218.1	12199.6
Region 5	SSP3-7.0	2070s	170349.8	233154.5	6767.3	13277.3

Region 5	SSP3-7.0	2090s	217881.2	244880.0	7107.7	13945.0
Region 5	SSP5-8.5	2030s	127623.8	195286.5	5668.2	11120.9
Region 5	SSP5-8.5	2050s	148044.5	219252.0	6363.8	12485.6
Region 5	SSP5-8.5	2070s	193716.9	240742.7	6987.6	13709.4
Region 5	SSP5-8.5	2090s	247077.5	248297.1	7206.9	14139.6

Note. Region 1: European Russia-West Siberia, Region 2: East Siberia, Region 3: Beringia, Region 4: Canada, and Region 5: North Atlantic.

Table S6. The mean potential species richness in the Arctic floristic sectors now and in the future under four GHG emission scenarios

Region	SSPs	Period	Species richness (spgc)	Standard deviation (spgc)	Standard error	Confidence interval
Region 1	–	Current	41.4	48.1	0.2	0.4
Region 1	SSP1-2.6	2030s	65.5	67.4	0.3	0.5
Region 1	SSP1-2.6	2050s	70.7	74.9	0.3	0.6
Region 1	SSP1-2.6	2070s	87.4	98.3	0.4	0.8
Region 1	SSP1-2.6	2090s	105.1	121.2	0.5	0.9
Region 1	SSP2-4.5	2030s	66.1	71.9	0.3	0.6
Region 1	SSP2-4.5	2050s	83.6	91.6	0.4	0.7
Region 1	SSP2-4.5	2070s	107.0	127.0	0.5	1.0
Region 1	SSP2-4.5	2090s	118.6	141.2	0.6	1.1
Region 1	SSP3-7.0	2030s	65.9	66.4	0.3	0.5
Region 1	SSP3-7.0	2050s	93.0	97.8	0.4	0.8
Region 1	SSP3-7.0	2070s	119.5	140.0	0.6	1.1
Region 1	SSP3-7.0	2090s	132.9	148.6	0.6	1.2
Region 1	SSP5-8.5	2030s	75.2	76.6	0.3	0.6
Region 1	SSP5-8.5	2050s	91.1	107.5	0.4	0.8
Region 1	SSP5-8.5	2070s	127.0	146.1	0.6	1.1
Region 1	SSP5-8.5	2090s	130.3	144.3	0.6	1.1
Region 2	–	Current	5.6	8.2	0.0	0.1
Region 2	SSP1-2.6	2030s	28.0	35.2	0.1	0.2
Region 2	SSP1-2.6	2050s	33.1	38.3	0.1	0.2
Region 2	SSP1-2.6	2070s	45.0	42.0	0.1	0.3
Region 2	SSP1-2.6	2090s	57.3	46.8	0.2	0.3
Region 2	SSP2-4.5	2030s	23.3	29.8	0.1	0.2
Region 2	SSP2-4.5	2050s	42.6	42.9	0.1	0.3
Region 2	SSP2-4.5	2070s	63.4	48.9	0.2	0.3
Region 2	SSP2-4.5	2090s	91.2	56.3	0.2	0.4
Region 2	SSP3-7.0	2030s	22.6	29.7	0.1	0.2
Region 2	SSP3-7.0	2050s	45.9	41.9	0.1	0.3
Region 2	SSP3-7.0	2070s	86.1	51.8	0.2	0.3
Region 2	SSP3-7.0	2090s	127.2	80.7	0.3	0.5
Region 2	SSP5-8.5	2030s	28.3	36.4	0.1	0.2
Region 2	SSP5-8.5	2050s	54.0	45.4	0.1	0.3
Region 2	SSP5-8.5	2070s	111.6	64.6	0.2	0.4
Region 2	SSP5-8.5	2090s	119.6	95.4	0.3	0.6
Region 3	–	Current	190.1	128.3	0.4	0.8
Region 3	SSP1-2.6	2030s	224.0	115.3	0.4	0.7

Region 3	SSP1-2.6	2050s	222.9	109.8	0.3	0.7
Region 3	SSP1-2.6	2070s	234.8	117.6	0.4	0.7
Region 3	SSP1-2.6	2090s	233.7	106.3	0.3	0.7
Region 3	SSP2-4.5	2030s	220.4	111.4	0.4	0.7
Region 3	SSP2-4.5	2050s	232.4	114.7	0.4	0.7
Region 3	SSP2-4.5	2070s	235.9	112.1	0.4	0.7
Region 3	SSP2-4.5	2090s	229.7	110.3	0.4	0.7
Region 3	SSP3-7.0	2030s	221.3	116.6	0.4	0.7
Region 3	SSP3-7.0	2050s	218.9	117.5	0.4	0.7
Region 3	SSP3-7.0	2070s	226.4	113.0	0.4	0.7
Region 3	SSP3-7.0	2090s	199.4	120.9	0.4	0.8
Region 3	SSP5-8.5	2030s	231.7	115.2	0.4	0.7
Region 3	SSP5-8.5	2050s	235.7	121.0	0.4	0.8
Region 3	SSP5-8.5	2070s	206.1	112.9	0.4	0.7
Region 3	SSP5-8.5	2090s	149.5	129.3	0.4	0.8
Region 4	–	Current	34.4	43.0	0.1	0.2
Region 4	SSP1-2.6	2030s	86.0	63.1	0.1	0.3
Region 4	SSP1-2.6	2050s	100.7	66.1	0.1	0.3
Region 4	SSP1-2.6	2070s	103.0	65.7	0.1	0.3
Region 4	SSP1-2.6	2090s	108.8	67.1	0.1	0.3
Region 4	SSP2-4.5	2030s	82.5	60.1	0.1	0.2
Region 4	SSP2-4.5	2050s	103.8	67.9	0.1	0.3
Region 4	SSP2-4.5	2070s	135.7	71.6	0.1	0.3
Region 4	SSP2-4.5	2090s	144.3	76.6	0.2	0.3
Region 4	SSP3-7.0	2030s	77.7	60.6	0.1	0.2
Region 4	SSP3-7.0	2050s	109.8	67.7	0.1	0.3
Region 4	SSP3-7.0	2070s	136.2	74.1	0.2	0.3
Region 4	SSP3-7.0	2090s	151.1	99.7	0.2	0.4
Region 4	SSP5-8.5	2030s	91.6	64.1	0.1	0.3
Region 4	SSP5-8.5	2050s	116.7	70.1	0.1	0.3
Region 4	SSP5-8.5	2070s	145.6	85.2	0.2	0.3
Region 4	SSP5-8.5	2090s	131.0	111.1	0.2	0.5
Region 5	–	Current	36.7	68.5	0.1	0.3
Region 5	SSP1-2.6	2030s	56.6	89.3	0.2	0.3
Region 5	SSP1-2.6	2050s	58.4	89.4	0.2	0.3
Region 5	SSP1-2.6	2070s	64.7	96.4	0.2	0.4
Region 5	SSP1-2.6	2090s	68.4	101.8	0.2	0.4
Region 5	SSP2-4.5	2030s	58.6	93.9	0.2	0.3
Region 5	SSP2-4.5	2050s	59.2	89.7	0.2	0.3
Region 5	SSP2-4.5	2070s	75.6	107.7	0.2	0.4
Region 5	SSP2-4.5	2090s	84.6	119.6	0.2	0.4
Region 5	SSP3-7.0	2030s	54.2	86.8	0.2	0.3
Region 5	SSP3-7.0	2050s	63.8	95.2	0.2	0.3

Region 5	SSP3-7.0	2070s	77.0	108.7	0.2	0.4
Region 5	SSP3-7.0	2090s	98.5	134.0	0.3	0.5
Region 5	SSP5-8.5	2030s	57.7	92.0	0.2	0.3
Region 5	SSP5-8.5	2050s	66.9	97.8	0.2	0.4
Region 5	SSP5-8.5	2070s	87.5	121.3	0.2	0.4
Region 5	SSP5-8.5	2090s	111.7	143.7	0.3	0.5

Note. spgc = species per grid cell. Region 1: European Russia-West Siberia, Region 2: East Siberia, Region 3: Beringia, Region 4: Canada, and Region 5: North Atlantic.

Table S7. The number of species and extinction risk in the Arctic and Arctic floristic sectors in the future under four GHG emission scenarios

Region	SSPs	Period	No. species with range expansion	No. species with range contraction	No. existing species
All	SSP1-2.6	2030s	1043	73	1116
All	SSP1-2.6	2050s	1020	96	1115
All	SSP1-2.6	2070s	1056	71	1127
All	SSP1-2.6	2090s	1064	77	1141
All	SSP2-4.5	2030s	1047	70	1117
All	SSP2-4.5	2050s	1039	87	1126
All	SSP2-4.5	2070s	1058	84	1142
All	SSP2-4.5	2090s	1046	102	1147
All	SSP3-7.0	2030s	1029	88	1117
All	SSP3-7.0	2050s	1029	102	1131
All	SSP3-7.0	2070s	1039	109	1147
All	SSP3-7.0	2090s	1022	133	1155
All	SSP5-8.5	2030s	1048	70	1118
All	SSP5-8.5	2050s	1042	95	1137
All	SSP5-8.5	2070s	1023	129	1152
All	SSP5-8.5	2090s	952	200	1151
Region 1	SSP1-2.6	2030s	667	153	796
Region 1	SSP1-2.6	2050s	686	168	824
Region 1	SSP1-2.6	2070s	762	161	892
Region 1	SSP1-2.6	2090s	795	163	917
Region 1	SSP2-4.5	2030s	665	165	808
Region 1	SSP2-4.5	2050s	760	163	899
Region 1	SSP2-4.5	2070s	790	182	921
Region 1	SSP2-4.5	2090s	768	219	919
Region 1	SSP3-7.0	2030s	625	154	756
Region 1	SSP3-7.0	2050s	787	147	906
Region 1	SSP3-7.0	2070s	779	210	927
Region 1	SSP3-7.0	2090s	718	267	859
Region 1	SSP5-8.5	2030s	721	121	828
Region 1	SSP5-8.5	2050s	765	176	900
Region 1	SSP5-8.5	2070s	738	249	887
Region 1	SSP5-8.5	2090s	662	306	793
Region 2	SSP1-2.6	2030s	458	55	488
Region 2	SSP1-2.6	2050s	485	57	515
Region 2	SSP1-2.6	2070s	548	50	570
Region 2	SSP1-2.6	2090s	656	46	678

Region 2	SSP2-4.5	2030s	442	56	472
Region 2	SSP2-4.5	2050s	521	50	543
Region 2	SSP2-4.5	2070s	647	47	672
Region 2	SSP2-4.5	2090s	743	39	763
Region 2	SSP3-7.0	2030s	443	55	476
Region 2	SSP3-7.0	2050s	537	53	560
Region 2	SSP3-7.0	2070s	728	37	749
Region 2	SSP3-7.0	2090s	834	31	849
Region 2	SSP5-8.5	2030s	472	50	499
Region 2	SSP5-8.5	2050s	594	49	620
Region 2	SSP5-8.5	2070s	789	35	806
Region 2	SSP5-8.5	2090s	887	43	909
Region 3	SSP1-2.6	2030s	851	205	1052
Region 3	SSP1-2.6	2050s	797	257	1045
Region 3	SSP1-2.6	2070s	853	208	1057
Region 3	SSP1-2.6	2090s	831	236	1058
Region 3	SSP2-4.5	2030s	802	242	1035
Region 3	SSP2-4.5	2050s	838	237	1069
Region 3	SSP2-4.5	2070s	825	268	1087
Region 3	SSP2-4.5	2090s	807	309	1110
Region 3	SSP3-7.0	2030s	874	198	1068
Region 3	SSP3-7.0	2050s	793	293	1081
Region 3	SSP3-7.0	2070s	792	317	1102
Region 3	SSP3-7.0	2090s	727	408	1124
Region 3	SSP5-8.5	2030s	881	180	1057
Region 3	SSP5-8.5	2050s	831	272	1098
Region 3	SSP5-8.5	2070s	749	382	1122
Region 3	SSP5-8.5	2090s	620	518	1110
Region 4	SSP1-2.6	2030s	599	52	642
Region 4	SSP1-2.6	2050s	649	47	688
Region 4	SSP1-2.6	2070s	671	46	706
Region 4	SSP1-2.6	2090s	713	48	752
Region 4	SSP2-4.5	2030s	582	58	632
Region 4	SSP2-4.5	2050s	670	44	706
Region 4	SSP2-4.5	2070s	741	42	769
Region 4	SSP2-4.5	2090s	813	49	847
Region 4	SSP3-7.0	2030s	570	59	622
Region 4	SSP3-7.0	2050s	680	42	713
Region 4	SSP3-7.0	2070s	780	52	820
Region 4	SSP3-7.0	2090s	901	65	946
Region 4	SSP5-8.5	2030s	629	47	667
Region 4	SSP5-8.5	2050s	722	47	761
Region 4	SSP5-8.5	2070s	854	63	898

Region 4	SSP5-8.5	2090s	897	91	969
Region 5	SSP1-2.6	2030s	1006	53	1059
Region 5	SSP1-2.6	2050s	956	105	1061
Region 5	SSP1-2.6	2070s	1019	65	1084
Region 5	SSP1-2.6	2090s	1043	56	1096
Region 5	SSP2-4.5	2030s	1027	38	1065
Region 5	SSP2-4.5	2050s	983	97	1077
Region 5	SSP2-4.5	2070s	1046	52	1094
Region 5	SSP2-4.5	2090s	1058	57	1112
Region 5	SSP3-7.0	2030s	998	59	1057
Region 5	SSP3-7.0	2050s	1009	75	1081
Region 5	SSP3-7.0	2070s	1034	77	1107
Region 5	SSP3-7.0	2090s	1044	74	1107
Region 5	SSP5-8.5	2030s	1011	52	1061
Region 5	SSP5-8.5	2050s	1009	81	1087
Region 5	SSP5-8.5	2070s	1048	70	1110
Region 5	SSP5-8.5	2090s	1009	110	1108

Note. All: Circumpolar Arctic, Region 1: European Russia-West Siberia, Region 2: East Siberia, Region 3: Beringia, Region 4: Canada, and Region 5: North Atlantic.

Table S8. The mean distributional centroid of species in the Arctic floristic sectors now and in the future under four GHG emission scenarios

Region	SSPs	Period	Latitude (°N)	Longitude（°）	Distance (km)
Region 1	–	Current	69.0	53.2	0.0
Region 1	SSP1-2.6	2030s	69.7	54.3	89.1
Region 1	SSP1-2.6	2050s	69.7	54.1	6.6
Region 1	SSP1-2.6	2070s	70.1	55.2	65.6
Region 1	SSP1-2.6	2090s	70.5	56.6	64.1
Region 1	SSP2-4.5	2030s	69.5	53.6	59.9
Region 1	SSP2-4.5	2050s	69.8	54.8	59.7
Region 1	SSP2-4.5	2070s	70.4	56.1	76.2
Region 1	SSP2-4.5	2090s	71.0	58.1	103.3
Region 1	SSP3-7.0	2030s	69.5	54.0	68.1
Region 1	SSP3-7.0	2050s	69.8	55.2	55.0
Region 1	SSP3-7.0	2070s	70.7	57.3	129.4
Region 1	SSP3-7.0	2090s	71.7	59.4	131.5
Region 1	SSP5-8.5	2030s	69.5	54.1	66.7
Region 1	SSP5-8.5	2050s	70.2	55.4	89.0
Region 1	SSP5-8.5	2070s	71.3	58.6	172.5
Region 1	SSP5-8.5	2090s	72.4	60.3	139.9
Region 2	–	Current	76.2	133.9	0.0
Region 2	SSP1-2.6	2030s	76.0	133.7	29.9
Region 2	SSP1-2.6	2050s	76.3	130.2	102.9
Region 2	SSP1-2.6	2070s	76.9	125.9	127.4
Region 2	SSP1-2.6	2090s	77.5	121.2	135.1
Region 2	SSP2-4.5	2030s	76.1	133.3	21.4
Region 2	SSP2-4.5	2050s	76.5	128.6	134.1
Region 2	SSP2-4.5	2070s	77.3	121.6	198.5
Region 2	SSP2-4.5	2090s	77.4	118.3	81.9
Region 2	SSP3-7.0	2030s	76.0	133.6	22.3
Region 2	SSP3-7.0	2050s	76.8	125.6	226.1
Region 2	SSP3-7.0	2070s	77.3	119.4	166.5
Region 2	SSP3-7.0	2090s	77.2	117.7	42.0
Region 2	SSP5-8.5	2030s	76.2	132.8	32.1
Region 2	SSP5-8.5	2050s	77.0	123.2	263.9
Region 2	SSP5-8.5	2070s	77.2	117.7	139.9
Region 2	SSP5-8.5	2090s	77.0	114.5	84.4
Region 3	–	Current	63.4	-161.0	0.0
Region 3	SSP1-2.6	2030s	63.9	-162.3	89.9
Region 3	SSP1-2.6	2050s	64.1	-162.7	24.8

Region 3	SSP1-2.6	2070s	64.1	-162.5	12.0
Region 3	SSP1-2.6	2090s	64.2	-163.2	34.7
Region 3	SSP2-4.5	2030s	63.9	-162.4	90.3
Region 3	SSP2-4.5	2050s	64.3	-162.9	47.4
Region 3	SSP2-4.5	2070s	64.5	-163.4	35.7
Region 3	SSP2-4.5	2090s	64.9	-164.0	49.1
Region 3	SSP3-7.0	2030s	63.8	-162.1	74.8
Region 3	SSP3-7.0	2050s	64.2	-162.4	40.8
Region 3	SSP3-7.0	2070s	64.9	-164.2	118.9
Region 3	SSP3-7.0	2090s	66.1	-166.6	171.4
Region 3	SSP5-8.5	2030s	64.0	-162.4	98.6
Region 3	SSP5-8.5	2050s	64.7	-163.4	88.9
Region 3	SSP5-8.5	2070s	65.5	-165.2	125.0
Region 3	SSP5-8.5	2090s	67.1	-168.6	234.6
Region 4	–	Current	73.9	-102.6	0.0
Region 4	SSP1-2.6	2030s	75.8	-96.5	271.7
Region 4	SSP1-2.6	2050s	76.1	-95.0	56.6
Region 4	SSP1-2.6	2070s	76.7	-93.5	78.9
Region 4	SSP1-2.6	2090s	77.2	-91.7	72.6
Region 4	SSP2-4.5	2030s	75.6	-96.2	266.4
Region 4	SSP2-4.5	2050s	76.6	-95.2	113.5
Region 4	SSP2-4.5	2070s	77.1	-90.8	124.3
Region 4	SSP2-4.5	2090s	78.0	-87.2	126.8
Region 4	SSP3-7.0	2030s	75.5	-97.5	237.1
Region 4	SSP3-7.0	2050s	76.4	-95.7	105.5
Region 4	SSP3-7.0	2070s	77.7	-89.2	218.8
Region 4	SSP3-7.0	2090s	79.4	-79.9	276.4
Region 4	SSP5-8.5	2030s	76.1	-96.2	303.7
Region 4	SSP5-8.5	2050s	77.0	-93.6	126.9
Region 4	SSP5-8.5	2070s	78.9	-82.9	323.7
Region 4	SSP5-8.5	2090s	79.9	-76.1	179.0
Region 5	–	Current	66.5	-48.2	0.0
Region 5	SSP1-2.6	2030s	67.1	-48.7	71.2
Region 5	SSP1-2.6	2050s	67.7	-48.6	68.6
Region 5	SSP1-2.6	2070s	69.0	-46.5	167.2
Region 5	SSP1-2.6	2090s	69.9	-45.2	108.2
Region 5	SSP2-4.5	2030s	66.9	-49.2	64.3
Region 5	SSP2-4.5	2050s	68.3	-46.6	192.9
Region 5	SSP2-4.5	2070s	69.4	-46.9	114.2
Region 5	SSP2-4.5	2090s	70.0	-46.4	67.0
Region 5	SSP3-7.0	2030s	67.0	-49.0	64.7
Region 5	SSP3-7.0	2050s	68.3	-47.4	165.5
Region 5	SSP3-7.0	2070s	70.1	-45.7	203.8

Region 5	SSP3-7.0	2090s	69.8	-48.3	105.1
Region 5	SSP5-8.5	2030s	67.0	-48.9	65.1
Region 5	SSP5-8.5	2050s	68.6	-47.3	194.0
Region 5	SSP5-8.5	2070s	70.0	-46.7	150.8
Region 5	SSP5-8.5	2090s	69.6	-49.9	134.1

Note. Region 1: European Russia-West Siberia, Region 2: East Siberia, Region 3: Beringia, Region 4: Canada, and Region 5: North Atlantic.

Table S9. The statistical results of the shifting direction of the distributional centroid for the sampled species in the future under four GHG emission scenarios

Region	SSPs	Period	Moving direction			
			Northeast	Northwest	Southwest	Southeast
Region 1	SSP1-2.6	2030s	35.7%	60.0%	1.0%	3.3%
Region 1	SSP2-4.5	2030s	35.4%	60.2%	1.3%	3.1%
Region 1	SSP3-7.0	2030s	33.6%	59.6%	1.1%	5.6%
Region 1	SSP5-8.5	2030s	36.5%	59.2%	1.3%	3.0%
Region 2	SSP1-2.6	2030s	2.7%	81.6%	4.2%	11.5%
Region 2	SSP2-4.5	2030s	2.4%	82.6%	4.0%	11.0%
Region 2	SSP3-7.0	2030s	2.7%	82.1%	4.4%	10.9%
Region 2	SSP5-8.5	2030s	3.0%	81.7%	4.0%	11.2%
Region 3	SSP1-2.6	2030s	13.9%	77.2%	5.9%	3.0%
Region 3	SSP2-4.5	2030s	12.8%	76.2%	7.3%	3.6%
Region 3	SSP3-7.0	2030s	14.2%	77.2%	5.5%	3.2%
Region 3	SSP5-8.5	2030s	13.5%	78.6%	5.6%	2.3%
Region 4	SSP1-2.6	2030s	22.6%	65.1%	6.8%	5.5%
Region 4	SSP2-4.5	2030s	23.0%	64.6%	6.7%	5.7%
Region 4	SSP3-7.0	2030s	21.8%	66.0%	6.6%	5.6%
Region 4	SSP5-8.5	2030s	21.9%	65.3%	7.1%	5.7%
Region 5	SSP1-2.6	2030s	29.2%	45.1%	18.4%	7.3%
Region 5	SSP2-4.5	2030s	30.2%	49.4%	17.4%	2.9%
Region 5	SSP3-7.0	2030s	30.2%	44.4%	19.4%	6.0%
Region 5	SSP5-8.5	2030s	34.0%	43.3%	16.9%	5.8%
Region 1	SSP1-2.6	2050s	29.2%	43.5%	7.2%	20.1%
Region 1	SSP2-4.5	2050s	50.2%	38.6%	2.2%	9.0%
Region 1	SSP3-7.0	2050s	49.2%	41.3%	2.0%	7.5%
Region 1	SSP5-8.5	2050s	51.5%	39.8%	3.2%	5.6%
Region 2	SSP1-2.6	2050s	4.9%	82.4%	4.2%	8.5%
Region 2	SSP2-4.5	2050s	3.4%	79.5%	6.4%	10.7%
Region 2	SSP3-7.0	2050s	3.3%	81.4%	6.9%	8.4%
Region 2	SSP5-8.5	2050s	2.8%	80.4%	7.6%	9.3%
Region 3	SSP1-2.6	2050s	19.7%	54.6%	17.7%	8.0%
Region 3	SSP2-4.5	2050s	25.8%	67.0%	5.0%	2.3%
Region 3	SSP3-7.0	2050s	26.0%	56.8%	10.4%	6.7%
Region 3	SSP5-8.5	2050s	27.4%	64.7%	4.5%	3.4%
Region 4	SSP1-2.6	2050s	15.7%	61.2%	13.0%	10.2%
Region 4	SSP2-4.5	2050s	16.1%	65.5%	11.3%	7.1%
Region 4	SSP3-7.0	2050s	23.3%	58.8%	10.2%	7.8%
Region 4	SSP5-8.5	2050s	29.0%	54.4%	10.7%	5.9%
Region 5	SSP1-2.6	2050s	42.0%	25.4%	16.6%	16.0%

Region 5	SSP2-4.5	2050s	54.0%	20.2%	9.5%	16.3%
Region 5	SSP3-7.0	2050s	52.7%	24.9%	10.4%	12.0%
Region 5	SSP5-8.5	2050s	55.2%	29.8%	7.1%	7.9%
Region 1	SSP1-2.6	2070s	52.1%	37.8%	2.3%	7.8%
Region 1	SSP2-4.5	2070s	53.7%	34.9%	3.3%	8.1%
Region 1	SSP3-7.0	2070s	56.8%	37.1%	2.1%	4.0%
Region 1	SSP5-8.5	2070s	57.0%	36.7%	1.4%	4.8%
Region 2	SSP1-2.6	2070s	4.0%	77.5%	8.9%	9.5%
Region 2	SSP2-4.5	2070s	7.7%	73.5%	7.2%	11.7%
Region 2	SSP3-7.0	2070s	5.7%	72.3%	9.5%	12.5%
Region 2	SSP5-8.5	2070s	7.8%	68.9%	11.8%	11.5%
Region 3	SSP1-2.6	2070s	25.9%	40.4%	18.8%	15.0%
Region 3	SSP2-4.5	2070s	20.8%	65.6%	6.9%	6.7%
Region 3	SSP3-7.0	2070s	13.4%	79.4%	4.5%	2.8%
Region 3	SSP5-8.5	2070s	16.6%	73.9%	6.7%	2.9%
Region 4	SSP1-2.6	2070s	26.5%	59.1%	5.6%	8.8%
Region 4	SSP2-4.5	2070s	29.0%	51.6%	9.3%	10.1%
Region 4	SSP3-7.0	2070s	33.3%	53.2%	7.6%	5.9%
Region 4	SSP5-8.5	2070s	34.5%	49.7%	10.3%	5.5%
Region 5	SSP1-2.6	2070s	57.0%	27.9%	9.5%	5.6%
Region 5	SSP2-4.5	2070s	41.8%	39.4%	16.3%	2.5%
Region 5	SSP3-7.0	2070s	57.7%	28.5%	10.7%	3.1%
Region 5	SSP5-8.5	2070s	53.7%	30.6%	13.5%	2.2%
Region 1	SSP1-2.6	2090s	52.3%	31.8%	5.1%	10.7%
Region 1	SSP2-4.5	2090s	57.6%	32.6%	3.3%	6.5%
Region 1	SSP3-7.0	2090s	53.3%	38.1%	2.8%	5.8%
Region 1	SSP5-8.5	2090s	45.2%	47.3%	1.9%	5.6%
Region 2	SSP1-2.6	2090s	7.3%	70.2%	8.6%	13.9%
Region 2	SSP2-4.5	2090s	8.0%	66.2%	10.8%	15.0%
Region 2	SSP3-7.0	2090s	12.8%	59.1%	10.4%	17.6%
Region 2	SSP5-8.5	2090s	19.2%	46.8%	15.7%	18.4%
Region 3	SSP1-2.6	2090s	9.3%	62.1%	18.1%	10.5%
Region 3	SSP2-4.5	2090s	22.5%	66.6%	5.3%	5.6%
Region 3	SSP3-7.0	2090s	19.4%	74.4%	4.4%	1.9%
Region 3	SSP5-8.5	2090s	17.8%	74.6%	4.0%	3.7%
Region 4	SSP1-2.6	2090s	21.3%	58.8%	8.7%	11.2%
Region 4	SSP2-4.5	2090s	29.1%	52.8%	12.2%	5.8%
Region 4	SSP3-7.0	2090s	36.9%	47.4%	11.6%	4.0%
Region 4	SSP5-8.5	2090s	36.0%	43.4%	14.9%	5.7%
Region 5	SSP1-2.6	2090s	61.3%	21.1%	11.6%	5.9%
Region 5	SSP2-4.5	2090s	55.3%	25.9%	15.6%	3.1%
Region 5	SSP3-7.0	2090s	35.8%	33.5%	28.2%	2.4%
Region 5	SSP5-8.5	2090s	38.4%	27.0%	32.3%	2.3%

Note. Region 1: European Russia-West Siberia, Region 2: East Siberia, Region 3: Beringia, Region 4: Canada, and Region 5: North Atlantic.

Table S10. Pearson correlation coefficients (*r*) for the 9 bioclimate variables

Bioclimatic variables	bio1	bio2	bio3	bio5	bio7	bio8	bio13	bio15	bio17
bio1	1	-0.428	0.516	0.666	-0.787	0.117	-0.039	-0.72	0.343
bio2		1	0.201	0.23	0.754	0.274	-0.157	0.389	-0.341
bio3			1	0.342	-0.433	-0.022	0.066	-0.458	0.342
bio5				1	-0.082	0.481	-0.334	-0.345	-0.125
bio7					1	0.222	-0.181	0.707	-0.536
bio8						1	-0.395	0.108	-0.518
bio13							1	0.159	0.795
bio15								1	-0.394
bio17									1

Note. bio1 = Annual Mean Temperature; bio2 = Mean Diurnal Range (Mean of monthly (max temp - min temp)); bio3 = Isothermality (bio2/bio7) (* 100); bio4 = Temperature Seasonality (standard deviation *100); bio5 = Max Temperature of Warmest Month; bio6 = Min Temperature of Coldest Month; bio7 = Temperature Annual Range (bio5-bio6); bio8 = Mean Temperature of Wettest Quarter; bio9 = Mean Temperature of Driest Quarter; bio10 = Mean Temperature of Warmest Quarter; bio11 = Mean Temperature of Coldest Quarter; bio12 = Annual Precipitation; bio13 = Precipitation of Wettest Month; bio14 = Precipitation of Driest Month; bio15 = Precipitation Seasonality (Coefficient of Variation); bio16 = Precipitation of Wettest Quarter; bio17 = Precipitation of Driest Quarter; bio18 = Precipitation of Warmest Quarter; bio19 = Precipitation of Coldest Quarter.

References

1. Seddon AWR, Macias-Fauria M, Long PR *et al.* Sensitivity of global terrestrial ecosystems to climate variability. *Nature* 2016;**531**:229–32.
2. Zhou W, Leung LR, Lu J. Steady threefold Arctic amplification of externally forced warming masked by natural variability. *Nat Geosci* 2024;**17**:508–15.
3. Cohen J, Screen JA, Furtado JC *et al.* Recent Arctic amplification and extreme mid-latitude weather. *Nat Geosci* 2014; **7**: 627–37.
4. Schuur EAG, Abbott BW, Commane R *et al.* Permafrost and Climate Change: Carbon Cycle Feedbacks From the Warming Arctic.
5. Masson-Delmotte V, Zhai P, Pirani Aet al. IPCC, *Climate Change 2021: The Physical Science Basis. Contribution of Working Group I to the Sixth Assessment Report of the Intergovernmental Panel on Climate Change*. Basel: Cambridge University Press, 2021, 583–4.
6. Foster GL, Royer DL, Lunt DJ. Future climate forcing potentially without precedent in the last 420 million years. *Nat Commun* 2017;**8**:14845.
7. Chen I-C, Hill JK, Ohlemüller R *et al.* Rapid range shifts of species associated with high levels of climate warming. *Science* 2011;**333**:1024–6.
8. Scheffers BR, De Meester L, Bridge TCL *et al.* The broad footprint of climate change from genes to biomes to people. *Science* 2016;**354**:aaf7671.
9. Elven R, Murray DF, Razzhivin VY, *et al. Annotated Checklist of the Panarctic Flora (PAF): Vascular plants version 1.0.* 2011, (http: //nhm2.uio.no/paf, accessed February 2022).
10. Stubbs RL, Soltis DE, Cellinese N. The future of cold-adapted plants in changing climates: *Micranthes* (Saxifragaceae) as a case study. *Ecol Evol* 2018;**8**:7164–77.

11. Oke TA, Stralberg D, Reid DG *et al.* Warming drives poleward range contractions of Beringian endemic plant species at high latitudes. *Divers Distrib* 2023;**29**:509–23.

12. Niskanen AKJ, Niittynen P, Aalto J *et al.* Lost at high latitudes: Arctic and endemic plants under threat as climate warms. Diez J (ed.). *Divers Distrib* 2019;**25**:809–21.

13. Walker DA, Raynolds MK, Daniëls FJA *et al.* The Circumpolar Arctic vegetation map. *J Veg Sci* 2005; **16**: 267–82.

14. Myers-Smith IH, Kerby JT, Phoenix GK *et al.* Complexity revealed in the greening of the Arctic. *Nat Clim Change* 2020; **10**: 106–17.

15. Storch D, Keil P, Jetz W. Universal species–area and endemics–area relationships at continental scales. *Nature* 2012;**488**:78–81.

16. Araújo MB, Anderson RP, Márcia Barbosa A *et al*. Standards for distribution models in biodiversity assessments. *Sci Adv* 2019; **5**: eaat4858.

17. Feng X, Peterson AT, Aguirre-López LJ *et al*. Rethinking ecological niches and geographic distributions in face of pervasive human influence in the Anthropocene. *Biol Rev* 2024; **99**: 1481–503.

18. Ives JD, Barry RG eds. *The Arctic: Environment, People, Policy*. London: Routledge, 2019.

19. Raynolds MK, Walker DA, Balser A *et al.* A raster version of the Circumpolar Arctic Vegetation Map (CAVM). *Remote Sens Environ* 2019;**232**:111297.

20. Proosdij ASJ, Sosef MSM, Wieringa JJ *et al.* Minimum required number of specimen records to develop accurate species distribution models. *Ecography* 2016;**39**:542–52.

21. Hausfather Z, Peters GP. Emissions – the 'business as usual' story is misleading. *Nature* 2020;**577**:618–20.

22. Su B, Huang J, Mondal SK *et al.* Insight from CMIP6 SSP-RCP scenarios for future drought

characteristics in China. *Atmospheric Res* 2021;**250**:105375.

23. Voldoire A, Saint-Martin D, Sénési S *et al.* Evaluation of CMIP6 DECK Experiments With CNRM-CM6-1. *J Adv Model Earth Syst* 2019;**11**:2177–213.
24. Graham MH. Confronting multicollinearity in ecological multiple regression. *Ecology* 2003;**84**:2809–15.
25. R Core Team. *R: A Language and Environment for Statistical Computing* (R Foundation for Statistical Computing, 2021); http://www.R-project.org/.
26. Thuiller W, Georges D, Gueguen M *et al.* biomod2: Ensemble Platform for Species Distribution Modeling. 2025.
27. Araujo M, New M. Ensemble forecasting of species distributions. *Trends Ecol Evol* 2007;**22**:42–7.
28. Allouche O, Tsoar A, Kadmon R. Assessing the accuracy of species distribution models: prevalence, kappa and the true skill statistic (TSS). *J Appl Ecol* 2006;**43**:1223–32.
29. Swets JA. http://www.jstor.org Measuring the Accuracy of Diagnostic Systems. *Sci New Ser* 1988;**240**:1285–93.
30. Thuiller W, Lafourcade B, Engler R *et al.* BIOMOD – a platform for ensemble forecasting of species distributions. *Ecography* 2009;**32**:369–73.
31. Liu C, Newell G, White M. On the selection of thresholds for predicting species occurrence with presence-only data. *Ecol Evol* 2016;**6**:337–48.
32. Graham CH, Hijmans RJ. A comparison of methods for mapping species ranges and species richness: Mapping species ranges and species richness. *Glob Ecol Biogeogr* 2006;**15**:578–87.
33. Conservation of Arctic Flora and Fauna (CAFF). *Arctic Biodiversity Assessment: Status and*

Trends in Arctic Biodiversity. Arctic Council, 2013.

Data S1.

Occurrence records and AUC value in ensemble modelling results of 1,187 Arctic species.

AUC: area under the receiver operating characteristic curve.

Data S2.

Species' Area of Habitat (AOH) in the Arctic from now to the 2090s (2030s, 2050s, 2070s, and 2090s) under four GHG emission scenarios (SSP1-2.6, SSP2-4.5, SSP3-7.0, and SSP5-8.5).

Data S3.

Species' Area of Habitat (AOH) in the five Arctic floristic sectors from now to the 2090s under four GHG emission scenarios. Region 1: European Russia-West Siberia, Region 2: East Siberia, Region 3: Beringia, Region 4: Canada, and Region 5: North Atlantic. NA: not applicable.
